# Shedding Light on the Volatile Composition of *Broa*, a Traditional Portuguese Maize Bread

**DOI:** 10.3390/biom11101396

**Published:** 2021-09-22

**Authors:** Andreia Bento-Silva, Noélia Duarte, Maria Belo, Elsa Mecha, Bruna Carbas, Carla Brites, Maria Carlota Vaz Patto, Maria Rosário Bronze

**Affiliations:** 1 FCT NOVA, Faculdade de Ciências e Tecnologia, Campus da Caparica, Universidade Nova de Lisboa, 2829-516 Caparica, Portugal; abentosilva@ff.ulisboa.pt; 2 ITQB NOVA, Instituto de Tecnologia Química e Biológica António Xavier, Universidade Nova de Lisboa, Avenida da República, 2780-157 Oeiras, Portugal; mariabelo87@gmail.com (M.B.); emecha@itqb.unl.pt (E.M.); cpatto@itqb.unl.pt (M.C.V.P.); 3 DCFM, Departamento de Ciências Farmacêuticas e do Medicamento, Faculdade de Farmácia da Universidade de Lisboa, Av. das Forças Armadas, 1649-003 Lisboa, Portugal; 4 iMed.ULisboa, Instituto de Investigação do Medicamento, Faculdade de Farmácia, Universidade de Lisboa, Avenida Prof. Gama Pinto, 1649-003 Lisboa, Portugal; mduarte@ff.ulisboa.pt; 5 INIAV, Instituto Nacional de Investigação Agrária e Veterinária, Avenida da República, Quinta do Marquês, 2780-157 Oeiras, Portugal; bruna.carbas@iniav.pt (B.C.); carla.brites@iniav.pt (C.B.); 6 iBET, Instituto de Biologia Experimental e Tecnológica, Avenida da República, Quinta do Marquês, Estação Agronómica Nacional, Apartado 12, 2780-157 Oeiras, Portugal

**Keywords:** maize, *broa*, maize bread, volatiles, phenolic compounds, Maillard reaction

## Abstract

In Portugal, maize has been used for centuries to produce an ethnic bread called *broa*, employing traditional maize varieties, which are preferred by the consumers in detriment of commercial hybrids. In order to evaluate the maize volatiles that can influence consumers’ acceptance of *broas*, twelve *broas* were prepared from twelve maize varieties (eleven traditional and one commercial hybrid), following a traditional recipe. All maize flours and *broas* were analyzed by HS-SPME-GC-MS (headspace solid-phase microextraction) and *broas* were appraised by a consumer sensory panel. In addition, the major soluble phenolics and total carotenoids contents were quantitated in order to evaluate their influence as precursors or inhibitors of volatile compounds. Results showed that the major volatiles detected in maize flours and *broas* were aldehydes and alcohols, derived from lipid oxidation, and some ketones derived from carotenoids’ oxidation. Both lipid and carotenoids’ oxidation reactions appeared to be inhibited by soluble phenolics. In contrast, phenolic compounds appeared to increase browning reactions during bread making and, consequently, the production of pyranones. Traditional samples, especially those with higher contents in pyranones and lower contents in aldehydes, were preferred by the consumer sensory panel. These findings suggest that, without awareness, consumers prefer *broas* prepared from traditional maize flours with higher contents in health-promoting phenolic compounds, reinforcing the importance of preserving these valuable genetic resources.

## 1. Introduction

The culture of maize in Portugal started during the sixteenth century and rapidly spread across the country. For centuries, natural and human selection adapted varieties to different environments, creating a diverse maize germplasm [1]. Portuguese maize traditional open pollinated varieties (OPVs) are commonly used for the production of *broa*, a traditional Portuguese maize bread [2], which plays an important economic and social role in Central and Northern Portuguese rural communities, where it is still widely consumed [1]. This type of bread is traditionally prepared with maize flour (50–100%) and rye and/or wheat flours (0–50%) that are mixed with hot water, and leavened dough from late *broa*, acting as sourdough [2,3]. After preparation, dough is baked in a wood-fired oven [3]. Due to the progressive adoption of more productive hybrids, Portuguese traditional maize varieties are at risk of disappearing [1]. Nevertheless, traditional varieties have persisted due to their better technological capacity and aroma characteristics highly valued for the production of *broa*, in addition to their better resilience to pests, diseases and abiotic stresses, qualities exploited by the Portuguese long-term participatory maize breeding VASO program [4]. In fact, previously reported sensory evaluation results of *broas* have demonstrated a preference for traditional or participatory improved OPVs in detriment of commercial hybrid maize varieties for *broa* production [3]. Additionally, traditional maize varieties still under production have an important role in the conservation and evolution of the available genetic maize variation, meeting the future demands for increasing yields and quality in high-stress environments in the context of climate changes [4].

Quality criteria important to consumers’ acceptance of breads are related to both their rheological (texture) and organoleptic (color, volume and flavor) properties [3,5,6]. Flavor is often considered the most important attribute [7,8,9] and results, in part, from the perception of volatile compounds that can interact with olfactory receptors [6]. The volatile composition of breads depends on the ingredients and processing techniques employed, and mainly results from oxidation of lipids and carotenoids, fermentation by yeast and lactic acid bacteria (LAB) and browning reactions (Maillard and caramelization reactions) during baking [10,11]. Aldehydes, alcohols, ketones, esters, acids, pyrazines, pyrrolines, hydrocarbons, furans and lactones [12] have been described as the main volatiles present in cereal breads, but the exact origin of each volatile is difficult to determine [13]. For instance, aldehydes, alcohols and furans may all be derived from lipid oxidation, yeast fermentation or Maillard and caramelization reactions [14,15,16].

In general, lipid oxidation products are associated with off-flavors [17,18] and are produced by plants through the action of lipoxygenases in response to wounding, thus playing an important role in plants’ defense strategies and signaling [7,19]. Flavor development during bread making is influenced by lipoxygenase activity [11] and by the type of sugars and amino acids present in the flour [19]. Lipoxygenases act on unsaturated fatty acids and produce unstable peroxide derivatives that can be transformed into carbonyl compounds, such as aldehydes [11,16], which can be enzymatically reduced to the corresponding alcohols [7] or be oxidized into acids and esters [16,17,20]. 4- or 5-Hydroxy carboxylic acids may further be converted to lactones [7,17], and hydrocarbons can be formed through the oxidation of the radical to the carbonium ion and decarboxylation [21]. The activity of endogenous lipoxygenases increases during grinding of the grain, after the addition of water to the flour and during kneading [15,16]. Additionally, lipid oxidation reactions may also occur by the action of enzymes associated with the metabolic activity of yeasts and LAB during fermentation [6]. 

Apart from lipid oxidation, aldehydes can also be formed inside the yeast cells, by the degradation of flour amino acids via the Ehrlich pathway, and originate the corresponding alcohols, acids and esters [16,17,20]. Different volatile compounds can be formed, depending on the LAB and yeasts present in the dough [11,16,22,23]. Ketones, such as geranylacetone, can be originated from carotenoids’ oxidation, which have been described in maize baked products and other cereal breads [11,24].

In addition to color development, browning reactions produce compounds that contribute to the flavor of bakery products [5]. In particular, Maillard products are formed through a reaction between amino acids and reducing sugars, leading to the formation of brown pigments (melanoidins), and a large number of volatile compounds [5,6,20]. This process consists of mainly three stages: (1) sugar degradation, where compounds such as furans, pyrones, furfurals, furanones and pyranones are formed, followed by (2) amino acids’ degradation (Strecker reaction), which generates mainly aldehydes, acids and alcohols, and (3) further interactions, originating colored melanoidins and other volatiles, such as pyrroles, pyridines and pyrazines [25]. These volatiles are considered important for the overall aroma profile of breads, as they generally have low odor thresholds and contribute to the desirable aroma properties [11,17,19,20,26]. Caramelization reactions are originated through sugars’ degradation in the absence of amino acids and provide compounds especially related to caramel flavor [5,19]. Low levels of volatiles from non-enzymatic browning reactions and high levels of lipid oxidation products contribute to the formation of off-flavors in breads [18].

Some studies have reported that variations in wheat flour odor directly affect bread flavor [23,27]. Moreover, phenolic compounds may also contribute to the volatile composition of foods, influencing both the Maillard and lipid oxidation reactions [28,29,30,31]. In particular, the presence of ferulic acid in whole wheat breads has been reported as the main reason for the difference in the aroma of breads prepared from whole and refined wheat flours [28]. As ferulic acid is particularly abundant in maize, where it can be at least 10 times higher than in other grains [32], it is expected that the volatile composition of *broas* may be especially affected by their phenolic composition. Additionally, these compounds exhibit antioxidant properties, contributing to the prevention of non-communicable diseases [33] that are generally regarded as a major public health concern.

Several studies have been published on the identification of key volatiles responsible for odor quality in maize-based foods, such as popcorn and cornflakes [24,34,35,36,37]. However, to the best of our knowledge, the volatile composition of maize-based sourdough breads has never been studied. The main objectives of this work were to: (1) characterize *broas*’ volatile composition in order to identify volatiles that may influence consumer’s choice, and to (2) shed light on the characteristics of traditional maize varieties responsible for their better suitability for *broas* production. In particular, the influence of (2a) soluble phenolic compounds, (2b) total carotenoids content and (2c) volatiles from maize flours on *broas’* volatile composition was evaluated. This knowledge is important to both the baking industry and maize breeders, as cereals’ composition contribution to odor can possibly become a future quality parameter in breeding [27].

## 2. Materials and Methods

### 2.1. Maize Flours and Broas Preparation

Eleven traditional or participatory improved open pollinated Portuguese maize varieties (F1 to F11, from now on referred to only as traditional varieties, since the participatory improved were also derived from traditional varieties) and a commercial hybrid maize flour—Nacional Type 175 (F12)—were studied (Table 1). The traditional varieties’ samples are representative of the national maize germplasm variability, taking into account their agronomic performance in field trials, basic nutritional quality and genetic diversity evaluated under the scope of the FP7 SOLIBAM European project, and were described in previous studies [38]. All maize samples were obtained from field trials conducted at ESAC (Escola Superior Agrária de Coimbra, Coimbra, Portugal). Flours were obtained after milling the whole maize grain in an artisan water mill with millstones (Moinhos do Inferno, Viseu, Portugal), with the exception of the commercial hybrid sample (F12), which was acquired already milled.

Twelve *broas* (B1 to B12) were prepared in a bakery following a traditional recipe. The procedure was previously described [39]. The ingredients included 70% maize flour, 20% commercial rye flour (Concordia type 70, Portugal) and 10% commercial wheat flour (National type 65, Portugal). Flours were mixed with 80% boiling water (vol/wt, flour basis) containing 1.76% salt and kneaded for 5 min (Ferneto AEF035). The dough was allowed to rest and cool to 27 °C, and the remaining ingredients (sugar, salt, dry yeast, sourdough) and 20% water were added. The dough was again kneaded for 8 min and left to rest for bulk fermentation at 25 °C for 90 min. After fermentation, the dough was manually molded into 400 g balls and baked in the oven (Matador, Werner & Pfleiderer Lebensmitteltechnik GmbH, Dinkelsbühl, Germany) at 270 °C for 40 min.

The commercial rye and wheat flours used for *broas* preparation were acquired already milled and analyzed in the same conditions.

### 2.2. HS-SPME-GC-MS Analysis

#### 2.2.1. Materials and Equipment

The different SPME fibers used (PDMS, PDMS/DVB, PA and DVB/CAR/PDMS) were purchased from Supelco (Sigma-Aldrich, St. Louis, MO, USA). The alkane standard mixture C_8_-C_20_ solution (40 mg L^−1^ in hexane) was purchased from Fluka (Fisher Scientific, Hampton, NH, USA). Ultra-pure water (18.2 MΩ.cm^−1^) was obtained from a Millipore-Direct equipment Q3 UV system (Millipore, Burlington, MA, USA). GC vials were sealed with a silicone/teflon-lined septum and screw cap (La-Pha-Pack, Langerwehe, Germany).

The analysis of volatile compounds was carried out using a GCMS-QP2010 Plus mass spectrometer (Shimadzu, Kyoto, Japan) and an AOC-5000 autosampler (Shimadzu, Kyoto, Japan). The compounds were separated using a Varian Factor Four DB-5MS column (30.0 m × 0.25 mm × 0.25 µm film thicknesses, Agilent J&W, Santa Clara, CA, USA).

#### 2.2.2. HS-SPME Optimization of Cereal Flours

The commercial maize flour was used to optimize the HS-SPME extraction of volatile compounds from cereal flours. Different ratios of flour:water were studied (addition of 0, 2, 5, 8 and 10 mL of water to 2 g of flour), as well as different types of fibers (DVB/CAR/PDMS 50/30 μm, PDMS 100 μm, PDMS/DVB 65 μm and PA 85 μm), temperatures (40, 50, 60 and 70 °C) and times (10, 20, 30, 40, 50 and 60 min) of extraction, in triplicate. Additionally, sample stability in the autosampler was evaluated for 32 h. The selected conditions were also applied to the commercial wheat and rye flours. 

#### 2.2.3. HS-SPME of Volatile Compounds of Cereal Flours and *broas*

After optimization, the following conditions were used for the volatile analysis of cereal flours (maize, wheat and rye): flour (2 g) was placed in a 20 mL GC-vial, deionized water (5 mL) was added and the mixture was vortexed for 2 min before HS-SPME-GC-MS analysis. For *broas*, 4 g of sample (whole, crumb and crust) was smashed manually and placed in a 20 mL vial. A 50/30 µm DVB/CARBOXEN/PDMS fiber was selected to concentrate the volatile compounds. Samples were extracted for 40 min at 60 °C and 250 rpm. Before extraction, the fiber was conditioned in the GC injector at 270 °C for 30 min, in order to remove any contaminants. Fiber blanks analyses were performed using the same SPME and chromatographic conditions. Samples were prepared in duplicate. 

#### 2.2.4. GC-MS Analysis of Volatile Compounds of Cereal Flours and *broas*

The extracted volatiles were injected in the GC column in split mode (ratio 1:2). The oven temperature was kept at 35 °C for 5 min, followed by an increase of 5 °C min^−1^ to a final temperature of 230 °C, which was kept for 5 min (total run time of 48 min). The column carrier gas was helium at a constant flow rate of 2.1 mL min^−1^. The mass spectrometer was operated in the electron impact mode (EI) at 70 eV, scanning from *m*/*z* 30 to 300, at a scan rate of 555 scan s^−1^, and the ion source temperature was set at 250 °C. Desorption time was 5 min at 260 °C (injector temperature). 

As previously reported [40], the identification of 11 of the most ubiquitous volatiles from maize flours was confirmed by the analysis of the respective commercial reference standards and by an additional analysis of maize flours and standards on a Sapiens-Wax.ms column (60 m × 0.2 mm × 0.25 μm) (Teknokroma, Barcelona, Spain). 

### 2.3. Characterization of the Phenolic Composition and Total Carotenoids

Phenolic compounds of maize flours and *broas* were extracted with 50% aqueous ethanol, and the major soluble phenolic compounds (ferulic acid, *p*-coumaric acid, diferuloyl putrescine, *p*-coumaroyl feruloyl putrescine, dicoumaroyl spermidine and *bis*-diferuloyl putrescine) were quantitated by HPLC-DAD (high-performance liquid chromatography coupled to a diode array detector), following the procedures previously described [38,41].

The total carotenoids content was spectrophotometrically measured at 450 nm according to the AACC method 14-60.01 (AACC International, 2012). Results were expressed in micrograms of lutein equivalent per gram of sample, as the main carotenoid found in maize, as previously described [42].

### 2.4. Sensory Analysis

A sensory evaluation (‘appearance’, ‘color’, ‘smell and odor,’ ‘taste and aroma’, ‘texture’ and ‘global appreciation’) of *broas* was conducted using a hedonic quantitative response scale (International Organization for Standardization, 2003) in a test room (International Organization for Standardization, 2007) with a consumer panel of 52 assessors. The test was performed using a numeric category scale from 1 (extremely unpleasant) to 8 (extremely pleasant) for all the attributes. Further details on this hedonic test have been described elsewhere [43].

### 2.5. Data Analysis

The software LabSolutions GCMS solution Release 2.53SU1 (Shimadzu, Kyoto, Japan) was used to analyze the data and to calculate the areas of the peak chromatograms. The identification of volatile compounds was performed by comparison of the mass spectra obtained for each compound with the mass spectra from the software library (NIST 27 and WILEY 229 and 147). The linear retention index (LRI) was calculated for each compound using the retention times of a homologous series of *n*-alkanes (C_8_-C_20_) injected under the same chromatographic conditions, and results were compared with data from the literature. A positive identification was considered when the experimental spectra shared at least an 80% similarity with spectra from the software libraries and the LRI deviation was less than 1%, when comparing to the LRI reported in the literature. 

The software SPSS^®^ version 21 was used to: (1) evaluate the differences among the results obtained during the optimization of the experimental conditions (ANOVA and Student’s *t* test), and to (2) measure the degree of association between *broas* volatile compounds and (2a) maize flours’ volatiles, (2b) maize flours and *broas’* phenolic compounds, (2c) maize flours and *broas’* total carotenoids content and (2d) scores obtained in *broas’* sensory analysis. A non-parametric test (Spearman correlation) was used to measure the degree of association between variables, since they did not meet the assumptions for a parametric analysis (normal distribution and homoscedasticity). 

In order to identify the main differences among the 12 maize and *broas*, and to study their interdependent relations, a hierarchical cluster analysis was performed using *R* software. Since this analysis requires a number of variables lower than the number of samples, it was necessary to decrease the number of variables. Therefore, after variables’ standardization, a PCA (principal component analysis) was performed, in order to group the variables according to their correlations. The number of principal components considered was based on the Kaiser criteria and a cluster analysis was then performed. The distance was measured by the Euclidean distance, and Ward’s method was used to form the clusters, allowing the formation of clusters with the lowest relative standard deviations among the groups. The optimal number of clusters was estimated using two different methods, namely the elbow point and silhouette methods. A statistical significance test (*v.test*) was performed in order to identify the variables which significantly contributed to clusters’ differentiation.

## 3. Results and Discussion

### 3.1. HS-SPME-GC-MS Optimization

The analysis of volatile components was performed by headspace solid-phase microextraction (HS-SPME) followed by gas chromatography coupled to mass spectrometry (GC-MS). The commercial maize flour was used to optimize the conditions of analysis in order to achieve a higher extraction efficiency of the volatiles. The total chromatogram areas were measured, focusing on the aldehydes’ content, since they have been described as mainly responsible for the aroma of cereal flours [44]. Different types of SPME fibers were tested and a DVB/CAR/PDMS fiber coating was selected, as higher peak areas were obtained (data not shown). These results are in accordance with previous studies on cereals’ volatile compounds [8,44]. Different ratios between water and flour were tested, and the highest improvement in SPME efficiency was observed when 5 mL of water was added to 2 g of flour. Different sample temperatures were tested, and the best results were obtained at 70 °C. However, the variation among the triplicates was higher (Supplementary Figure S1), probably due to changes in the sample composition, namely to the increase in lipid oxidation and Maillard reactions [44]. Thus, the temperature of 60 °C was chosen for further analyses. Different extraction times were evaluated, and results show an increase in peak areas with higher exposure times (Supplementary Figure S2), possibly due to the increase of the activity of endogenous lipoxygenase after the addition of water [16]; however, these differences were not significant (*p* > 0.5). Taking into account the time required for exposure of the fiber and chromatographic analysis, a time of exposure of 40 min was selected. The repeatability of the whole procedure (*n* = 6) was determined, and the relative standard deviation (RSD) was 17%, similar to values previously reported [44]. The stability of the maize flours in the autosampler was analyzed for 32 h. After 20 h, compounds such as dodecanol, hexadecanoic acid and ethyl caprylate were detected in the chromatograms and tended to increase, probably due to the metabolism of bacteria and fungi [45,46,47,48]. Therefore, samples remained for a maximum of 18 h in the autosampler, in order to avoid significant changes in their volatile composition.

### 3.2. Identification of Volatile Compounds in Maize Flour and Broas 

**Figure 1:**
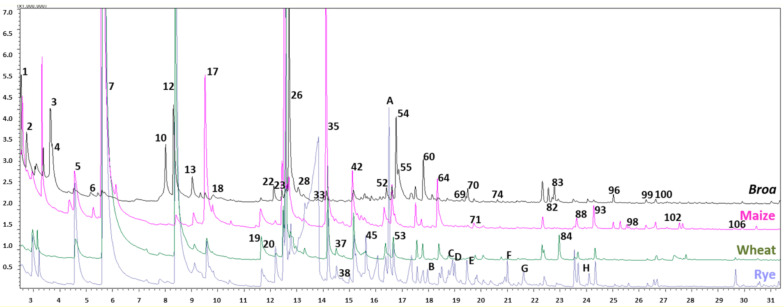
Chromatographic profiles of *broa* and maize, wheat and rye flours. Peaks are numbered according to Table 2 and letters represent compounds detected exclusively in rye and/or wheat flours, namely, A: α-terpinolene, B: camphor, C: octanoic acid, D: 4-terpineol, E: α-terpineol, F: linalyl acetate, G: nonanoic acid, H: 2(3H)-furanone-5-heptyldihydro.

All twelve maize flours and corresponding *broas* were analyzed using the optimized conditions. Illustrative chromatographic profiles of a maize flour (F1), corresponding *broa* (B1) and wheat and rye flours are presented in Figure 1. Forty-four compounds were identified in maize flours and eighty-seven in *broas* by comparison of their mass spectra with those from the software library and LRI from the literature. Their characteristic flavors, LRI, similarity indexes and retention times (RTs) are described in Table 2. 

Aldehydes were the most abundant volatiles detected in maize flours, corresponding to 30% of the total chromatogram area, which is in accordance with results previously reported [35,40,42]. Alcohols (21%), hydrocarbons (21%) ketones (11%), terpenes (<1%) and lipid-derived furans (<1%) were also among the most important families of compounds. It was not possible to confirm the identification of seven compounds, but putative identifications were performed. Peak 49 presents a spectrum that is very similar to 6-nonenal (*Z*) and 5-undecene (Z), having identical similarity indexes. The presence of the ion at *m*/*z* 29, also present in other aldehydes, suggests that this compound is 6-nonenal. In turn, the mass spectrum of peak 50 suggests a structure corresponding to pantolactone (dihydro-3-hydroxy-4,4-dimethyl-2(3H)-furanone), with a molecular ion at *m*/*z* 130 and three major fragments at *m*/*z* 43, 57 and 71. However, it was not possible to confirm the identification of this compound, since the LRI values found in the literature were not similar (>1% deviation) to the LRI obtained in the present work. The chromatographic profiles suggested that several different compounds could be co-eluting. For peak 99, two compounds can be proposed: geranylacetone and nerylacetone, with close similarity indexes. This peak was tentatively identified as geranylacetone, since it has been widely described in several maize-based products [24,35,36].

The families of compounds identified in *broas* were mainly aldehydes (19% of total chromatogram area), alcohols (16%) and hydrocarbons (14%). Ketones (7%), furans (5%), pyrans (3%), lactones (3%), esters (3%), acids (3%) and pyrazines (1%) were also present. All these compounds have been described in other baked cereal products [11,20]. As in maize flour, it was not possible to confirm the identification of some compounds due to the absence of LRI bibliographic values for a DB-5 column, but tentative identifications were made. Peak 29 showed a mass spectrum with a base peak at *m*/*z* 88, and other fragments at *m*/*z* 43, 60, 70, 99 and 115. A structure corresponding to ethyl hexanoate, with a similarity index of 85%, was suggested. This compound has been reported in baked cereal products [11]. Peak 35 presented a spectrum similar to that of 3-ethyl-2-methyl-1,3-hexadiene, with a molecular ion at *m*/*z* 124, a base peak at *m*/*z* 67 and fragments at *m*/*z* 39, 41, 55, 95 and 109. This compound has been described in the crust of whole-meal wheat bread and other maize-based foods [14,56]. A tentative identification for peak 47 was methylpentylfuran, with a molecular ion at *m*/*z* 110 and a base peak at *m*/*z* 95; however, it was not possible to confirm this identification since no LRI value was described in the literature. For peak 51, two compounds were suggested: 3,5-octadien-2-one (*E,E*) and 1-octyn-3-ol, as they presented similar spectra. However, the compound 3,5-octadien-2-one was selected, due to the presence of the base peak at *m*/*z* 95 and the molecular ion at *m*/*z* 124. Compound 74 was tentatively identified as 2-hexyl-1-octanol, due to the high-spectrum similarity index (90%), and it has been described in other cereals, such as rice [57,58].

Since rye and wheat flours were also included in the recipe of *broa*, these samples were analyzed in the same conditions as maize flours. The chromatographic profiles corresponding to these flours are presented in Figure 1. Compounds such as acetoin (peak 2), hexanoic acid (27), 3-octen-2-one (37) and 2,4-decadienal (*E,E*) (84) were detected in *broas* and rye and wheat flours, but not in maize flours. Other compounds, such as 2(3H)-furanone-5-heptyldihydro (peak H) and linalyl acetate (peak F), were detected only in wheat and rye flours. 2,4-Decadienal (*E,E*) (84) was particularly abundant in the commercial wheat flour. Rye was distinguished by the presence of acids, such as octanoic (peak C) and nonanoic (G) acids, and terpenes, such as α-terpinolene (A), camphor (B), 4-terpineol (D) and α-terpineol (E).

### 3.3. Characterization of the Volatile Composition of Maize Flours

The majority of the compounds identified in maize flours were aldehydes (hexanal, heptanal, octanal, 2-octenal, nonanal, 2-nonenal, decanal), alcohols (1-pentanol, 1-hexanol, 1-heptanol, 1-octen-3-ol), hydrocarbons and 2-pentylfuran, which have been described in cereal flours as secondary products of lipid oxidation of unsaturated fatty acids [15,20,37,44]. Other abundantly detected volatile compounds were geranylacetone, ionone and 6-methyl-5-hepten-2-one, derived from carotenoids’ oxidation [40,59,60,61].

In order to evaluate the differences among maize flours and to study possible relations among the different volatiles, a cluster analysis was performed and the Spearman coefficients among the main volatiles were determined. Twenty-nine compounds, described in Supplementary Table S1, were considered for these analyses, since the remaining fifteen were only present in trace amounts in all maize samples and it was not possible to accurately measure their peak areas. A PCA was performed in order to reduce the number of variables, since it was higher (29) than the number of samples. Following the Kaiser criteria, four components were retained after PCA analysis, which explained 93% of the total variance. The optimal number of clusters obtained by the elbow point and silhouette methods was two, one corresponding to all the traditional maize varieties (F1 to F11) and the other to the commercial maize sample (F12).

**Figure 2:**
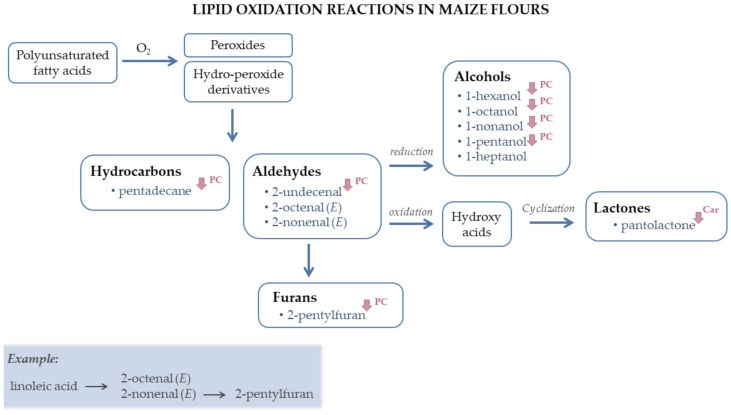
Representative scheme of lipid oxidation reactions [15,17,20,28,35] occurring in maize flour samples.

The commercial sample was distinguished from the traditional varieties due to the presence of benzaldehyde and higher amounts (*p* < 0.05) of other aldehydes (2-octenal (*E*), 2-nonenal (*E*), hexanal, heptanal, octanal, 2-heptenal (*Z*), 2-undecenal, decanal and 6-nonenal), alcohols (1-hexanol, 1-heptanol, 1-octen-3-ol, 1-octanol, 1-nonanol and 1-pentanol) and 3-ethyl-2-methyl-1,3-hexadiene, 2-pentylfuran, limonene, trimethyldodecane, 2-heptanone and pantolactone. The mentioned aldehydes showed very strong and positive correlations among them (Supplementary Table S2). Since these volatiles are mainly derived from the oxidation of polyunsaturated fatty acids (Figure 2) [44,62], these results suggest that more lipid oxidation reactions were occurring in the commercial flour. Very strong and positive correlations were also found between these aldehydes and 3-ethyl-2-methyl-1,3-hexadiene (Supplementary Table S2). Although the origin of this compound is not known, these high correlations suggest that it may also be a product of lipid oxidation. In fact, a recent study has shown that it was formed after black rice storage (>3 months) [63], and it has been recently described in purple sweet maize [64] and maize milk [65]. The highest concentration in lipid oxidation derivatives observed in F12 can be explained by its different genetic origin. In particular, the concentration of 2-octenal (*E*) and 2-nonenal (*E*), two by-products of linoleic acid oxidation, one of the most abundant lipids in maize kernel [66], was recently associated with a gene that codes for a linoleate 9S-lipoxygenase, an enzyme involved in linoleic acid metabolism [40]. Therefore, a higher enzymatic activity in F12 can explain its higher levels in 2-octenal (*E*) and 2-nonenal (*E*). Additionally, F12 was acquired already milled, and it may have been kept at room temperatures for a longer period than the traditional maize flours, contributing to its higher amounts in lipid oxidation products [67,68]. Another possibility for these differences could be the different granulometry of the commercial sample, since it presented a higher mean diameter and a large particle distribution range compared to traditional maize flours [43]. However, research has shown the opposite: finely milled cereal flours tend to be more susceptible to lipid oxidation reactions than flours with higher particle sizes [67,68], possibly due to the higher surface area that favors the contact with oxygen [67]. Thus, the different granulometry of the commercial flour was probably not the main reason for its higher content in aldehydes and alcohols. Ultimately, the lower content of lipid oxidation derivatives in the traditional maize flours may also be caused by their higher amounts in antioxidant compounds [11]. Consequently, the total carotenoids content and major soluble phenolic compounds present in maize flours (ferulic and *p*-coumaric acids, diferuloyl putrescine, coumaroyl feruloyl putrescine, dicoumaroyl spermidine and *bis*-diferuloyl putrescine) were quantitated (Supplementary Table S3) and correlated to their volatile composition (Supplementary Table S4). Results showed strong negative correlations among several volatile compounds derived from lipid oxidation [15,17], namely 1-hexanol, 2-pentylfuran, 1-octanol, 1-nonanol, 2-undecenal and pentadecane, and some phenolic compounds, such as ferulic acid, diferuloyl putrescine, coumaroyl feruloyl putrescine and *bis*-diferuloyl putrescine, whereas no positive correlations were found. Similarly, pantolactone, also possibly derived from lipid oxidation reactions [15], showed very strong and negative correlations (R < –0.70, *p* < 0.01) not only with diferuloyl putrescine, but also with total carotenoids’ content. Hence, phenolic compounds in traditional maize flours and, to a lesser extent, carotenoids, may have inhibited lipid oxidation reactions, and contributed to their longer preservation. In fact, ferulic acid has been approved in certain countries as a food additive to prevent lipid oxidation [69].

**Figure 3:**
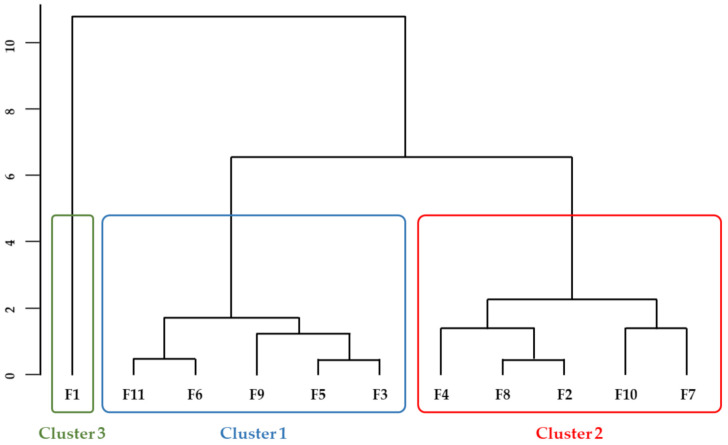
Dendrogram of cluster analysis of traditional maize flours.

As the differences between the traditional and commercial maize flours were high, it was not possible to detect any dissimilarities among the traditional maize varieties. Thus, a second analysis was performed, excluding the commercial sample and benzaldehyde, once it was not detected in the traditional maize varieties. The analysis was performed considering the first 5 components obtained by the PCA, which explained 91.3% of the total variance. The optimal number of clusters obtained by the elbow point and silhouette methods now corresponded to 3 clusters. The resulting dendrogram is presented in Figure 3 and the compounds that significantly contributed to discriminate the clusters are presented in Table 3. Samples from cluster 1 (F3, F5, F6, F9 and F11) were distinguished from cluster 2 (F2, F4, F7, F8, F10) by their lower contents in ketones (α-ionone, 6-methyl-5-hepten-2-one and geranylacetone), derived from carotenoids’ oxidation [40,59] and higher pantolactone contents (Supplementary Figure S3). Geranylacetone, α-ionone and 6-methyl-5-hepten-2-one showed strong and positive correlations among them (R > 0.7, *p* < 0.05) (Supplementary Table S2). Therefore, a higher content of carotenoids and/or higher carotenoids’ oxidation reactions led to an increase in these compounds. Indeed, strong and positive correlations were found between total carotenoids’ content and both α-ionone and geranylacetone (Supplementary Table S4). In addition, all the samples from cluster 2 were yellow kernels, showing higher total carotenoids’ content (Supplementary Table S3), corroborating the results from a previous study [40]. The concentrations in geranylacetone and α-ionone can also be influenced by the expression of genes encoding carotenoid cleavage dioxygenases [60,61]. Ultimately, higher amounts in compounds that inhibit carotenoids’ oxidation, such as ferulic and *p*-coumaric acid or other phenolic derivatives, can also contribute to lower concentrations in these compounds. In fact, moderate negative correlations were found between geranylacetone and *p*-coumaric acid and between 6-methyl-5-hepten-2-one and ferulic acid (Supplementary Table S4).

Finally, cluster 3, constituted by the sample F1, was distinguished by higher contents in compounds derived from lipid oxidation, similar to the commercial hybrid (F12). This sample was a synthetic open pollinated maize variety developed as an experimental higher-quality cultivar with increased precocity, obtained through the crossing of 12 maize populations (10 Portuguese traditional varieties and 2 American populations). As previously discussed for the commercial sample, the differences observed may be at least partially explained by their different genetic origin.

### 3.4. Characterization of the Volatile Composition of Broas

**Figure 4:**
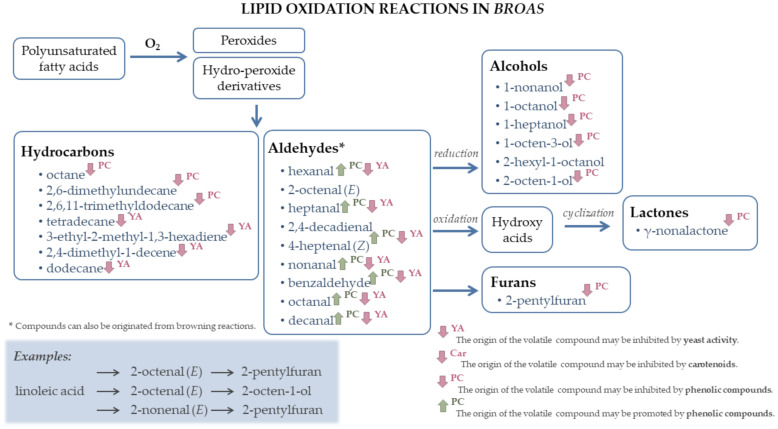
Representative scheme of lipid oxidation reactions [15,17,20,28,35] in *broas*.

The volatile compounds present in *broas* were mainly derived from lipid oxidation (Figure 4) and originated during bread making (fermentation (Supplementary Figure S4) and non-enzymatic browning reactions (Supplementary Figure S5). It is known that the increase of lipid oxidation products in breads is associated with (1) higher fermentation temperatures [15], (2) the use of wholegrain flours, such as in broas preparation, probably due to the additional lipid material in cereal germ [28], and (3) the presence of oxidative yeasts naturally present in sourdoughs, such as *Candida* spp. [16]. 4-Heptenal (*Z*) and 2,4-decadienal (*E,E*) were detected in *broas*, but not in maize flours. Both aldehydes have been reported as primary odorants to contribute to the flavor of wheat bread [56], and 2,4-decadienal has also been described as an important popcorn volatile [24]. They have also been described as lipid oxidation products and could have been formed during fermentation, due to the metabolic activity of yeasts and lactic acid bacteria [6]. Since 2,4-decadienal was particularly abundant in the commercial wheat flour used for *broas* preparation, it could also have been derived from the lipid oxidation of linoleic acid [28] present in this flour.

Benzaldehyde was detected in all *broas* despite being only detected in the commercial maize flour. It may have been produced by auto-oxidation of 2,4-decadienal [18] or formed from phenylalanine through the Ehrlich pathway and/or Strecker degradation (Supplementary Figure S4) [17,25]. Other compounds were also detected in *broas*, but not in maize flours, as acids (acetic and hexanoic acids), esters (ethyl hexanoate, ethyl octanoate, 1-hexyl acetate), ketones (octen-2-one, 3-hydroxy-2-butanone, 2-methyl-3-octanone and 3,5-octadien-2-one) and furanones (γ-*N*-caprolactone and γ-nonalactone). These compounds may have been originated by yeast and/or bacteria during fermentation [15,17,20,22]. Acetic acid is a well-known main product of fermentation by LAB and its concentration is higher when sourdough is used in breads; formulation [20], such as in the preparation of *broa*. In spite of being volatilized during baking [11], acetic acid may be a product of Maillard or caramelization reactions [17,20]. Furanones can also be products of carbohydrate dehydration and fragmentation that occurs during baking [21,25]. Other compounds may have been formed during the early steps of the Maillard reaction (sugar dehydration or fragmentation), such as furans, pyranones and furfurals [5,19,25,71], and in the last stages of the reaction, such as pyrroles and pyrazines (Supplementary Figure S4) [19]. *p*-Vinylguaiacol was also detected in *broas*, but not in maize flours. It has been described as an important odorant in other breads and baked products, derived from ferulic acid decarboxylation [8,28,29]. On the contrary, some compounds were lost after processing, such as aldehydes, alcohols and ketones, which may have been volatilized during baking [16,20], or participated in further reactions during bread making [35].

The contribution of a compound to the final bread aroma depends not only on its concentration, but also on its odorant power, which is determined by its odor threshold, or odor activity value (OAV) [11]. Therefore, some compounds, although present in low concentrations, may be more flavor-active than others present in higher concentrations [11]. The most important and potent volatile compounds found in *broas* and already described in wheat bread and other maize-based foods are highlighted in Table 2. Some of these compounds are positively associated with the pleasant aroma of breads, while others can be considered as off-flavors [15,20].

*Broas*’ crumb and crust samples were analyzed separately in the same conditions. The crust was characterized by a higher content in aldehydes, furans, pyrrolines and acids, while the crumb presented a higher content of alcohols and esters (data not shown). These results were expected, since different compounds are mainly present in the crumb, where temperatures during bread making are in general less than 100 °C, and others are formed in the crust, at temperatures of around 230–250 °C [5,11]. The main volatiles present in the crumb were compounds associated with lipid oxidation [35] and fermentation [11,12,16,72] and, in the crust, the number of volatile compounds resulting from the Maillard reaction [35] increased. Since the main objective of this work was to determine the volatile compounds of the whole bread, which may influence consumers’ choice, the results discussed in the present work did not go further into the differences between the crust and crumb.

**Figure 5:**
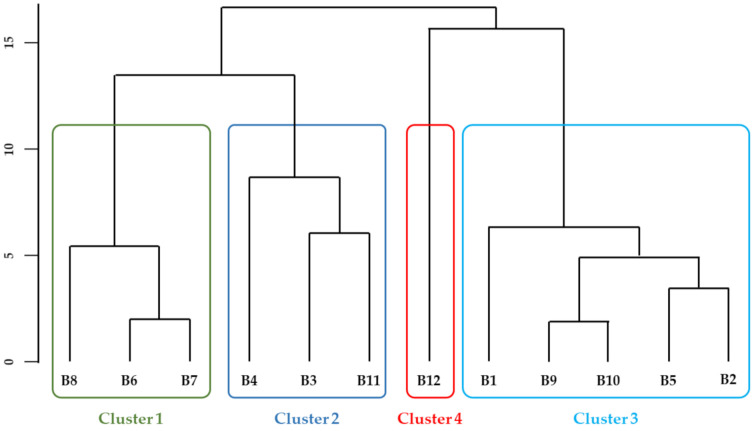
Dendrogram of cluster analysis of *broas*.

As for maize flours, the associations among different samples and the correlations among volatiles were studied. All eighty-seven compounds were considered for these analyses (Supplementary Table S5). After sample dimension reduction by PCA and based on the Kaiser criteria, 11 components were retained, which explained 100% of the total variance. Cluster analysis was then performed. The optimal number of clusters obtained by the elbow point method was in this case of four clusters, while by the silhouette method was five clusters. However, since the average silhouette width obtained for five clusters was very similar to that obtained for four clusters, the analysis was performed considering an optimal number of four clusters, and the resulting dendrogram is presented in Figure 5. The compounds that significantly contributed to discriminate the clusters are presented in Table 4.

*Broas* were differentiated mainly by their content in volatiles from lipid oxidation and formed during baking. In particular, clusters 3 and 4 were characterized by higher amounts in volatiles from lipid oxidation. As observed for maize flours, the *broa* prepared from the commercial maize variety (B12) was discriminated from all the others, belonging to cluster 4 (Figure 5 and Table 4). This sample was characterized by higher amounts in some aldehydes, alcohols and alkanes, suggesting that more lipid oxidation reactions [20,21] had occurred, probably due to the different genetic origin of the corresponding maize flour (F12), as previously discussed. It was also characterized by lower contents in methylpentylfuran. Although it was not possible to confirm the identification of methylpentylfuran, it is possible that it had been originated by lipid oxidation, such as 2-pentylfuran [15,17], and/or during the Maillard reaction, such as other furans [19]. On the contrary, cluster 3 (B1, B2, B5, B9 and B10) was characterized by higher contents in methylpentylfuran and, similar to cluster 4, by higher amounts in several low-odor threshold aldehydes derived from lipid oxidation. Cluster 2 (B3, B4 and B11) was characterized by lower amounts in volatiles from lipid oxidation and/or fermentation reactions [15,21,28], namely 3-ethyl-2-methyl-1,3-hexadiene, 3-octen-2-one, octanal and benzaldehyde. The lower amounts in 2,4-decadienal observed in these samples can explain their lower benzaldehyde contents, since benzaldehyde can be formed by the auto-oxidation of 2,4-decadienal (*E,E*) [18]. Finally, *broas* B6, B7 and B8 belonged to cluster 1, characterized by higher contents in compounds resulting from the baking process, particularly maltol, 1-furfurylpyrrole, 2-acetylpyrrole and 5-methylfurfural.

The correlations among *broas*’ volatiles were also evaluated (Supplementary Table S6). Similar to maize flours, strong positive correlations were found among lipid oxidation products, such as aldehydes, alcohols and some ketones [20]. Positive correlations (R > 0.59, *p* < 0.05) were also detected among compounds formed during baking, such as methylpyrazine, 5-methylfurfural, furfurylpyrrole and 2-furanmethanol, and among volatiles which may have been originated during fermentation, namely hexanoic acid, benzyl alcohol, ethyl hexanoate, 1-hexyl acetate, ethyl octanoate, γ-nonalactone, 3-octen-2-one and 2-methyl-3-octanone. Positive correlations were also commonly found among these volatiles and several alcohols, such as isopentanol, 1-pentanol, 1-hexanol and 1-heptanol, suggesting that these compounds have also been originated by bacteria or yeast fermentation. Conversely, significant negative correlations were found among some volatiles derived from fermentation, such as 2-methyl-1-butanol, phenylethyl alcohol and 2-methyl-3-octanone, with several lipid oxidation products, such as aldehydes (hexanal, 4-heptenal (*Z*), heptanal, benzaldehyde, nonanal and decanal) and hydrocarbons (3-ethyl-2-methyl-1,3-hexadiene, 2,4-dimethyl-1-decene, dodecane and tetradecane). Other authors have reported similar negative correlations in other food products [73]. One explanation for these correlations is the lower amylase and yeast activities in some flours, which consequently provided a higher amount of active oxygen for oxidation, contributing to the increase in lipid oxidation reactions [30]. Alternatively, a higher yeast activity may have promoted the metabolization of aldehydes to the corresponding alcohols. Moskowitz et al. [28] have reported that *p*-vinylguaiacol can significantly reduce the generation of Maillard-type aroma compounds, but only a modest negative correlation was found between this compound and methylpyrazine in *broas* (R = –0.587, *p* < 0.05), whereas no correlations were found between this compound and other Maillard derivatives (Supplementary Table S6).

The volatile composition of maize kernels plays a major role in overall end-product quality [40]. Therefore, in order to understand the influence of maize volatiles on *broas’* volatile composition, the volatile compounds detected in maize flours and *broas* were correlated (Supplementary Table S7). In general, higher amounts of lipid oxidation products were found in *broas* prepared from maize flours that presented higher contents in these compounds. For instance, 1-octen-3-ol was detected in higher concentrations in *broas* produced from maize flours with higher contents in 1-octanol (R = 0.804, *p* < 0.01) and maize flours with higher contents in heptanal originated *broas* with higher contents in 3-ethyl-2-methyl-1,3-hexadiene (R = 0.818, *p* < 0.01).

#### 3.4.1. Contribution of Phenolic Compounds for *Broas’* Volatile Composition

Some phenolic compounds, particularly free phenolic acids present in the outer layers of cereal grains, such as ferulic acid, may directly contribute to breads’ flavor [74], increasing their bitterness and astringency [30]. However, previous studies have demonstrated that the contribution of phenolic compounds to breads’ flavor is mainly due to their influence on their volatile composition [28,29,30]. Although most of maize phenolic compounds are insoluble and linked to arabinoxylans [32], it is believed that only the soluble phenolics are able to influence the volatile composition of breads [28,75]. Therefore, the main soluble phenolics of maize flours and *broas* were quantitated (Supplementary Table S3) and correlated with *broas’* volatile composition (Supplementary Table S8). Some examples of the influence of phenolic compounds on *broas* volatiles are represented in Figure 4, and Supplementary Figures S4 and S5.

Significant negative correlations were found between phenolic compounds from maize flours and *broas* and several volatiles derived from lipid oxidation reactions. In particular, strong negative correlations (R < –0.7, *p* < 0.05) were found between *broas’* diferuloyl putrescine and coumaroyl feruloyl putrescine contents and alkanes (octane, 2,6-dimethylundecane and 2,6,11-trimethyldodecane) and alcohols (1-octen-3-ol, 1-heptanol, 1-octanol). Negative correlations were also obtained between phenolic compounds and 1-nonanol and 1-octanol. Therefore, phenolic compounds seamed to inhibit lipid oxidation reactions not only in maize flours, as previously discussed, but also in *broas* and possibly during bread making, as described for other breads [30,33,73,76]. However, positive correlations (R > 0.57, *p* < 0.05) were found between both free ferulic acid and coumaroyl feruloyl putrescine in maize flours and several lipid oxidation aldehydes [15,20,44] in *broas*, such as hexanal, 4-heptenal, heptanal, nonanal, decanal and benzaldehyde. Some studies have reported that phenolics, including ferulic acid, can actually increase the generation of lipid oxidation volatiles [30], possible because they begin to show pro-oxidant behavior at higher concentrations [30,73]. Conversely, Moskowitz et al. [28] have demonstrated that the addition of ferulic acid to refined breads did not influence the generation of lipid oxidation products. The effectiveness of an antioxidant can be influenced by its location in the food matrix, survival during food processing, interactions with other food components [31] and by different hydrophilic properties of some antioxidants [30,73,77]. However, the mentioned aldehydes could have also been formed during baking [11] and not exclusively from lipid oxidation reactions.

Negative correlations were also found between phenolic compounds and some volatiles originated by yeast and/or bacteria during fermentation, such as ethyl hexanoate, ethyl octanoate, 1-hexyl acetate, benzyl alcohol, 2-methyl-1-butanol and γ-nonalactone [15,17,20,22], suggesting that hydroxycinnamic acids and hydroxycinnamic acid amides inhibited amylases and yeast activity, as it has already been described for ferulic acid [30]. These findings can also explain the positive correlations among aldehydes and phenolic compounds and the negative correlations among alcohols and phenolic compounds, since aldehydes can be reduced to alcohols by the activity of LAB and yeasts [20]. Thus, if these reduction reactions were inhibited by phenolic compounds, the levels of alcohols would be lower, whereas an increase in aldehydes’ content would be expected.

On the contrary, significant positive correlations were found among phenolic compounds and furan derivatives from browning reactions (Maillard and caramelization reactions, Supplementary Figure S4), namely, furfural, furfuryl alcohol, 2-acetylfuran and 5-methylfurfural. These findings are in accordance with previous studies, which have demonstrated an increase in furfural and 5-methylfurfural by feruloylated oligosaccharides [29], possibly due to their pro-oxidant effect at high concentrations, therefore increasing browning reactions [78]. The positive correlations of some aldehydes with phenolic compounds can also be explained by the increase in these reactions, since aldehydes can also be originated during baking [11]. Conversely, other authors have described a reduction in the content of Maillard volatiles in breads, such as furanones and other furan derivatives, by phenolic compounds, including ferulic acid [28,30,77], possibly due to the formation of adducts with dicarbonyls or scavenging reactions with radical precursors [28,29,30,73,77]. In fact, as previously discussed, the contents of γ-nonalactone (a furanone) were negatively correlated to some phenolics. However, taking into account the cluster analysis, γ-nonalactone was most likely originated during fermentation and not during baking.

Thus, these results suggest that phenolic compounds may: (1) act as antioxidants, inhibiting lipid oxidation reactions in both maize flours (Figure 2) and *broas* (Figure 4), (2) inhibit amylases and yeast fermentation (Supplementary Figure S4) and (3) act as pro-oxidants during baking, increasing the levels of Maillard and caramelization volatiles (Supplementary Figure S5). As previously reported [41], some insoluble phenolic compounds may become soluble after maize processing to *broas*, increasing their content in soluble phenolic compounds. Therefore, the increasing levels of phenolic compounds during bread making may have contributed to their action as pro-oxidants, thus increasing the level of Maillard derivatives.

#### 3.4.2. Contribution of Carotenoids for *Broas’* Volatile Composition

*Broas* produced from maize flours with a higher content in carotenoids showed higher amounts of geranylacetone and α-ionone (R > 0.85, *p* < 0.01) (Supplementary Table S8). Thus, maize flours with higher amounts of volatiles from carotenoids’ oxidation generated *broas* with higher amounts of carotenoids’ oxidation volatiles (Supplementary Table S7). For instance, although 6-methyl-5-hepten-2-one was not detected in *broas*, *broas* prepared from maize flours with higher contents in this compound showed higher geranylacetone contents (R = 0.832, *p* < 0.01). Both of these compounds can result from the degradation of phytoene and ζ-carotene [7,61].

Significant negative correlations were found between carotenoids’ content and some compounds formed during bread making (fermentation and/or baking), namely γ-*N*-caprolactone, 2-phenylacetaldehyde, acetoin (3-hydroxy-2-butanone), hexanoic acid and indole [20,21,25], suggesting that carotenoids inhibited the formation of these compounds (Supplementary Figures S4 and S5).

A positive correlation was observed between γ-*N*-caprolactone present in *broas* and pantolactone in maize flours (Supplementary Table S7), which indicates that pantolactone might have participated in further reactions during bread making, originating γ-*N*-caprolactone. Carotenoids could have contributed to the increase in other compounds formed during bread making, such as 2,3-dihydrobenzofuran, 2-methoxy-2,3,3-trimethylbutane and acetic acid, since significant positive correlations were found between them and carotenoids’ content (Supplementary Figure S5).

#### 3.4.3. *Broas’* Sensory Analysis and Volatile Composition

The results obtained from a consumer sensory evaluation of *broas* [43] were retrieved and compared here with their volatile composition, in order to determine the volatile compounds that might contribute to *broas* sensory characteristics. The sensory analysis scores are presented in Supplementary Table S9 and the correlations among these scores and *broas’* volatile compounds are presented in Supplementary Table S10.

As previously reported [43], the sensory analysis revealed poor discrimination among the 11 samples prepared from traditional maize varieties. The *broa* prepared from the commercial maize flour (B12) showed the lowest mean scores for all the evaluated attributes (‘appearance’, ‘smell and odor’, ‘texture’, ‘taste and aroma’, ‘color’ and ‘global appreciation’). In addition, the majority of the negative comments were attributed to this sample, including ‘dry texture’, ‘weak typical flavor’, ‘wheat bread flavor’, ‘with a weak maize flavor’ and ‘no history’. As previously discussed, the commercial *broa* was distinguished from all the traditional *broas* (Figure 5 and Table 4), essentially due to its higher contents in volatiles derived from lipid oxidation reactions, such as 2-octenal, which was very strongly and negatively correlated (R = –0.746, *p* < 0.01) with ‘taste and aroma’ scores. This aldehyde contributes to fatty, nutty, waxy and green notes [20,51]. This sample was also characterized by higher contents in benzyl alcohol, an extremely aroma-active compound associated with pleasant notes [15,17,20]. However, benzyl alcohol has been described in rye breads as a major factor responsible for their intense and bread-like flavors [16], and thus can contribute to a more bread-like flavor in *broas*. Hence, it may contribute to aroma characteristics in *broas* that are not typical of this type of bread, giving rise to some of the negative comments described above. It can also be responsible for a bitter taste [79], which was also referred to as a negative characteristic of this particular *broa*.

The *broa* B8 scored higher for the majority of the sensorial attributes (‘color’, ‘smell and odor’, ‘texture’ and ‘global appreciation’) and was often described by the consumer panel as ‘the tastiest’ and ‘with a good maize flavor’. This sample belonged to cluster 1, together with B6 and B7, characterized by higher contents in compounds resulting from the baking process associated with positive notes [19,20,21,24], particularly maltol, 1-furfurylpyrrole and 2-acetylpyrrole. Interestingly, these compounds have not been reported as abundant in other breads [20], but have been described in popcorn [24,36], extruded maize products [14] and tortilla chips [80], suggesting that they can have a contribution to the characteristic maize-based foods, and particularly *broas’* sweet aroma. Significant positive correlations were found between ‘taste and aroma’ scores and pyranones content (maltol and 3-hydroxy-2,3-dihydromaltol). Despite the relatively high odor threshold of 9 mg/kg (water), maltol was very abundant in the B8 sample, which showed the highest maltol content of all *broas* (Supplementary Table S5). It has a caramel-like odor and enhances the sweet taste of food [21]. In contrast, B12 showed the lowest contents of maltol of all *broas*, which also supports its importance on *broas* ‘taste and aroma’. B1 showed the second lowest contents in maltol and had the lowest contents in 3-hydroxy-2,3-dihydromaltol of all *broas*. Some of the comments attributed to B12, such as ‘weak typical flavor’ and ‘weak aroma and flavor’ were also attributed to B1, which was the sample with the lowest ‘taste and aroma’ scores among all *broas* prepared from traditional maize flours. As previously discussed, F1 was differentiated from all the other traditional maize flours (Table 3 and Figure 3) due to its higher contents in volatiles from lipid oxidation, in particular 2-octenal (*E*). Similarly, B1 belonged to cluster 3, together with B2, B5, B9 and B10, characterized by higher amounts in lipid oxidation products, such as hexanal and heptanal, which were negatively correlated with ‘taste and aroma’ scores.

B11, followed by B3, showed the highest ‘taste and aroma’ scores. These samples and B4, which showed the fourth highest scores, all belonged to cluster 2 (Figure 5 and Table 4), characterized by lower contents in volatiles from lipid oxidation reactions, in particular octanal, which has a low odor threshold and is considered an off-flavor of foods [17,24]. Indeed, a significant negative correlation was found between ‘taste and aroma’ scores and octanal. This cluster was also characterized by lower contents of 3-ethyl-2-methyl-1,3-hexadiene and benzaldehyde, which also showed negative correlations with ‘taste and aroma’ scores, along with other aldehydes derived from lipid oxidation reactions, namely 2-nonenal (*E*) and 2-undecenal, frequently described as negative contributors to the aroma of breads [20].

These results explain some differences among *broas* prepared from the traditional and commercial hybrid maize varieties and sustain the previous knowledge of producers regarding the better-quality aspects of traditional maize varieties for *broas’* production [4]. In particular, according to the producers, the *broa* of traditional maize is softer, sweeter and can be preserved for longer periods than *broas* produced with hybrid varieties [81]. Indeed, higher contents in pyranones may contribute to traditional *broas* sweeter taste, and lower contents in aldehydes suggest that lower lipid oxidation reactions were occurring in traditional samples, which can not only explain their longer preservation, but also contribute to their more pleasant flavor.

Positive correlations were found between ‘smell and odor’ scores and 2,3-dihydrobenzofuran, which was more abundant in *broas* with higher carotenoids’ content, as previously discussed. To the best of our knowledge, 2,3-dihydrobenzofuran has not been described neither in other maize-based foods nor cereal breads and was not detected in maize flours, so it was originated during the bread making process, similar to other furan derivatives [14,15,16]. This compound has been described in wines and soy sauce as a product of the Maillard reaction [82,83] and is associated with musky notes [55].

Very strong and positive correlations were found between the main products of carotenoids’ degradation (α-ionone and geranylacetone) and color scores, which can be explained by the preference for *broas’* yellowish color by the panel of consumers. The ‘global appreciation’ of *broas* was greatly influenced not only by ‘flavor and aroma’, but also by ‘texture’, which also showed a very strong positive correlation between them (Supplementary Table S11). *Broas* with higher contents in some volatiles associated with off-aromas, such as octanal, 2-octenal and 2-nonenal, also showed lower scores for texture, whereas samples with higher pyranones content showed higher scores. These findings may be explained by the proteolysis reactions that occur during sourdough fermentation [9,84]. Briefly, proteolysis produces amino acids and other precursors of aroma compounds during baking, enhancing the formation of volatiles related to better bread flavor [9], while simultaneously improving the dough rheology and bread texture, resulting in a large reduction of elasticity and firmness of the dough [84].

Most of the volatiles commonly described as great contributors for other cereal breads and maize-based foods, such as 2-acetylpyrroline, 2-(methylimino)-3-butanone and 2,3-butanedione [8,85], were not detected in *broas*. Therefore, their absence can account for the different sensory characteristics of *broas* [81], when comparing to other cereal breads. In particular, 2-acetyl-1-pyrroline, a compound derived from the Maillard reaction, has been referred to as the main compound responsible for the characteristic flavor of wheat bread crust [24,26,36]. However, this compound is highly volatile, may be poorly released to the headspace during extraction, degrades rapidly after baking and can be oxidized to 2-acetylpyrrole, which was detected in *broas* [21]. Furthermore, phenolic acids, such as ferulic acid, could have inhibited its production [28]. 2-Acetyl-1-pyrroline has been described as mainly responsible for the differences in the overall odors of wheat and rye breads, where it is present in much lower concentrations [26]. Therefore, its absence in *broas* can also contribute to their characteristic aroma.

## 4. Conclusions

The main volatile compounds present in maize flours were aldehydes and alcohols derived from lipid oxidation reactions, which were present in higher amounts in the commercial maize variety. Ketones from carotenoids’ oxidation, such as α-ionone and geranylacetone, were present in higher amounts in yellow maize varieties. The presence of higher lipid and carotenoids’ oxidation volatiles in maize flours directly contributed to higher amounts of similar volatiles in *broas*. Other volatile compounds present in *broas* were esters, furans, furfurals and pyranones, derived from the bread making process (fermentation and baking). The differences in *broas’* volatile composition were mainly due to lipid oxidation and browning reactions, which were influenced by soluble phenolic compounds. In particular, ferulic and *p*-coumaric acids and hydroxycinnamic acid amides inhibited lipid oxidation and fermentation reactions and promoted browning reactions. *Broas* less appreciated by consumers, especially the *broa* obtained from the commercial flour, showed higher lipid oxidation compounds, such as 2-octenal, hexanal and heptanal, and lower contents in pyranones, such as maltol and 3-hydroxy-2,3-dihydromaltol, in addition to lower phenolic compounds. Results showed that maize flours with higher levels in phenolic compounds give raise to *broas* with better sensory characteristics, especially related to ‘taste and aroma’. In conclusion, the selection of maize varieties richer in health-promoting phenolic compounds originate *broas* more appreciated by the consumers. These findings are relevant not only for the industry, contributing to the selection of the best maize varieties for higher-quality bread making, but also for maize breeders, who can add these quality traits to routine breeding of maize for *broas’* production. These results sustain the previous knowledge of producers regarding the better-quality aspects of traditional maize varieties for *broas’* production, and can contribute to the valorization of these varieties, which should be preserved as a safeguard against an unpredictable future.

## Figures and Tables

**Table 1:** Maize flours and *broas* identification and description.

Flour	*Broa*	Variety	Kernel Color	Description	Origin
F1	B1	**SinPre**	white	Synthetic open pollinated variety, from the cross of 12 divergent original maize populations developed as an experimental higher-quality cultivar with increased precocity.	VASO participatory maize breeding program [4]
F2	B2	**Aljezudo**	yellow	Flint-type FAO 300. Hybrid open pollinated variety: from the cross made around the years 2000–2005 between two historical populations, Aljezur × Amiudo.	VASO participatory maize breeding program [4]
F3	B3	**Bastos**	white	Early flint open pollinated variety.	VASO participatory maize breeding program [4]
F4	B4	**Amiúdo**	yellow	Early flint-type FAO 200, open pollinated variety adapted to stress conditions (soils with low pH, water stress and aluminum toxicity)	VASO participatory maize breeding program [4]
F5	B5	**Broa-213**	yellow	Early intermediate type, traditional farmer open pollinated variety.	Collected from the farmer in the 2005 expedition to the Central Northern region of Portugal [1]
F6	B6	**Pigarro**	white	Flint-type FAO 300, open pollinated variety, with strong fasciation expression, used in the best soils for human consumption	VASO participatory maize breeding program [4]
F7	B7	**Algarro**	yellow	Early flint-type. Hybrid open pollinated variety: from the cross made around the years 2000–2005 between two historical populations, Aljezur × Pigarro	VASO participatory maize breeding program [4]
F8	B8	**Castro Verde**	yellow	Late flint-type FAO 600, open pollination variety, with big kernel row number and large ear size	VASO participatory maize breeding program [4]
F9	B9	**Verdeal de Aperrela**	white	Late flint-type FAO 600, open pollinated variety, used for bread making	VASO participatory maize breeding program [4]
F10	B10	**Fandango**	yellow	Synthetic open pollinated variety, dent-type FAO 600, big kernel row number and large ear size	VASO participatory maize breeding program [4]
F11	B11	**Broa-57**	white	Early flint-type traditional farmer open pollinated variety	Collected from the farmer in the 2005 expedition to the Central Northern region of Portugal [1]
F12	B12	**Commercial**	white	Nacional Type 175, wholegrain flour (from hybrid maize variety)	Obtained already milled from a bakery

**Table 2:** Identification of detected compounds in maize flours (F) and *broas* (B).

Peak	B/F	RT (min)	Compound	SI (%)	LRI	LRI (Literature)	Odor Description
**1**	B	2.56	Acetic acid *	94	-	600 (DB-5) [49]	Sour [20,49], acid, pungent [20]
**2**	B	3.22	**Acetoin (3-hydroxy-2-butanone)**	95	-	718 (DB-5) [49]	Butterscotch, buttery, yogurt, creamy [20]
**3**	B	3.74	**Isopentanol**	97	-	736 (DB-5) [49]	Balsamic, alcoholic [17,20], malty [20]
**4**	B	3.82	**2-Methyl-1-butanol**	90	-	755 (DB-5) [49]	Wine, onion [49], malty [20]
**5**	B	4.65	**1-Pentanol**	97	-	769 (DB-5MS) [50]	Fruity [20,49], balsamic, fusel-like, sweet [20]
	F			96	-		
**6**	B	5.56	**Octane**	97	809	800 (DB-5) [49]	Alkane [20,49]
	F			94	809		
**7**	B	5.70	Hexanal *	97	813	819 (DB-5) [51]	Green, grassy, tallowy [20,51], fruity, acorn-like, fishy, herbal, leafy [51]
	F			96	813		
**8**	B	6.87	Methylpyrazine *	97	840	828 (DB-5) [49]	Popcorn [49], roasted, burnt, sweet [20]
**9**	B	6.94	Furfural * (2-furancarboxaldehyde)	97	843	830 (DB-5) [51]	Almond, sweet [20,51], woody, fruity, flowery [51], bread-like, soil, burnt roasted, toasted [20]
**10**	B	8.29	**Furfuryl alcohol (2-furanmethanol)**	96	865	866 (HP-5) [51]	Caramel-like [25,51], weak, fermented, burnt sugar, creamy [51], sweet, fruity [25], burnt, warm oil, mild [20]
**11**	B	8.29	N.I.	96	865	n/a	n/a
**12**	B	8.44	**1-Hexanol**	96	879	869 (DB-5 MS) [50]	Flowery [20,49], resin, green [49], green grass, woody, mild, sweet [20]
	F			96	879		
**13**	B	9.11	**2-*n*-Butylfuran**	94	895	893 (DB-5) [50]	Green [20]
**14**	B	9.16	**2-Heptanone**	91	897	895 (DB-5) [49]	Soapy [20,49], fruity, cinnamon [20]
	F			96	897		
**15**	B	9.45	**Nonane**	87	904	900 (DB-5) [49]	Alkane [49], fusel-like [51]
**16**	B	9.54	4-Heptenal (*Z*) *	90	906	895 (DB-5) [51]	Biscuit-like, sweet [20,51], boiled potato, creamy [51], putrid [20]
**17**	F	9.59	Heptanal *	97	909	900 (DB-5) [51]	Citrus, fatty, rancid [20,51], green, dry fish, pesticide, solvent, smoky, fruity [51], malty [20]
	B			97	909		
**18**	B	9.90	**2-Acetylfuran [1-(2-Furanyl)ethanone]**	95	915	910 (DB-5) [51]	Sweet [25,51], balsamic-cinnamic note, cereal [51], caramel-like, fruity [25], smoky, roasted [20]
**19**	B	11.72	**2-Heptenal (*Z*)**	94	963	964 (DB-5) [51]	Sour, green, vegetable, fresh, fatty [10]
	F			94	963		
**20**	B	11.82	Benzaldehyde *	83	967	961 (DB-5) [51]	Almond, caramel [20]
	F			95	967		
**21**	B	11.87	5-Methylfurfural *	82	966	978 (DB-5) [49]	Almond [20,49], caramel, burnt sugar [49], sweet, bitter [20]
**22**	B	12.24	**1-Heptanol**	95	977	969 (DB-5) [50]	Green [20,52], woody, heavy, oily, fresh, light green, nutty [52]
	F			95	977		
**23**	F	12.51	1-Octen-3-ol *	94	985	980 (DB-5) [51]	Mushroom [20], garlic, spicy, rubbery, carrots, herbaceous, dirty, dust, earthy [51]
	B			95	985		
**24**	F	12.67	**6-Methyl-5-hepten-2-one**	85	988	985 (DB-5) [51]	Mushroom, earthy, vinyl, rubbery, woody, blackcurrant, boiled fruit [51]
**25**	B	12.74	**2-Methyl-3-octanone**	90	990	985 (DB-5 MS) [50]	n/f
**26**	F	12.81	2-Pentylfuran *	93	993	991 (BPx-5) or 992 (HP-5 MS) [51]	Buttery, green bean [20,51], floral, fruity [15,20], mushroom, raw nuts [20]
	B			94	993		
**27**	B	12.99	**Hexanoic acid**	88	997	1019 (DB-5)	Sweaty, cheesy, goat-like [20,51], pungent, rancid [51], fatty [20]
**28**	B	13.16	**Decane**	85	1001	1000 (DB-5) [49]	Alkane [49]
**29**	B	13.20	Ethyl hexanoate *	85	1003	1000 (DB-5) [50]	Apple peel, fruity [20]
**30**	B	13.35	Octanal *	91	1007	1004 (DB-5) [51]	Citrus, flowery [20,51], lemon, stew-like, boiled meat-like, rancid, soapy, green, fruity, orange [51]
	F			91	1007		
**31**	F	13.63	**2,4-Heptadienal (*E,E*)**	87	1017	1003 (DB-5) [51]	Orange oil, oily, fatty, rancid [51]
**32**	B	13.69	**1-Hexyl acetate**	90	1017	1008 (DB-5) [51]	Fruity, spicy, herbal [20,51], sweet wine, rubbery, tobacco, acidulous, citrus, green [51]
**33**	B	14.12	**Limonene**	87	1030	1031 (DB-5) [51]	Citrus [20,51], licorice, green, ethereal, fruity [51]
	F			93	1030		
**34**	B	14.12	N.I.	87	1030	n/a	n/a
**35**	F	14.20	**3-Ethyl-2-methyl-1,3-hexadiene**	89	1033	1030 (VF-5 MS) [50]	n/f
	B			89	1033		
**36**	B	14.52	Benzyl alcohol * (benzenemethanol)	81	1040	1039 (DB-5) [49]	Sweet, flowery [49], pleasant aromatic [20]
**37**	B	14.56	**3-Octen-2-one**	87	1043	1040 (DB-5) [49]	Nut, crushed bug [49], earthy type [20]
**38**	F	14.68	2-Phenylacetaldehyde *	85	1045	1043 (DB-5) [51]	Green [25,26], floral, hyacinths [25], metallic [26], honey-like, sweet [20]
	B			91	1047		
**39**	B	14.72	N.I. (3 compounds)	91	1047	n/a	n/a
**40**	F	14.77	**Isooctanol** [T.I.]	84	1049	1051 (DB-1) [51]	Fatty, orange, rose [51]
**41**	B	15.08	**ϒ** **-*N*-caprolactone** **[5-ethyldihydro-2(3H)-furanone]**	86	1058	1056 (HP-5MS) [51]	Coumarin-like, sweet [51]
**42**	B	15.22	2-Octenal (*E*) *	96	1062	1056 (DB-5) [51]	Fatty, nutty [20,51], burdock-like, sweet, sour, waxy, green, burnt, mushroom [51], roasted [20]
	F			96	1062		
**43**	B	15.49	**2-Acetylpyrrole**	91	1069	1060 (DB-5), 1072 (DB-5) [51]	Herbal, nutty, anisic, sweet [51], roasted, biscuits [20]
**44**	F	15.51	**2-Octen-1-ol (*E*)**	92	1069	1048 (DB-1) [49], 1064 (DB-5 Interpolated)	Green, vegetable-like [20]
	B			91	1071		
**45**	B	15.67	**1-Octanol**	91	1075	1072 (DB-5) [53]	Sharp, fatty, waxy, citrus [52], earthy, moldy vegetable [20]
	F			83	1075		
**46**	B	15.83	**2,4-Dimethyl-1-decene** [T.I.]	84	1080	n/f	n/f
**47**	B	15.94	**Methylpentylfuran** [T.I.]	87	1083	n/f	n/f
**48**	F	16.21	**2-Nonanone**	89	1091	1090 (DB-5) [49]	Hot milk, soap, green [49]
**49**	F	16.31	**6-Nonenal (*Z*)**	89	1094	1101 (DB-5) [51]	Cucumber, green, melon, waxy [52]
**50**	F	16.35	**Pantolactone [dihydro-3-hydroxy-4,4-dimethyl-2(3H)-furanone]** [T.I.]	90	1095	990 (HP-1) [51]	Cotton candy [49], licorice, smoky, toasted bread [54]
**51**	B	16.39	**3,5-Octadien-2-one (*E,E*)** [T.I.]	79	1096	1068 (DB-5) [51]	Fresh, sweet, woody, mushroom [51]
**52**	B	16.51	**Undecane**	94	1100	1099 (DB-5) [51]	Fusel-like [51]
**53**	F	16.70	Nonanal *	96	1107	1103 (DB-5), 1104 (DB-5), 1108 (DB-5) [51]	Citrus, soapy [20,51], gravy, green, tallowy, fruity, gas, chlorine, floral, waxy, sweet, melon, fatty, lavender [51]
	B			96	1107		
**54**	B	16.93	**Maltol** **(3-hydroxy-2-methyl-4H-pyran-4-one)**	95	1113	1108 (DB-5) [51]	Caramel-like [51], warm-fruity, caramelized/sweet [20]
**55**	B	16.98	Phenylethyl alcohol * **(2-phenylethanol)**	95	1115	1118 (DB-5) [49]	Rose, honey [20,49], spice, lilac [49], wilted rose [20]
**56**	B	16.98	N.I.	95	1115	n/a	n/a
**57**	B	17.32	**3,7-Dimethyldecane**	86	1126	1127 (HP-5 MS) [50]	n/f
**58**	B	17.42	**2,7-Dimethyl-1-octanol** [T.I.]	84	1129	1625 (DB-Wax) [50]	n/f
**59**	B	17.62	**2-Methoxy-2,3,3-trimethylbutane** [T.I.]	74	1136	n/f	n/f
**60**	B	17.94	**3-Hydroxy-2,3-dihydromaltol (2,3-dihydro-3,5-dihydroxy-6-methyl-4H-pyran-4-one)**	90	1144	1134 (DB-5) [51]	Caramelized [20]
**61**	B	17.94	N.I. (2 compounds)	90	1144	n/a	n/a
**62**	B	18.24	3-Nonen-1-ol (*Z*) *	94	1155	1134 (DB-1) [51], 1152 (DB-5 interpolated)	Sweet, green [51], waxy [20]
**63**	B	18.33	**1-Chloro-octadecane**	80	1159	1320 (Carbowax) [51]	Sweet, green [51], waxy [20]
**64**	B	18.45	2-Nonenal (*E*) *	96	1163	1162 (DB-5) [51]	Green, fatty, tallowy [20,26,51], cucumber-like [20,51], soapy, floral, sweet, wet, earthy, plastic [51], paper [20]
	F			96	1163		
**65**	B	18.45	N.I. (2 compounds)	96	1163	n/a	n/a
**66**	B	18.69	**3-Methylundecane**	93	1170	1169 (DB-5) [50]	n/f
**67**	B	18.82	**1-Nonanol**	84	1174	1174 (DB-5 MS) [51]	Citrus [20]
	F			91	1174		
**68**	B	18.97	**1-Furfurylpyrrole**	81	1179	1133 (DB-1) [51], 1166 (DB-5 interpolated)	Roasted, chocolate, green [51], vegetable [20]
**69**	B	19.53	Ethyl octanoate *	92	1198	1195 (DB-5) [51]	Sweet, soapy, fresh, fruity [20,51], fatty, floral, green leafy, menthol, anise, baked-fruity [51]
**70**	B	19.54	**Dodecane**	94	1200	1199 (DB-5) [51]	Fusel-like [51]
	F			94	1197		
**71**	F	19.80	Decanal *	96	1205	1209 (DB-5) [49]	Stewed, burnt, green, waxy, orange skin-like, floral, lemon, fatty, herbaceous, soapy [51], citrus [20]
	B			95	1208		
**72**	B	19.96	**2,6-Dimethylundecane**	91	1212	1210 (DB-5 MS) [50]	n/f
**73**	B	20.24	**2,3-Dihydrobenzofuran**	88	1222	1226 (DB-5) [50]	Musky notes [55]
**74**	B	20.73	**2-Hexyl-1-octanol** [T.I.]	90	1239	2162 (DB-Wax) [50]	n/f
**75**	B	20.80	N.I.	87	1242	n/a	n/a
**76**	B	20.93	N.I.	82	1246	n/a	n/a
**77**	B	21.47	N.I. (ketone)	84	1265	n/a	n/a
**78**	B	22.02	**2-Methyldodecane** [T.I.]		1284	n/a	n/a
**79**	B	22.35	**Indole (2,3-benzopyrrole)**	93	1295	1288 (DB-5) [51]	Sweet, burnt, floral, jasmine, earthy [51], animal [20]
**80**	B	22.43	N.I.	88	1299	n/a	n/a
**81**	B	22.66	N.I.	92	1307	n/a	n/a
**82**	B	22.80	***p*** -Vinylguaiacol * **(2-methoxy-4-vinylphenol)**	94	1312	1313 (DB-5) [51]	Clove [49,51], curry [49], phenolic, smokey [51]
**83**	B	22.89	N.I.	89	1316	n/a	n/a
**84**	B	23.02	2,4-Decadienal (*E,E*) *	91	1321	1319 (DB-5) [51]	Fatty [49,51,52], fried, wax [49,51], citrus [51,52], meaty, pungent, green [51]
**85**	B	23.36	N.I.	87	1333	n/a	n/a
**86**	B	23.49	N.I.	90	1338	n/a	n/a
**87**	B	23.60	N.I.	82	1342	n/a	n/a
**88**	F	23.68	N.I.	88	1345	n/a	n/a
**89**	B	23.73	N.I.	82	1347	n/a	n/a
**90**	B	23.83	**5-Methyltridecane**	95	1352	1355 (DB-5) [50]	n/f
	F			82	1349		
**91**	B	24.16	γ-Nonalactone * **[5-pentyl-dihydro-2(3H)-furanone]**	93	1363	1360 (DB-5MS) [50]	Coconut [20,26,49], peach [49], sweet, fruity [20]
**92**	B	24.25	**2-Undecenal**	87	1366	1365 (DB-5) [51]	Fruity [20,51], geranium, metallic, pungent, sweet, green, fatty [51]
	F			83	1363		
**93**	F	24.31	N.I.	87	1369	n/a	n/a
**94**	F	24.38	**2,6,11-Trimethyldodecane**	91	1371	1375 (DB-5) [50]	n/f
	B	24.39	**2,7,10-Trimethyldodecane**		1372		
**95**	B	24.93	**1-Tetradecene**	93	1392	1392 (DB-5) [50,51]	n/f
	F			94	1392		
**96**	F	25.10	**Tetradecane**	96	1397	1399 (DB-5) [51]	Mild herbaceous, sweet, fusel-like [51]
	B			97	1400		
**97**	F	25.34	**Dodecanal**	86	1408	1408 (DB-5) [51]	Oily, herbal, fatty, citrus, waxy [51]
**98**	B	25.74	**α-Ionone**		1423	1426 (DB-5 [51]	Floral, violet, woody, fruity [51]
	F				1423		
**99**	B	26,37	**Geranylacetone**	93	1448	1448 (DB-5) [51]	Fresh, floral, rosy-green, fruity odor [51]
	F			89	1448		
**100**	B	26,96	N.I.	91	1471	n/a	n/a
**101**	F	26,98	**Dodecanol**	90	1473	1470 (DB-5) [50]	Waxy-type [20]
**102**	F	27,65	**Pentadecane**	93	1499	1500 (DB-5) [51]	Mild green, fusel-like [51]
	B			92	1499		
**103**	F	28,79	N.I.	90	1547	n/a	n/a
**104**	B	28,88	**2-Butyl-1-octanol** [T.I.]	91	1550	1277 (DB-5) [50]	n/f
**105**	F	29,25	N.I.	90	1566	n/a	n/a
**106**	F	29,94	**Hexadecane**	95	1595	1600 (DB-5) [51]	Fusel-like, fruity, sweet [51]

RT: retention time; min: minutes; SI: similarity index; LRI: linear retention index; N.I.; not identified; T.I.: tentatively identified; n/a: not applicable; n/f: not found.  * Important volatile compounds described in wheat and rye breads, popcorn or extruded maize products [8,17,20,24,26,35].

**Table 3:** Maize flours’ volatile compounds that contributed to discriminate the different clusters.

Chemical Class	Compound	Suggested Origin
**Cluster 1**: F11, F6, F9, F5, F3		
Ketones	α-Ionone (−)	Degradation of δ-carotene [60,61]
	6-Methyl-5-hepten-2-one (−)	Degradation of phytoene, ζ-carotene, lycopene, δ-carotene [61]
	Geranylacetone (−)	Degradation of phytoene and ζ-carotene [7,61]
Alcohols	1-Nonanol (−)	Lipid oxidation [17]
	1-Hexanol (−)	
	1-Heptanol (−)	
	1-Octen-3-ol (−)	
	1-Pentanol (−)	
Aldehydes	Decanal (−)	Lipid oxidation [15]
	6-Nonenal (*Z*) (−)	
Hydrocarbons	Pentadecane (−)	Lipid oxidation [21]
Lactones	Pantolactone (+)	Lipid oxidation [15]
**Cluster 2**: F4, F8, F2, F10, F7		
Lactones	Pantolactone (−)	Lipid oxidation [15]
Ketones	α-Ionone (+)	Degradation of δ-carotene [7,61]
	6-Methyl-5-hepten-2-one (+)	Degradation of phytoene, ζ-carotene, lycopene, δ-carotene [61]
	Geranylacetone (+)	Degradation of phytoene and ζ-carotene [7,61]
Alcohols	1-Nonanol (+)	Lipid oxidation [17]
	1-Hexanol (+)	
**Cluster 3:** F1		
Aldehydes	2-Octenal (*E*) (+)	Lipid oxidation [15]
	Octanal (+)	
	Heptanal (+)	
	2-Nonenal (+)	
	Hexanal (+)	
	2-Undecenal (+)	
	2-Heptenal (Z) (+)	
	Decanal (+)	
	6-Nonenal (+)	
Terpenes	Limonene (+)	Plant metabolism and signaling [70]
Alcohols	1-Octanol	Lipid oxidation [17]
Hydrocarbons	Pentadecane	Lipid oxidation [21]
	3-Ethyl-2-methyl-1,3-hexadiene	
Furans	2-Pentylfuran	Lipid oxidation, from (*E*)-2-nonenal [15,17]

(+) Compounds present in higher concentrations (*p* < 0.05). (−) Compounds present in lower concentrations (*p* < 0.05).

**Table 4:** *Broas*’ volatile compounds that contributed to discriminate the different clusters.

Chemical Class	Compound	Suggested Origin
**Cluster 1:** B6, B7, B8		
Furans	2-Pentylfuran * (−)	Lipid oxidation, from (*E*)-2-nonenal [17]
Hydrocarbons	1-Chlorooctadecane (+)	Lipid oxidation [21]
	Undecane (+)	
	Decane (+)	
	3,7-Dimethyldecane (+)	
	3-Methylundecane (+)	
	2,4-Dimethyl-1-decene (+)	
Pyrroles	1-Furfurylpyrrole (+)	Last stages of the Maillard reaction [19]
	2-Acetylpyrrole (+)	Maillard reaction, from oxidation of 2-acetyl-1-pyrroline [21]
Alcohols	2-Hexyl-1-octanol (+)	Yeast fermentation or reduction of aldehydes from lipid oxidation [17]
Pyranones	Maltol (+)	Degradation of disaccharides, such as maltose or lactose [21]
Furfurals	5-Methylfurfural * (+)	Degradation of sugars [11,20]
**Cluster 2:** B3, B4, B11		
Ketones	3-Octen-2-one (−)	Lipid oxidation [20], fermentation [20] or sugar degradation [11]
Aldehydes	Octanal * (−)	Lipid oxidation [15]
	2,4-Decadienal (*E,E*) * (−)	Lipid oxidation of linoleic acid [28]
	Benzaldehyde * (−)	Ehrlich pathway or Strecker degradation, from phenylalanine [17,25], or auto-oxidation of 2,4-decadienal [18]
Hydrocarbons	3-Ethyl-2-methyl-1,3-hexadiene (−)	Lipid oxidation [21]
	Tetradecane (+)	
	1-Tetradecene (+)	
**Cluster 3:** B1, B2, B5, B9, B10		
Alcohols	2-Octen-1-ol (−)	Yeast fermentation or reduction of aldehydes from lipid oxidation [17]
Pyrroles	1-Furfurylpyrrole (−)	Last stages of the Maillard reaction [19]
Alkanes	Nonane (−)	Lipid oxidation [21]
	Dodecane (−)	
Aldehydes	4-Heptenal (*Z*) * (+)	Lipid oxidation [15,20,44]
	Heptanal * (+)	
	Hexanal * (+)	
	Decanal * (+)	
Furans	Methylpentylfuran (+)	n/f
**Cluster 4**: B12		
Furans	Methylpentylfuran (−)	n/f
Aldehydes	2-Phenylacetaldehyde * (+)	Strecker reaction [16] and Ehrlich pathway [16], from phenylalanine [17,20,25]
	2-Heptenal (*Z*) (+)	Lipid oxidation [15,20,44]
	2-Octenal * (*E*) (+)	
Alcohols	1-Octen-3-ol * (+)	Lipid oxidation [20]
	2-Octen-1-ol (+)	Yeast fermentation or reduction of aldehydes from lipid oxidation [17]
	Benzyl alcohol * (+)	
	1-Pentanol (+)	
Alkanes	2,7,10-Trimethyldodecane (+)	Lipid oxidation [21]
	5-Methyltridecane (+)	
	2,6-Dimethylundecane (+)	
Pyrroles	Indole (+)	Non-enzymatic browning reactions [12]
Terpenes	Limonene (+)	Plant metabolism and signaling [70] and yeast fermentation [16]
Pyrazines	Methylpyrazine * (+)	Last stages of the Maillard reaction [19]
Furanones	ϒ-Nonalactone * (+)	Lipid oxidation of oleic and linoleic acid [15], fermentation [22], Maillard reaction [20,21]

n/f: not found. (+) Compounds present in higher concentrations (*p* < 0.05). (−) Compounds present in lower concentrations (*p* < 0.05).  * Important volatile compounds described in wheat and rye breads, popcorn, extruded maize products or other cereal-based foods [8,17,20,24,26,35].

## Data Availability

The data supporting the findings of this study are available within the article and its supplementary material.

## Supplementary Materials

The following are available online at https://www.mdpi.com/article/10.3390/biom11101396/s1, Figure S1: Average of the total chromatogram areas at different temperatures, Figure S2: Average of the total chromatogram areas at different extraction times, Figure S3: Representative scheme of carotenoids’ oxidation reactions occurring in maize flour samples, Figure S4: Representative scheme of fermentation reactions in *broas*, Figure S5: Representative scheme of non-enzymatic browning reactions in *broas*. Table S1: Average peak areas obtained for each maize flour and considered for the cluster analysis, Table S2: Spearman correlation coefficients among maize flours’ volatile compounds, Table S3: Major soluble phenolic compounds and total carotenoids’ content of maize flours and *broas*, Table S4: Spearman correlation coefficients between maize flours’ volatile compounds and the content of major phenolics and total carotenoids, Table S5: Average peak areas obtained for each *broa* and considered for the cluster analysis, Table S6: Spearman correlation coefficients among *broas’* volatile compounds, Table S7: Spearman correlation coefficients among the volatile compounds from traditional maize flours and *broas*, Table S8: Spearman correlation coefficients between *broas’* volatile compounds and (1) the major phenolic compounds and (2) total carotenoids’ content of both *broas* and maize flours, Table S9: Average *broas’* sensorial analysis scores, Table S10: Spearman correlation coefficients between *broas’* volatile compounds and sensorial analysis scores, Table S11: Spearman correlation coefficients among *broas’* sensorial analysis scores.
