# Supplementary material for: Shedding Light on the Volatile Composition of Broa, a Traditional Portuguese Maize Bread
Source: Biomolecules. 2021 Sep 22;11(10):1396. doi: 10.3390/biom11101396 (PMC8533067; doi:10.3390/biom11101396)
Supplement: Supplementary file 1 [file biomolecules-11-01396-s001.zip › biomolecules-1365314-supplementary.pdf]

Shedding light on the volatile composition of *broa*, a traditional Portuguese maize bread

Andreia Bento-Silva, Noélia Duarte, Maria Belo, Elsa Mecha, Bruna Carbas, Carla Brites, Maria Carlota Vaz Patto and Maria Rosário Bronze

Supplementary Information

Content

1. HS-SPME-GC-MS optimization

Figure S1: Average of the total chromatogram areas at different temperatures………………………………………… ii

Figure S2: Average of the total chromatogram areas at different extraction times……………………………………… ii

2. Maize flours' volatile compounds

Table S1: Average peak areas obtained for each maize flour and considered for the cluster analysis………………………… iii

Table S2: Spearman correlation coefficients among maize flours' volatile compounds……………………………………… iv

Table S3: Major soluble phenolic compounds and total carotenoids content of maize flours and *broas*…………………… v

Table S4: Spearman correlation coefficients between maize flours' volatile compounds and the content in major phenolics and total carotenoids…………………………………………………………………………………… vi

Figure S3: Representative scheme of carotenoids oxidation reactions occurring in maize flour samples………………… vi

3. *Broas'* volatile compounds

Figure S4: Representative scheme of fermentation reactions in *broas*………………………………………………… vii

Figure S5: Representative scheme of non-enzymatic browning reactions in *broas*………………………………………… viii

Table S5: Average peak areas obtained for each *broa* and considered for the cluster analysis……………………………… ix

Table S6: Spearman correlation coefficients among *broas'* volatile compounds……………………………………………… xii

Table S7: Spearman correlation coefficients among the volatile compounds from traditional maize flours and *broas*….. xvii

3.1. Contribution of phenolic compounds and total carotenoids content for broas' *volatile composition*

Table S8: Spearman correlation coefficients between *broas'* volatile compounds and (1) the major phenolic compounds and (2) total carotenoids content of both *broas* and maize flours…………………………………………… xix

3.2. Broas' *sensory analysis and volatile composition*

Table S9: Average *broas'* sensorial analysis scores……………………………………………………………… xxii

Table S10: Spearman correlation coefficients between *broas'* volatile compounds and sensorial analysis scores………… xxii

Table S11: Spearman correlation coefficients among *broas'* sensorial analysis scores………………………………… xxiii

1. HS-SPME-GC-MS optimization

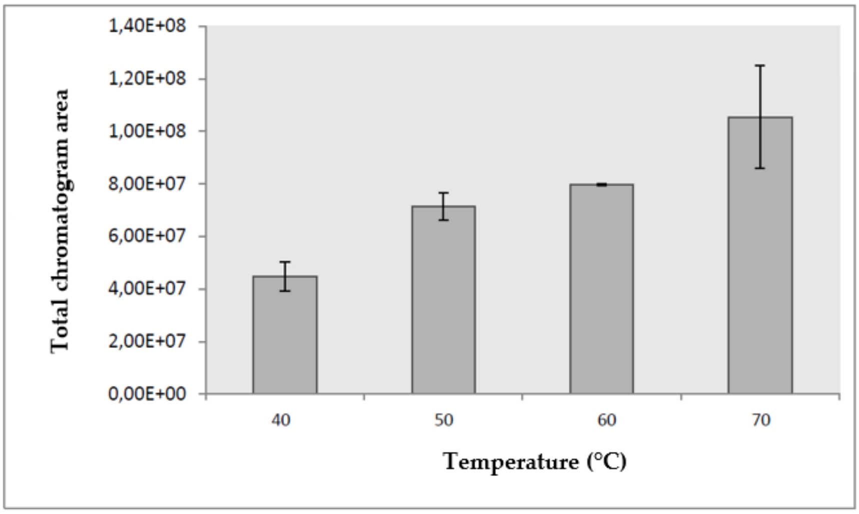

Figure S1: Average of the total chromatogram areas (n=3) at different temperatures.

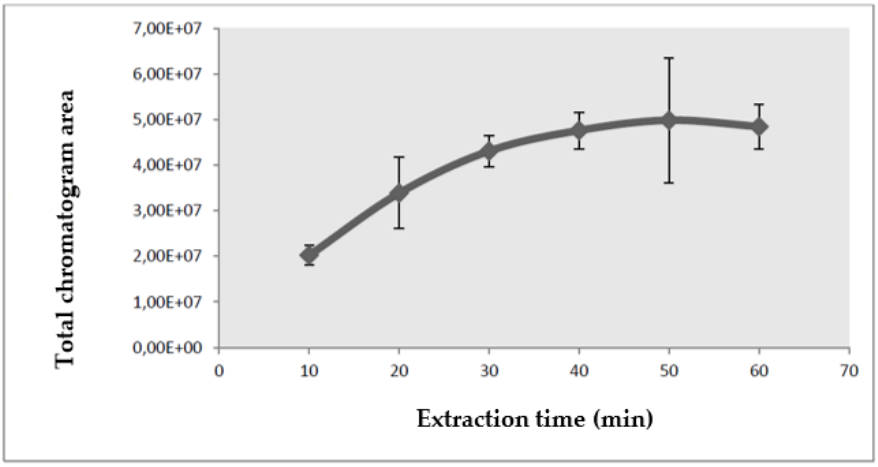

Figure S2: Average of the total chromatogram areas (n=3) at different extraction times.

2. Maize flours' volatile compounds

Table S1: Average peak areas obtained for each maize flour and considered for the cluster analysis.

Peak	F1	F2	F3	F4	F5	F6	F7	F8	F9	F10	F11	F12
**5**	1658377	1009855	1256498	1217227	1060382	1327411	1799584	1476005	919320	1838126	725925	2210163
**6**	94738	110760	117426	164492	99848	355394	296692	68028	55465	174295	308121	139354
**7**	16595178	8977672	5575694	4341115	6491413	6763045	9356316	7880485	4373802	8572044	2648452	81446621
**12**	781602	1130851	1117981	877255	446660	790109	1226260	1158610	534814	1022466	673474	12351643
**14**	244418	320968	294144	125473	264506	270256	306814	243268	91076	337048	235060	472126
**17**	4731308	1662092	919518	533459	1220885	1038858	1605374	1136067	725771	2045836	393957	11657972
**19**	1690755	1096634	593988	376191	694269	567770	872046	583080	276485	566205	380358	7174363
**20**	3400*	3400*	3400*	3400*	3400*	3400*	3400*	3400*	3400*	3400*	3400*	635057
**22**	153824	126149	116886	83802	52492	71816	177935	131352	47218	93224	52360	571579
**23**	1863520	1693657	832024	676149	782413	1146345	1428193	1079132	498389	1451812	735445	5543102
**24**	392873	631673	192640	348560	239456	281681	303880	974572	102258	662634	211448	724874
**26**	362023	130821	135200	117157	166565	266458	240408	99853	62655	237295	155139	1399370
**30**	136901	35237	25917	16085	19342	24093	41639	31926	18972	41795	3400*	1091192
**33**	189790	50216	43185	59953	41795	30543	52713	63620	34159	54659	58150	473815
**35**	6014598	2146759	1100882	685711	939887	1515439	2303204	1383751	707293	2532928	459037	19002344
**42**	4284828	1251491	571410	725409	862704	958432	1386846	716032	361203	1334293	390485	36536915
**45**	260713	87435	49191	89162	55616	82610	102763	109371	21953	69462	149627	590549
**49**	72082	35303	27496	24509	31146	20784	34279	32704	7350	46674	17329	126399
**50**	190169	3400*	232639	3400*	348155	355868	3400*	6907	135364	3400*	362948	3986645
**64**	4231824	2130422	1237578	1737192	1475089	1908541	2614240	1333316	731441	1909602	1054486	19169040
**67**	20702	119436	66271	183689	49393	76753	137860	159094	3400*	140627	88015	362653
**71**	472376	287434	146411	329358	157311	169237	209568	282677	205773	197245	214246	567680
**92**	101510	36743	25168	43967	26273	34320	41865	33899	35971	74969	39171	184084
**94**	209606	278910	210798	204543	151520	207405	261172	346156	248025	219035	208088	745170
**95**	40161	47346	67717	31560	76296	161301	90450	79728	100375	227738	136649	39949
**96**	1048673	848752	833964	583538	896301	1760036	1186079	792172	1153657	1963731	1474996	1103037
**98**	83655	66810	3400*	71098	19757	3400*	24464	74100	21156	94637	3400*	3400*
**99**	271686	510639	55346	466517	107719	86793	137806	437938	79753	486880	66329	277122
**102**	253946	140318	87333	134232	95153	96632	222702	121057	94501	125287	87087	197216

Peaks are numbered according to Table 2. *: Not detected. A peak area of 3400 was considered, which corresponded to the half of the area obtained for the lowest detected peak.

Table S2: Spearman correlation coefficients among maize flours' volatile compounds.

V	6	7	12	14	17	19	22	23	24	26	30	33	35	42	45	49	50	64	67	71	92	94	95	96	98	99	102
5	0.070	0.661*	0.551	0.449	0.571	0.415	0.695*	0.620*	0.419	0.621*	0.563	0.368	0.614*	0.527	0.269	0.667*	-0.478	0.609*	0.316	0.234	0.565	0.192	0.222	0.252	0.513	0.326	0.642*
6		-0.214	0.076	0.268	-0.236	-0.184	-0.047	0.040	-0.274	0.380	-0.252	-0.241	-0.145	-0.143	0.094	-0.219	0.324	0.042	0.224	-0.311	-0.095	-0.223	0.473	0.587	-0.443	-0.332	-0.011
7			0.255	0.383	0.964**	0.933**	0.711*	0.875**	0.338	0.751**	0.951**	0.807**	0.969**	0.940**	0.681*	0.921**	-0.237	0.923**	-0.154	0.667*	0.760**	0.128	-0.200	0.003	0.557	0.291	0.844**
12				0.534	0.083	0.199	0.782**	0.491	0.576	0.042	0.107	-0.016	0.175	0.047	0.035	0.264	-0.667*	0.209	0.649*	0.082	0.019	0.634*	-0.053	-0.108	0.333	0.466	0.324
14					0.293	0.408	0.466	0.652*	0.363	0.457	0.182	0.007	0.315	0.212	0.110	0.463	-0.022	0.316	0.211	-0.213	0.107	0.047	0.324	0.344	0.086	0.137	0.178
17						0.911**	0.566	0.802**	0.208	0.779**	0.983**	0.888**	0.990**	0.981**	0.740**	0.932**	-0.117	0.910**	-0.287	0.698*	0.866**	-0.039	-0.150	0.069	0.543	0.224	0.799**
19							0.676*	0.851**	0.199	0.693*	0.894**	0.802**	0.911**	0.914**	0.729*	0.866**	-0.094	0.911**	-0.223	0.673*	0.641*	0.027	-0.385	-0.144	0.407	0.202	0.804**
22								0.731*	0.416	0.419	0.605*	0.476	0.632*	0.559	0.455	0.638*	-0.547	0.678*	0.298	0.450	0.362	0.486	-0.334	-0.233	0.391	0.307	0.797**
23									0.475	0.706*	0.746**	0.580	0.844**	0.769**	0.580	0.826**	-0.337	0.836**	0.107	0.512	0.648*	0.238	0.000	0.188	0.553	0.444	0.738**
24										-0.024	0.176	0.143	0.224	0.130	0.164	0.397	-0.601	0.147	0.601	0.312	0.197	0.696*	0.070	-0.057	0.731*	0.802**	0.160
26											0.744**	0.641*	0.807**	0.810**	0.672*	0.743**	0.189	0.848**	-0.177	0.364	0.725*	-0.318	0.203	0.453	0.192	-0.058	0.663*
30												0.926**	0.982**	0.982**	0.773**	0.897**	-0.132	0.909**	-0.291	0.758**	0.851**	0.013	-0.245	-0.019	0.516	0.189	0.825**
33													0.879**	0.927**	0.908**	0.833**	-0.045	0.828**	-0.235	0.870**	0.843**	-0.041	-0.342	-0.131	0.508	0.191	0.758**
35														0.981**	0.762**	0.922**	-0.163	0.939**	-0.222	0.711*	0.871**	0.020	-0.132	0.102	0.539	0.240	0.839**
42															0.816**	0.907**	-0.071	0.953**	-0.263	0.766**	0.864**	-0.095	-0.230	0.021	0.498	0.190	0.840**
45																0.715*	0.078	0.778**	-0.089	0.799**	0.726*	0.009	-0.241	-0.008	0.355	0.125	0.703*
49																	-0.227	0.878**	-0.002	0.659*	0.825**	-0.022	-0.131	0.012	0.662*	0.406	0.761**
50																		-0.163	-0.631*	-0.335	-0.206	-0.639*	0.153	0.251	-0.711*	-0.768**	-0.398
64																			-0.066	0.719*	0.784**	-0.066	-0.244	0.015	0.472	0.237	0.910**
67																				0.043	-0.111	0.399	0.053	-0.110	0.426	0.665*	0.049
71																					0.712*	0.197	-0.513	-0.342	0.676*	0.494	0.713*
92																						-0.102	0.105	0.285	0.661*	0.343	0.701*
94																							-0.111	-0.193	0.334	0.433	0.152
95																								0.937**	-0.074	-0.086	-0.331
96																									-0.103	-0.175	-0.113
98																										0.905**	0.475
99																											0.241

P: Peak (numbered according to Table 2); p-Value corresponds to the significance level of Spearman correlation coefficient indicated as *: significant at $p < 0.05$; **: significant at $p < 0.01$.

Table S3: Major soluble phenolic compounds and total carotenoids content of maize flours and *broas*.

Sample	pCA	FA	DCS*ct*	DCS*tt*	DCSτ	DFP*cc*	DFP*ct*	DFP*tt*	DFPτ	CFP*ct*	CFP*tt*	CFPτ	*bis*DFP	Carot
**F1**	0.41 ± 0.01	0.42 ± 0.03	0.82 ± 0.10	2.73 ± 0.50	3.55 ± 0.40	0.06 ± 0.01	1.52 ± 0.09	3.30 ± 0.79	4.89 ± 0.69	0.30 ± 0.03	0.89 ± 0.14	1.18 ± 0.17	0.07 ± 0.00	27.01 ± 0.84
**B1**	1.53 ± 0.19	1.80 ± 0.22	0.44 ± 0.01	1.65 ± 0.20	2.08 ± 0.20	0.06 ± 0.01	0.75 ± 0.01	2.26 ± 0.41	3.08 ± 0.41	0.19 ± 0.01	0.60 ± 0.09	0.79 ± 0.09	< 0.01	8.83 ± 0.03
**F2**	0.26 ± 0.02	0.33 ± 0.02	0.86 ± 0.03	1.80 ± 0.30	2.66 ± 0.27	0.17 ± 0.06	2.26 ± 0.01	3.27 ± 0.98	5.70 ± 0.93	0.37 ± 0.01	0.95 ± 0.19	1.33 ± 0.19	0.08 ± 0.02	46.01 ± 0.05
**B2**	1.02 ± 0.04	1.30 ± 0.09	0.35 ± 0.01	1.26 ± 0.11	1.61 ± 0.12	0.06 ± 0.01	0.97 ± 0.04	3.12 ± 0.53	4.15 ± 0.56	0.19 ± 0.01	0.81 ± 0.13	1.00 ± 0.13	< 0.01	15.21 ± 0.33
**F3**	0.36 ± 0.02	0.37 ± 0.03	0.86 ± 0.17	2.61 ± 0.50	3.46 ± 0.67	0.07 ± 0.01	2.09 ± 0.37	5.10 ± 0.51	7.26 ± 0.89	0.32 ± 0.05	1.09 ± 0.13	1.40 ± 0.18	0.11 ± 0.01	10.83 ± 0.35
**B3**	1.51 ± 0.11	1.44 ± 0.10	0.37 ± 0.03	1.50 ± 0.13	1.87 ± 0.15	0.05 ± 0.01	0.81 ± 0.11	3.74 ± 0.39	4.60 ± 0.49	0.15 ± 0.02	0.81 ± 0.09	0.96 ± 0.10	< 0.01	5.20 ± 0.08
**F4**	0.16 ± 0.00	0.28 ± 0.02	0.39 ± 0.02	0.61 ± 0.11	0.99 ± 0.09	0.15 ± 0.05	1.39 ± 0.12	1.98 ± 0.42	3.52 ± 0.25	0.25 ± 0.01	0.75 ± 0.10	0.99 ± 0.11	0.06 ± 0.00	33.49 ± 0.22
**B4**	0.75 ± 0.04	1.21 ± 0.08	0.22 ± 0.06	0.94 ± 0.10	1.16 ± 0.11	0.05 ± 0.01	0.81 ± 0.06	3.47 ± 0.57	4.33 ± 0.54	0.17 ± 0.01	1.08 ± 0.16	1.25 ± 0.15	< 0.01	11.63 ± 0.32
**F5**	0.38 ± 0.01	0.43 ± 0.02	0.76 ± 0.24	2.38 ± 0.48	3.13 ± 0.72	0.07 ± 0.01	1.86 ± 0.38	4.65 ± 0.13	6.58 ± 0.27	0.32 ± 0.07	0.98 ± 0.00	1.30 ± 0.07	0.10 ± 0.00	28.52 ± 0.15
**B5**	1.31 ± 0.08	1.46 ± 0.08	0.42 ± 0.03	1.86 ± 0.24	2.28 ± 0.27	0.05 ± 0.01	0.98 ± 0.02	4.21 ± 0.61	5.24 ± 0.63	0.22 ± 0.00	0.95 ± 0.11	1.16 ± 0.11	< 0.01	10.17 ± 0.46
**F6**	0.27 ± 0.00	0.36 ± 0.01	0.83 ± 0.07	1.78 ± 0.10	2.61 ± 0.17	0.11 ± 0.01	1.69 ± 0.31	2.45 ± 0.30	4.26 ± 0.62	0.33 ± 0.05	0.80 ± 0.12	1.14 ± 0.17	0.07 ± 0.00	10.10 ± 0.02
**B6**	1.05 ± 0.05	1.25 ± 0.06	0.40 ± 0.02	1.02 ± 0.06	1.43 ± 0.07	0.08 ± 0.01	0.87 ± 0.03	1.88 ± 0.15	2.83 ± 0.18	0.16 ± 0.00	0.57 ± 0.04	0.73 ± 0.04	< 0.01	4.44 ± 0.02
**F7**	0.33 ± 0.05	0.39 ± 0.03	0.69 ± 0.01	2.11 ± 0.03	2.79 ± 0.01	0.08 ± 0.00	2.13 ± 0.03	4.97 ± 0.07	7.18 ± 0.10	0.36 ± 0.00	1.32 ± 0.06	1.68 ± 0.06	0.06 ± 0.01	17.67 ± 0.02
**B7**	0.98 ± 0.09	1.36 ± 0.11	0.37 ± 0.05	1.13 ± 0.59	1.49 ± 0.61	0.05 ± 0.00	0.91 ± 0.17	3.55 ± 0.27	4.51 ± 0.40	0.18 ± 0.01	0.90 ± 0.07	1.08 ± 0.08	< 0.01	7.24 ± 0.06
**F8**	0.24 ± 0.01	0.28 ± 0.04	0.70 ± 0.12	1.72 ± 0.37	2.41 ± 0.48	0.12 ± 0.01	1.91 ± 0.16	3.42 ± 0.36	5.45 ± 0.50	0.29 ± 0.02	0.81 ± 0.07	1.09 ± 0.09	0.08 ± 0.00	61.39 ± 0.53
**B8**	0.97 ± 0.14	1.14 ± 0.15	0.47 ± 0.03	1.15 ± 0.23	1.62 ± 0.26	0.13 ± 0.02	1.38 ± 0.11	2.40 ± 0.61	3.91 ± 0.69	0.21 ± 0.01	0.60 ± 0.12	0.82 ± 0.13	< 0.01	19.47 ± 0.66
**F9**	0.27 ± 0.01	0.42 ± 0.02	0.63 ± 0.06	0.79 ± 0.14	1.41 ± 0.20	0.38 ± 0.01	3.22 ± 0.37	4.45 ± 0.71	8.05 ± 1.09	0.37 ± 0.10	1.08 ± 0.13	1.45 ± 0.23	0.07 ± 0.00	9.15 ± 0.55
**B9**	0.93 ± 0.02	1.19 ± 0.02	0.34 ± 0.02	0.83 ± 0.05	1.17 ± 0.06	0.10 ± 0.02	1.62 ± 0.22	4.43 ± 0.57	6.15 ± 0.63	0.21 ± 0.02	0.95 ± 0.10	1.15 ± 0.10	< 0.01	3.31 ± 0.05
**F10**	0.26 ± 0.00	0.37 ± 0.02	0.77 ± 0.07	1.28 ± 0.09	2.05 ± 0.15	0.21 ± 0.02	2.05 ± 0.12	2.27 ± 0.15	4.53 ± 0.29	0.35 ± 0.03	0.69 ± 0.07	1.04 ± 0.10	0.09 ± 0.01	50.74 ± 0.37
**B10**	0.87 ± 0.06	1.24 ± 0.09	0.35 ± 0.03	0.99 ± 0.08	1.35 ± 0.11	0.08 ± 0.00	0.97 ± 0.06	2.36 ± 0.20	3.41 ± 0.26	0.16 ± 0.01	0.63 ± 0.05	0.79 ± 0.05	< 0.01	15.54 ± 0.36
**F11**	0.30 ± 0.04	0.39 ± 0.06	0.92 ± 0.04	2.40 ± 0.31	3.33 ± 0.35	0.06 ± 0.01	1.63 ± 0.09	2.88 ± 0.44	4.58 ± 0.51	0.27 ± 0.03	0.65 ± 0.07	0.92 ± 0.10	0.12 ± 0.01	8.16 ± 0.15
**B11**	1.30 ± 0.10	1.67 ± 0.13	0.51 ± 0.08	1.48 ± 0.14	1.99 ± 0.21	0.07 ± 0.02	1.04 ± 0.17	2.67 ± 0.24	3.78 ± 0.38	0.17 ± 0.05	0.58 ± 0.05	0.75 ± 0.07	< 0.01	3.53 ± 0.31
**F12**	0.28 ± 0.02	0.34 ± 0.02	0.53 ± 0.06	1.17 ± 0.13	1.70 ± 0.19	0.06 ± 0.02	0.76 ± 0.05	1.05 ± 0.04	1.87 ± 0.11	0.22 ± 0.01	0.44 ± 0.05	0.66 ± 0.05	0.06 ± 0.00	2.11 ± 0.51
**B12**	0.62 ± 0.06	1.00 ± 0.12	0.25 ± 0.10	0.57 ± 0.11	0.82 ± 0.17	0.07 ± 0.01	0.46 ± 0.02	0.69 ± 0.08	1.22 ± 0.07	0.13 ± 0.01	0.29 ± 0.05	0.42 ± 0.05	< 0.01	1.21 ± 0.17

F: Maize flour; **B**: *Broa*; **pCA**: *p*-Coumaric acid (mg 100 g^{-1} dw [dry weight]); **FA**: Ferulic acid (mg 100 g^{-1} dw); **DCS**: Dicoumaroyl spermidine (mg pCA equivalents 100 g^{-1} dw); **DFP**: Diferuloyl putrescine (mg ferulic acid equivalents [FAE] 100 g^{-1} dw); **CFP**: Coumaroyl feruloyl putrescine (mg FAE 100 g^{-1} dw); ***bis*DFP**: *bis*-Diferuloyl putrescine (mg FAE 100 g^{-1} dw); ***cc***: *cis,cis* isomer; ***ct***: *cis.trans* isomer; ***tt***: *trans,trans* isomer; τ: Total of isomeric forms; **bisDFP**: *Bis*-diferuloyl putrescine; **Carot**: Total carotenoids content (mg lutein equivalentes 100 g^{-1}).

Table S4: Spearman correlation coefficients between maize flours' volatile compounds and the content in major phenolics and total carotenoids.

V \ Var	pCA	FA	DCS*ct*	DCS*tt*	DCS$_T$	DFP*cc*	DFP*ct*	DFP*tt*	DFP$_T$	CFP*ct*	CFP*tt*	CFP$_T$	bisDFP	Carot
**5**	0.05	-0.20	-0.35	-0.10	-0.10	-0.18	-0.28	-0.24	-0.38	-0.19	-0.20	-0.20	-0.45	0.13
**6**	-0.05	-0.16	0.24	0.03	0.03	-0.19	-0.28	-0.39	-0.50	-0.12	-0.38	-0.33	-0.04	-0.27
**7**	0.19	-0.07	-0.14	0.10	0.10	-0.20	-0.07	-0.11	-0.17	0.08	-0.01	0.05	-0.43	0.12
**12**	-0.28	-0.64*	-0.21	-0.19	-0.19	-0.02	0.02	-0.14	-0.20	-0.11	-0.07	-0.08	-0.39	0.14
**14**	0.11	-0.17	0.13	0.09	0.09	-0.13	0.05	-0.17	-0.20	0.15	-0.10	-0.03	-0.06	0.04
**17**	0.21	0.02	-0.12	0.09	0.09	-0.17	-0.10	-0.17	-0.20	0.07	-0.08	-0.03	-0.28	0.19
**19**	0.48	0.02	0.08	0.43	0.43	-0.54	-0.17	0.09	-0.04	-0.09	0.11	0.09	-0.19	-0.01
**20**	0.04	-0.22	-0.39	-0.31	-0.31	-0.40	-0.48	-0.48	-0.48	-0.48	-0.48	-0.48	-0.40	-0.48
**22**	0.10	-0.33	-0.20	0.12	0.12	-0.32	-0.17	-0.05	-0.18	-0.20	0.01	-0.03	-0.45	0.15
**23**	0.16	-0.17	0.11	0.20	0.20	-0.29	-0.18	-0.27	-0.34	-0.01	-0.20	-0.13	-0.26	0.09
**24**	-0.43	-0.60*	-0.26	-0.28	-0.28	0.04	-0.31	-0.50	-0.55	-0.29	-0.47	-0.47	-0.31	0.52
**26**	0.52	0.23	0.08	0.32	0.32	-0.62*	-0.50	-0.28	-0.45	-0.21	-0.28	-0.24	-0.25	-0.34
**30**	0.18	-0.09	-0.11	0.10	0.10	-0.18	-0.06	-0.12	-0.17	0.05	-0.05	0.00	-0.32	0.15
**33+34**	-0.09	-0.33	-0.28	-0.06	-0.06	-0.41	-0.62*	-0.44	-0.52	-0.71**	-0.54	-0.62*	-0.27	0.11
**35**	0.17	-0.07	-0.09	0.07	0.07	-0.17	-0.10	-0.20	-0.25	0.08	-0.10	-0.02	-0.38	0.08
**42**	0.19	-0.06	-0.18	0.06	0.06	-0.28	-0.32	-0.34	-0.43	-0.06	-0.19	-0.14	-0.50	0.08
**45**	0.05	-0.27	-0.09	0.11	0.11	-0.60*	-0.66*	-0.44	-0.57	-0.64*	-0.53	-0.57	-0.34	-0.13
**49**	0.14	-0.14	-0.12	0.11	0.11	-0.24	-0.21	-0.22	-0.28	-0.12	-0.17	-0.16	-0.25	0.29
**50**	0.49	0.27	0.23	0.28	0.28	-0.71*	-0.49	-0.11	-0.23	-0.47	-0.34	-0.36	0.22	-0.74**
**64+65**	0.12	-0.16	-0.19	0.03	0.03	-0.25	-0.33	-0.37	-0.45	-0.08	-0.19	-0.14	-0.56	0.08
**67**	-0.60*	-0.78**	-0.40	-0.52	-0.52	0.05	-0.37	-0.59*	-0.64*	-0.47	-0.56	-0.60*	-0.36	0.24
**71**	-0.24	-0.39	-0.35	-0.24	-0.24	-0.20	-0.50	-0.55	-0.50	-0.47	-0.46	-0.46	-0.55	0.00
**92**	-0.07	-0.08	-0.34	-0.25	-0.25	-0.16	-0.48	-0.66*	-0.60*	-0.29	-0.54	-0.47	-0.54	-0.12
**94**	-0.25	-0.36	-0.20	-0.24	-0.24	0.14	0.31	-0.01	0.10	0.14	0.01	0.06	-0.26	0.02
**95**	-0.06	0.29	0.35	0.03	0.03	0.30	0.41	0.12	0.17	0.47	0.00	0.12	0.39	-0.05
**96**	0.18	0.41	0.23	0.03	0.03	-0.01	0.08	-0.20	-0.15	0.32	-0.23	-0.06	0.06	-0.39
**98**	-0.35	-0.13	-0.23	-0.17	-0.17	0.45	0.12	-0.09	-0.03	0.17	0.02	0.03	-0.16	0.80**
**99**	-0.596*	-0.56	-0.30	-0.43	-0.43	0.40	-0.08	-0.50	-0.42	0.00	-0.31	-0.28	-0.34	0.69*
**102**	-0.01	-0.20	-0.38	-0.08	-0.08	-0.07	-0.22	-0.26	-0.30	-0.03	-0.03	-0.01	-0.71*	0.26

Var: Variables; **V**: Maize flours' volatiles (peaks numbered according to Table 2); **TPC**: Total phenolic content; **AA**: Antioxidant activity: **pCA**: *p*-Coumaric acid; **FA**: Ferulic acid; **DCS**: Dicoumaroyl spermidine; **DFP**: Diferuloyl putrescine; **CFP**: Coumaroyl feruloyl putrescine; ***cc***: *cis,cis* isomer; ***ct***: *cis.trans* isomer; ***tt***: *trans,trans* isomer; T: Total of isomeric forms; **bisDFP**: *Bis*-diferuloyl putrescine; **Carot**: Carotenoids. *p*-Value corresponds to the significance level of Spearman correlation coefficient indicated as *: significant at $p < 0.05$; **: significant at $p < 0.01$.

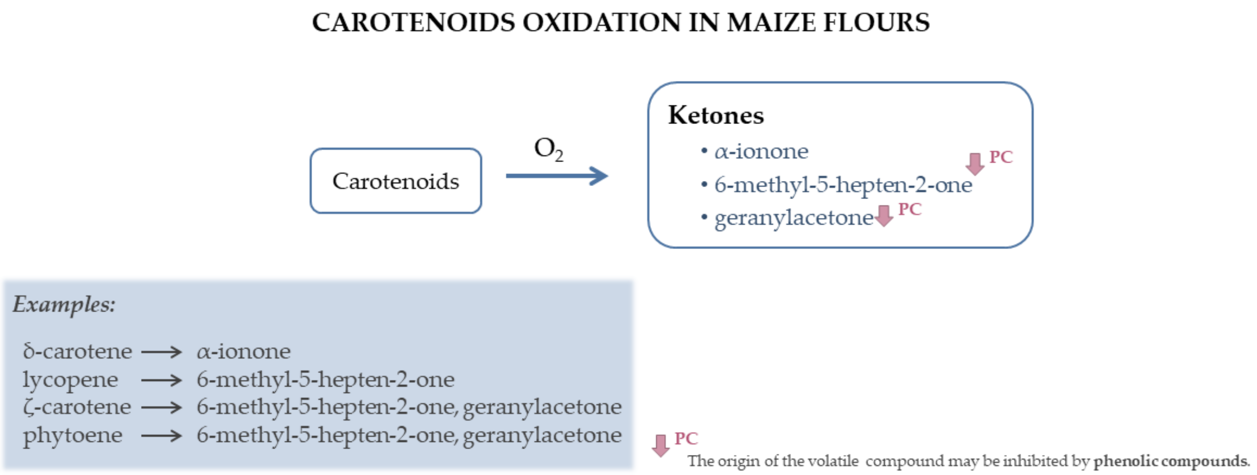

Figure S3: Representative scheme of carotenoids oxidation reactions [6,7] occurring in maize flour samples.

3. *Broas'* volatile compounds

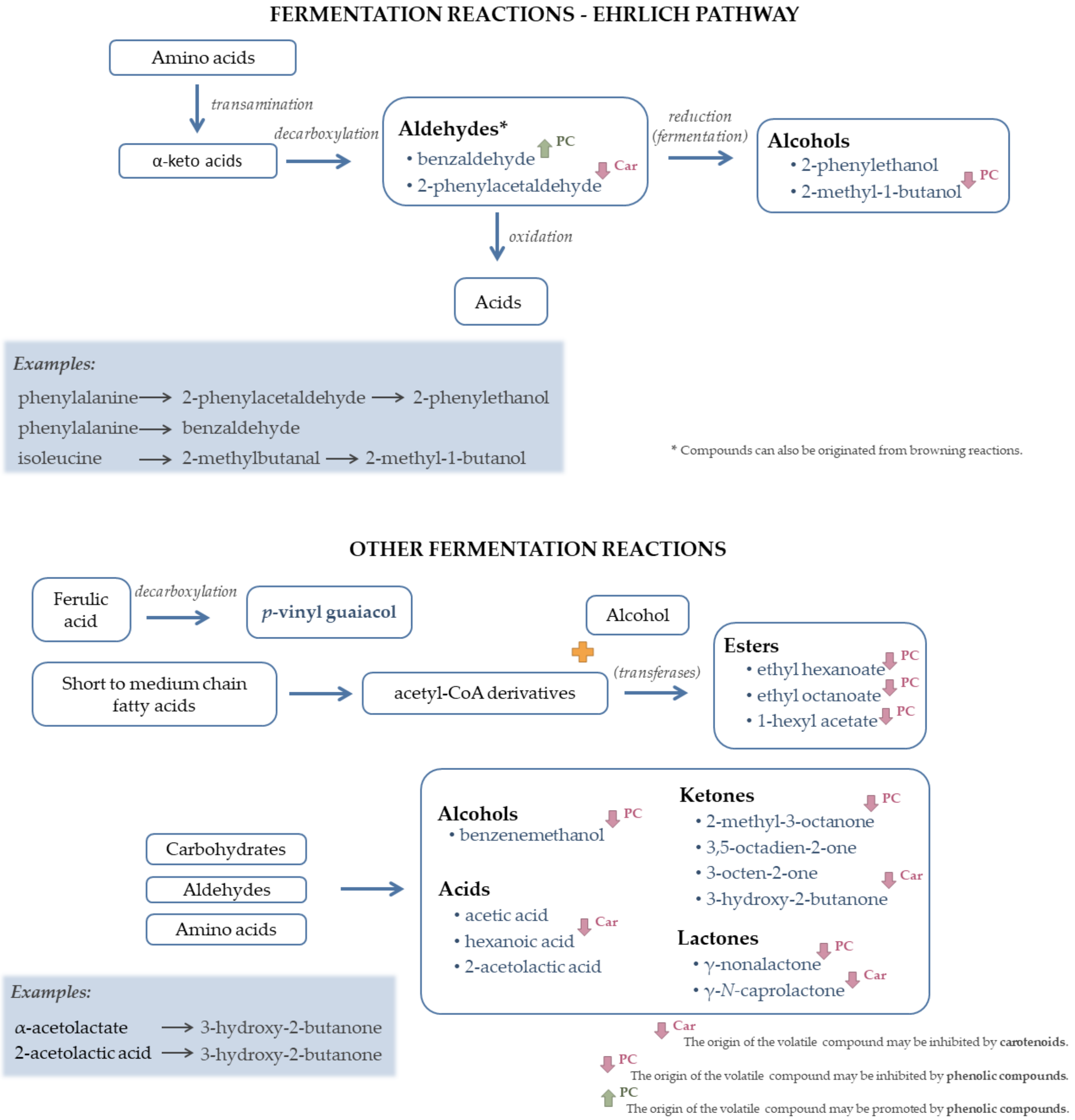

Figure S4: Representative scheme of fermentation [1,2,8–11] reactions in *broas*.

MAILLARD REACTION – EARLY STAGES

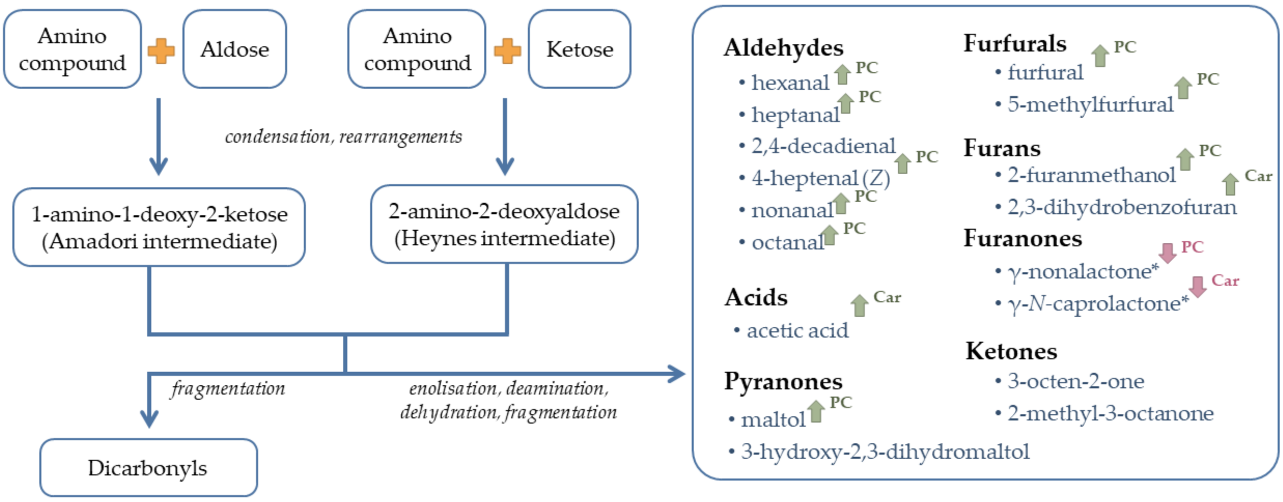

* The compound can also be originated from lipid oxidation reactions and/or during fermentation.

MAILLARD REACTION – STRECKER DEGRADATION

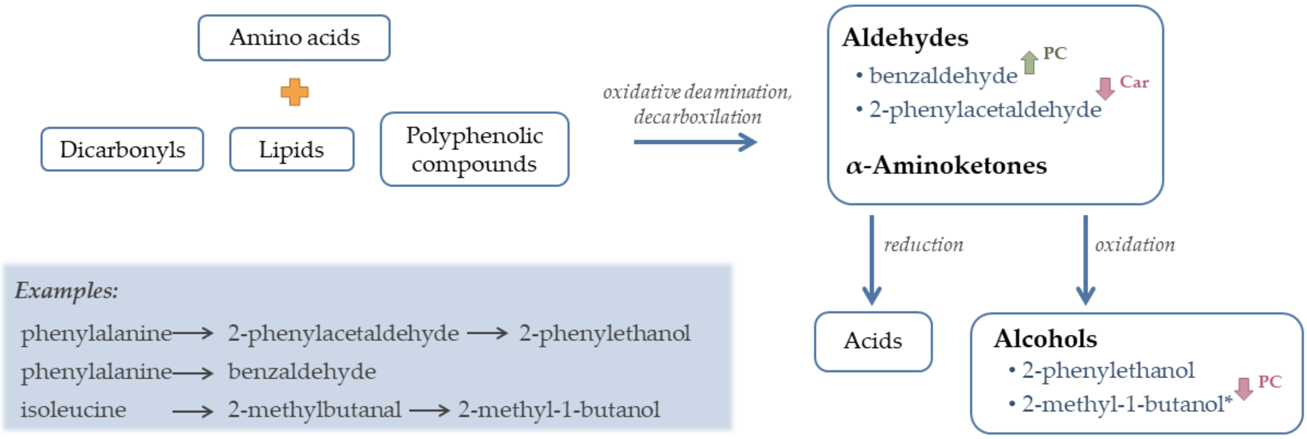

* The compound can also be originated by the Ehrlich pathway during fermentation.

MAILLARD REACTION – LAST STAGES

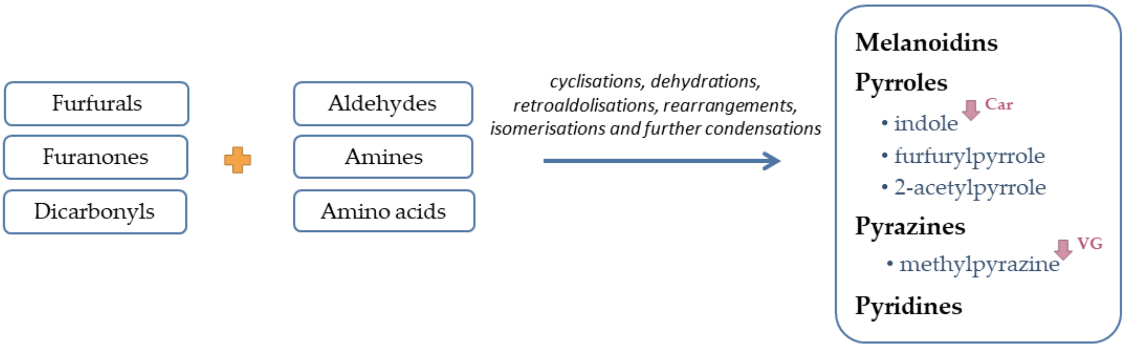

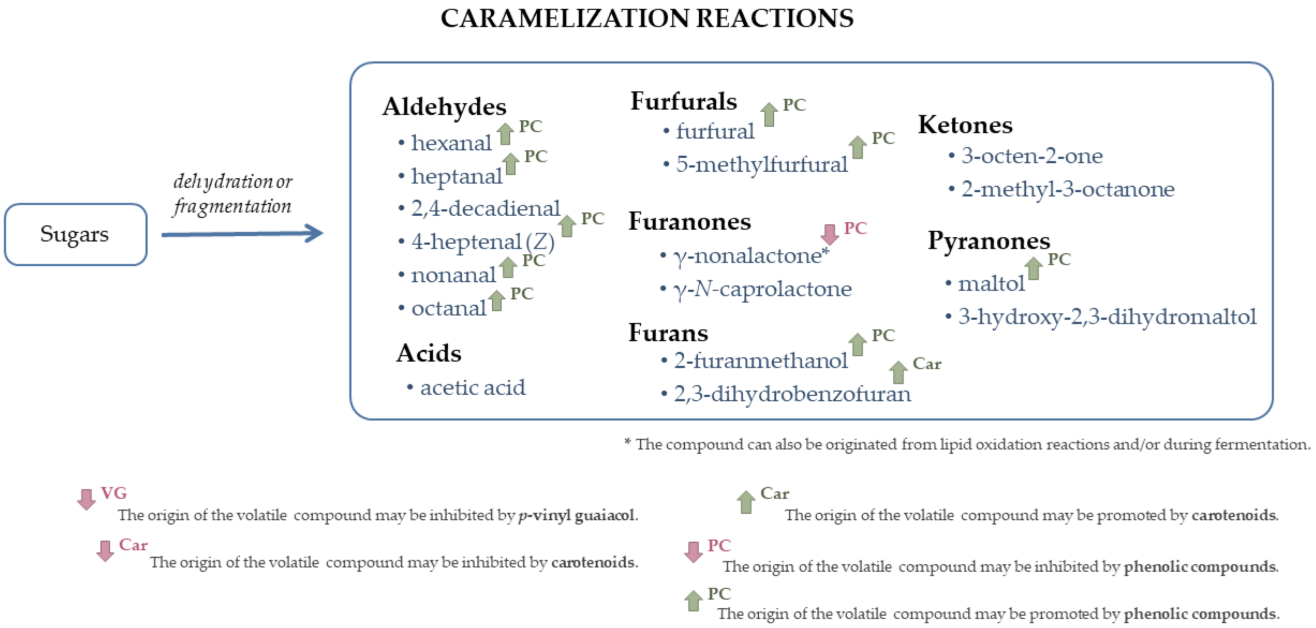

Figure S5: Representative scheme of non-enzymatic browning reactions [8–10,12–19] in *broas*.

Table S5: Average peak areas obtained for each *broa* and considered for the cluster analysis.

Peak	B1	B2	B3	B4	B5	B6	B7	B8	B9	B10	B11	B12
1	4052460	6913655	3400	5878768	3059599	3436765	2782261	3277620	3400	4707432	1877325	3400*
2	1613111	1620132	3900906	2643630	1468109	2776033	3882538	1373954	6166869	2463561	30935558	3394857
3	42809453	39384900	33613872	34737284	26545801	28408077	25012446	32308492	30243685	36939700	31307981	40425174
4	1391288	1847294	2180311	11175028	1462992	2194962	2376009	3637860	1917772	1856100	2435142	2685667
5	3593791	3686261	3972399	4820196	2761784	2702190	3046064	2477990	2769137	3338531	3405233	5004677
6	1291798	1471542	1850174	2083018	1177605	1413696	2148679	1968306	2227936	2065355	2722728	1925435
7	41683618	27962542	10011568	6495596	28208587	23023617	14924908	6775093	33668744	15145745	7077303	11052841
8	198480	193930	311064	181077	445379	303176	485323	253979	213735	148576	140761	709668
9	34897282	26314065	26882267	2273011	37324793	52388869	35154083	15673955	22721271	12099642	9750833	7089576
10+11	8031932	15653699	32243251	15761638	17793289	24282608	25086285	22056034	16016763	12749030	19141693	8549643
12	37377696	38822350	51998922	60982592	33481342	32710156	29479398	24333969	30882438	35750860	49383172	63521909
13	1164009	1349253	1255192	1672298	1106670	864794	977344	1018060	1306491	1202917	954805	1054406
14	2149843	1955902	2463292	6311790	1685502	1452278	1903953	1764546	1969608	2341862	2434417	3421223
15	113944	134862	261796	415480	178634	191245	214951	452640	186123	211217	223172	248711
16	446277	283641	74232	3400*	317254	152965	148377	32667	390962	238175	98585	105878
17	32489764	21278051	10737694	3468487	23923175	14278293	13260280	6812942	23952379	17195874	7717869	11536969
18	1735450	2057507	4662797	2570542	3061797	3857207	3131023	3733842	2249823	1377301	1731491	1151421
19	1623548	1465513	1290231	1566770	1111399	1562207	1337130	1225524	1277881	1355121	956488	2258959
20	3144333	2446348	1497460	458360	2525714	3378573	2520405	1111221	2636028	1459251	1263346	1507561
21	2595959	1737963	3879275	267453	2325498	5775428	3852010	2784448	1749929	1171298	1173912	1415271
22	4311779	2332057	4041739	9207260	2756138	2029140	2017175	2113637	2069868	3220536	5199669	7341500
23	3768645	3048444	3083768	3935878	2703577	3354148	3269097	3549377	2973530	3283096	3414662	5159205
25	2051450	2148189	1800744	1915352	1709455	1535459	1701481	1643097	2286783	2099377	1189440	2142055
26	31676334	28989143	27251115	33325932	25999215	23244700	24141763	21986265	30210938	28265876	23382118	28739581
27	4953777	5037512	5052697	4710193	4735489	5259447	5154612	3933640	5574949	5467128	4887885	5750972

**28**	100780	81779	158659	114860	206489	283017	252524	182043	177354	194603	193142	182601
**29**	462177	353456	390378	522654	325111	379800	271951	247557	336935	394589	345915	448016
**30**	4188404	3020244	2089020	739941	2897347	3273912	2690390	1588579	3239119	3107094	2130140	3773725
**32**	298606	218204	429272	2468688	135767	195278	145557	91797	93674	884731	1833196	2194559
**33+34**	621269	483667	696457	744337	534387	468188	510564	633148	667322	486793	656622	1222280
**35**	8302680	6047662	2618490	1973207	4356173	4162317	4602592	3059521	5098051	4959568	2473066	7522055
**36**	98102	84972	311543	191446	72314	392731	326022	381897	459506	364234	288649	997204
**37**	587522	755951	297704	272196	486277	471636	529033	268413	484596	613623	297630	715258
**38+39**	1598227	1802087	1647842	1514611	1446428	1739374	1662019	1320210	1900977	1780575	2046604	2916241
**41**	59738	46123	350006	227259	290495	432200	351480	168764	276482	268761	412327	373893
**42**	8695448	5976580	5714487	6971412	6441891	7782569	7318207	7045146	7957922	7468878	5688109	9934246
**43**	174532	400291	317189	330852	324537	424027	501831	392050	347390	325182	395272	313054
**44**	238209	193748	259734	567444	285827	385678	374888	303588	209579	249207	317469	408199
**45**	1535769	893236	1295200	1928160	1294623	1588531	1619058	1512016	1233514	1319706	1268380	1928022
**46**	376254	178579	438269	319181	332838	878159	666754	640722	107136	3400*	3400*	3400*
**47**	1268841	1442951	1048583	1195958	1184681	994363	1221829	1195409	1635686	1676642	1310337	472280
**51**	976209	1257881	1127429	1378532	1040453	1502233	1252689	1081818	1171470	1222260	1297878	961650
**52**	1503007	283172	2068925	1164557	819799	4132068	2798608	2812151	446526	265389	508945	367096
**53**	15152537	11609414	8828916	5020070	10862974	12566136	13518938	6185419	13483629	10616912	12359645	8839444
**54**	540800	5129324	6599299	2262882	6032707	13884318	6531884	15783039	4701947	6569573	9450744	400543
**55+56**	8450949	14285894	18616582	14589386	12678874	14041615	10873452	13614795	13201407	12280016	15421156	16179171
**57**	20041	3400*	93277	31646	45008	225202	169023	143060	19116	10995	22289	17029
**58**	1011329	2516272	973607	1094614	1101677	1548085	850607	1543379	598885	813143	460842	1151660
**59**	1412029	2624523	1393247	1607556	1273287	1900710	1314660	1708459	1054199	1137389	1091069	1052786
**60+61**	718680	4156615	3168889	1902561	2752217	5673703	3021665	9812370	834929	3902311	8548717	2165964
**62**	1863121	3400*	1733556	2152229	2313533	1720956	1831554	1804888	2133698	2657191	2395628	3474196
**63**	51189	3400*	120961	44869	20411	347098	209081	191003	10491	3400*	3400*	14602
**64+65**	6339166	5642795	3417544	2707100	4269184	5385376	5911011	3113892	5152787	4961316	4767411	5000905
**66**	74630	57873	158578	129116	292359	597594	642937	586295	255356	402545	458564	464240
**67**	438183	640477	515117	857963	450077	555275	586652	948866	604684	451767	646392	706207
**68**	95629	64393	109861	101058	72714	140880	124854	108057	78194	62189	65775	109235
**69**	2330773	1478157	1553139	2171127	1476484	1637940	1276584	1296740	1401814	1158143	1222309	1758262
**70**	2526163	3656598	4313685	3129476	2656395	3715235	3358593	3912802	2052527	2587557	2717313	3115555
**71**	3212529	2364740	1870765	1478061	3005185	2484220	2415535	1769081	2288348	2605161	2273039	1980932
**72**	2566407	138938	337016	1119054	220113	368650	309619	242157	84277	235418	466327	2533421
**73**	454356	621107	362993	797011	430308	737478	533188	779705	276825	536760	513292	221482
**74**	416078	317656	470235	383909	380686	729395	634770	624754	259102	172866	164255	239652
**75**	37184	58201	39284	20787	55507	42719	51099	28537	87530	73321	90025	28895
**76**	187247	76902	163165	159803	125155	322680	279348	270960	3400*	3400*	3400*	3400*
**77**	1613542	1520422	1193258	1115091	1266597	1592534	1611110	1365599	1679614	1771210	1273830	1953320
**78**	206958	136615	255803	222412	183613	178479	160586	103178	106388	128061	140773	176374
**79**	1829113	1773109	1673559	1972275	1575570	1675712	1596893	1385864	1898293	1581061	1798724	2975183
**80**	6635587	4319722	5277130	5450636	6300769	9207526	8274601	7910861	2274495	2209702	2223696	3286119
**81**	3906205	2154931	3052472	2992268	3601337	6323672	5602433	5357264	352941	317403	232080	574600
**82**	2831539	3293831	3296997	3952307	3270635	722472	3479460	2979964	3750725	3659228	4729299	1768510
**83**	3654169	1940086	3016515	3235971	3351406	5973824	5256780	5163522	503478	357077	419552	594503
**84**	3206104	3052760	2175799	2146376	2181693	2296852	3079223	2531329	3633080	3540861	2206438	3447821
**85**	20733	37824	25370	37319	124243	135042	178924	195143	162155	254445	240083	327804
**86**	39752	30773	3400*	28294	100447	92256	135648	148839	98077	171831	152263	183248
**87**	1524488	1369420	1115381	1385528	1237920	1296943	1385626	1281608	1520064	1604085	1009771	1981223

**89**	566510	772346	759028	638117	499344	589963	436567	508654	545421	419994	419998	770465
**90**	229693	305503	324023	304323	295081	273278	331966	318051	282425	380979	457308	476522
**91**	1320265	1308923	1191385	1699429	1491772	1579126	1491461	1586679	1566635	1528966	1417740	1918638
**92**	359465	272754	213875	178524	181084	229743	262689	221401	257947	252175	208731	253418
**94**	1488997	1156215	1069183	1328720	1399041	1496208	1585530	1631865	1555529	1818639	1884353	2487290
**95**	292897	451798	516811	472471	319294	246098	324599	278938	226035	253007	350927	247127
**96**	2098644	2928697	3068897	2813205	2532200	2209901	2515595	2274340	2319640	2493556	3335083	2744327
**98**	209390	167197	3400*	114012	113658	75642	84664	266659	102979	234135	68232	3400*
**99**	981776	1003767	522505	839491	774144	600560	722266	1225579	555970	1197652	479132	736538
**100**	194157	108831	90697	159954	74380	101802	84468	85110	3400*	3400*	3400*	3400*
**102**	352367	265805	222085	318640	326795	350290	323164	255323	267612	277907	349331	302100
**104**	290772	253259	243965	378274	395475	305004	390790	379881	195570	229084	261919	203555

Peaks are numbered according to Table 2. *: Not detected. A peak area of 3400 was considered, which corresponded to the half of the area obtained for the lowest detected peak.

Table S6: Spearman correlation coefficients among *broas'* volatile compounds.

V	2	3	4	5	6	7	8	9	10+11	12	13	14	15	16	17	18	19	20	21	22	23
**1**	-0.634*	0.324	-0.190	0.000	-0.289	0.070	-0.528	0.000	-0.373	-0.014	0.303	-0.148	-0.268	0.099	0.085	-0.092	0.345	-0.106	-0.275	0.035	0.127
**2**		-0.252	0.273	0.273	0.643*	-0.175	-0.014	-0.189	0.329	0.252	-0.112	0.406	0.196	-0.175	-0.189	-0.063	-0.147	0.000	-0.042	0.049	-0.070
**3**			-0.119	0.678*	-0.217	0.014	-0.308	-0.490	-0.762**	0.615*	0.455	0.587*	-0.056	0.042	0.056	-0.580*	.636*	-0.217	-0.427	0.643*	0.524
**4**				0.126	0.559	-0.881**	0.056	-0.580*	0.301	0.147	-0.224	0.336	0.888**	-0.881**	-0.902**	0.126	0.021	-0.643*	-0.168	0.210	.587*
**5**					0.056	-0.238	-0.091	-0.531	-0.427	0.916**	0.497	0.867**	0.154	-0.231	-0.210	-0.441	0.552	-0.315	-0.476	0.762**	0.392
**6**						-0.469	-0.350	-0.587*	0.168	-0.049	-0.007	0.385	0.462	-0.385	-0.434	-0.217	-0.308	-0.490	-0.413	0.028	0.119
**7**							0.105	0.601*	-0.357	-0.294	0.105	-0.448	-0.944**	0.986**	0.993**	-0.161	0.119	0.867**	0.224	-0.371	-0.497
**8**								0.441	0.315	-0.161	-0.308	-0.280	0.084	0.007	0.063	0.378	0.119	0.385	0.594*	-0.315	-0.112
**9**									0.413	-0.531	-0.329	-0.776**	-0.559	0.510	0.545	0.573	-0.126	.783**	0.811**	-0.678*	-0.552
**10+11**										-0.385	-0.399	-0.357	0.399	-0.434	-0.406	.832**	-0.559	-0.042	0.643*	-0.503	-0.357
**12**											0.364	0.825**	0.182	-0.287	-0.280	-0.427	0.434	-0.343	-0.510	.874**	0.406
**13**												0.455	-0.105	0.105	0.154	-0.133	0.224	-0.210	-0.441	0.315	-0.196
**14**													0.420	-0.406	-0.399	-0.469	0.329	-0.559	-0.615*	.839**	0.490
**15**														-0.951**	-0.937**	0.245	-0.119	-0.783**	-0.091	0.280	0.469
**16**															0.993**	-0.287	0.077	.797**	0.098	-0.322	-0.476
**17**																-0.224	0.105	.811**	0.154	-0.336	-0.503
**18**																	-0.287	0.126	0.769**	-0.517	-0.329
**19**																		0.196	-0.112	0.301	0.566
**20**																			0.545	-0.510	-0.378
**21**																				-0.643*	-0.273
**22**																					0.580*

V	25	26	27	28	29	30	32	33_34	35	36	37	38+39	41	42	43	44	45	46	47	51	52
**1**	0.077	0.246	-0.387	-0.261	0.289	-0.049	0.120	-0.542	0.035	-0.493	0.162	-0.331	-0.613*	-0.085	0.183	-0.127	0.085	0.085	0.331	0.394	-0.092
**2**	-0.035	0.000	0.497	0.154	0.035	-0.021	0.301	0.357	-0.210	0.385	-0.126	0.664*	0.643*	-0.098	0.203	0.168	-0.091	-0.254	0.070	0.322	-0.091
**3**	0.552	0.594*	0.077	-0.699*	0.727**	0.308	0.559	0.259	0.441	-0.077	0.406	0.182	-0.517	0.252	-0.594*	-0.224	0.105	-0.444	0.119	-0.336	-0.455
**4**	-0.364	-0.259	-0.161	0.133	-0.021	-0.538	0.280	0.503	-0.580*	0.455	-0.580*	0.021	0.322	-0.077	0.273	0.783**	0.538	0.028	-0.364	0.280	0.287
**5**	0.448	0.650*	0.161	-0.559	0.741**	0.014	.832**	0.545	0.119	-0.161	0.308	0.343	-0.098	-0.049	-0.462	0.126	0.224	-0.465	-0.070	-0.098	-0.434
**6**	-0.042	-0.021	0.126	0.077	-0.182	-0.378	0.168	0.350	-0.329	0.378	-0.322	0.399	0.203	-0.119	0.378	0.189	-0.014	-0.373	0.448	0.364	-0.182
**7**	0.441	0.294	0.364	-0.028	-0.035	0.762**	-0.413	-0.462	0.741**	-0.161	0.608*	0.126	-0.196	0.420	-0.154	-0.650*	-0.392	0.014	0.294	-0.273	-0.217
**8**	-0.042	-0.252	0.294	0.357	-0.266	0.182	-0.329	0.119	0.224	0.301	0.168	-0.119	0.378	0.315	-0.112	0.287	0.336	0.444	-0.720**	-0.483	0.371
**9**	-0.266	-0.336	0.042	0.357	-0.371	0.287	-0.615*	-0.643*	0.182	-0.217	0.147	-0.315	0.147	0.035	0.217	-0.252	-0.154	0.711**	-0.203	-0.014	0.503
**10+11**	-0.664*	-0.692*	-0.168	0.434	-0.580*	-0.552	-0.385	-0.105	-0.650*	0.105	-0.587*	-0.259	0.483	-0.490	0.524	0.238	-0.056	0.627*	-0.329	0.350	0.671*
**12**	0.259	0.497	0.063	-0.427	0.762**	-0.007	0.895**	0.538	-0.063	-0.196	0.161	0.336	0.070	-0.168	-0.503	0.231	0.161	-0.521	-0.210	-0.035	-0.420
**13**	0.755**	0.818**	-0.042	-0.755**	0.413	-0.147	0.210	0.280	0.119	-0.315	0.168	-0.084	-0.678*	-0.105	-0.350	-0.434	-0.217	-0.324	0.378	-0.042	-0.483
**14**	0.336	0.566	0.112	-0.469	0.692*	-0.161	0.846**	0.748**	-0.119	0.042	-0.014	0.315	-0.028	-0.049	-0.503	0.203	0.238	-0.592*	0.056	-0.077	-0.420
**15**	-0.357	-0.301	-0.224	0.070	-0.014	-0.685*	0.273	0.573	-0.664*	0.378	-0.636*	-0.161	0.231	-0.231	0.035	0.629*	0.441	0.049	-0.385	0.084	0.287
**16**	0.455	0.308	0.350	-0.021	-0.042	0.755**	-0.371	-0.462	0.755**	-0.182	0.636*	0.175	-0.224	0.406	-0.154	-0.671*	-0.427	-0.106	0.406	-0.273	-0.322
**17**	0.490	0.329	0.371	-0.049	-0.021	0.748**	-0.385	-0.448	0.762**	-0.175	0.643*	0.133	-0.245	0.413	-0.196	-0.692*	-0.413	-0.056	0.364	-0.308	-0.294
**18**	-0.476	-0.441	-0.308	0.175	-0.399	-0.455	-0.510	-0.161	-0.510	-0.035	-0.559	-0.608*	0.126	-0.308	0.329	0.126	0.063	0.866**	-0.434	0.224	0.811**
**19**	0.434	0.552	0.357	-0.280	0.769**	0.503	0.483	0.056	0.469	0.154	0.469	0.147	-0.154	0.615*	-0.294	0.224	0.622*	0.000	-0.224	-0.063	-0.077
**20**	0.147	0.049	0.434	0.217	-0.084	0.755**	-0.441	-0.455	0.580*	0.042	0.434	0.119	0.168	0.483	0.028	-0.308	-0.126	0.338	-0.077	-0.133	0.168
**21**	-0.392	-0.510	0.042	0.273	-0.406	0.098	-0.608*	-0.322	0.007	0.140	-0.154	-0.336	0.245	0.070	0.203	-0.056	0.035	0.859**	-0.462	-0.105	0.776**
**22**	0.147	0.476	-0.203	-0.434	0.720**	-0.105	0.839**	0.650*	-0.140	-0.238	-0.049	0.098	-0.084	-0.112	-0.657*	0.259	0.231	-0.570	-0.112	-0.196	-0.371

**23**	-0.147	0.112	-0.112	-0.140	0.545	0.042	0.573	0.413	-0.056	0.266	-0.168	0.021	0.035	0.357	-0.252	0.601*	0.727**	-0.099	-0.301	-0.070	0.147	
**25**		0.797**	0.483	-0.559	0.371	0.427	0.112	0.189	0.671*	0.084	0.629*	0.343	-0.503	0.406	-0.399	-0.510	-0.196	-0.500	0.371	-0.329	-0.692*	
**26**			0.154	-0.692*	0.671*	0.252	0.413	0.350	0.399	-0.231	0.371	0.126	-0.517	0.280	-0.490	-0.266	0.056	-0.416	0.329	-0.140	-0.503	
**27**				0.252	0.189	0.664*	0.105	-0.035	0.545	0.650*	0.580*	0.727**	0.399	0.580*	-0.049	-0.098	0.028	-0.275	0.042	-0.091	-0.357	
**28**					-0.406	0.084	-0.217	-0.364	-0.189	0.343	-0.070	0.063	0.741**	0.112	0.371	0.448	0.217	0.218	-0.266	0.175	0.266	
**29**						0.287	0.811**	0.301	0.140	-0.063	0.224	0.154	-0.119	0.280	-0.566	0.161	0.420	-0.310	-0.077	0.007	-0.266	
**30**							-0.028	-0.259	0.839**	0.273	0.699*	0.462	0.091	0.755**	-0.273	-0.273	0.000	-0.169	0.035	-0.329	-0.266	
**32**								0.413	-0.147	-0.084	0.112	0.357	0.154	-0.077	-0.350	0.392	0.364	-0.521	-0.063	0.168	-0.378	
**33_34**									-0.210	0.224	-0.336	0.084	0.084	0.035	-0.531	0.322	0.245	-0.352	-0.273	-0.364	-0.098	
**35**										0.119	0.860**	0.350	-0.294	0.671*	-0.294	-0.490	-0.098	-0.218	0.217	-0.531	-0.420	
**36**											-0.063	0.427	0.441	0.587*	0.098	0.280	0.287	-0.049	-0.203	-0.070	0.077	
**37**												0.462	-0.168	0.392	-0.182	-0.392	-0.119	-0.338	0.224	-0.301	-0.601*	
**38_39**													0.406	0.189	0.126	-0.070	-0.238	-0.641*	0.238	0.140	-0.594*	
**41**														0.014	0.217	0.573	0.210	0.056	-0.476	0.210	0.238	
**42**															-0.280	0.070	0.462	-0.007	-0.091	-0.385	-0.021	
**43**																0.133	-0.126	0.324	0.196	0.748**	0.266	
**44**																	0.790**	0.169	-0.636*	0.238	0.392	
**45**																		0.303	-0.524	-0.014	0.413	
**46**																			-0.444	0.127	0.916**	
**47**																				0.231	-0.538	
**51**																					0.133	

**V**	**53**	**54**	**55+56**	**57**	**58**	**59**	**60+61**	**62**	**63**	**64+65**	**66**	**67**	**68**	**69**	**70**	**71**	**72**	**73**	**74**	**75**	**76**	**77**
**1**	-0.077	-0.035	-0.317	-0.197	0.423	0.690*	0.120	-0.155	-0.092	0.092	-0.366	-0.049	-0.359	0.148	0.035	0.239	-0.028	0.775**	0.106	-0.148	0.208	-0.141
**2**	0.224	0.021	0.427	0.021	-0.622*	-0.517	-0.056	0.091	-0.141	0.077	0.182	0.133	0.196	-0.189	-0.056	-0.280	0.105	-0.420	-0.252	0.413	-0.306	0.077
**3**	-0.231	-0.510	0.182	-0.685*	0.196	0.063	-0.245	0.224	-0.380	0.084	-0.573	0.056	-0.301	0.462	-0.133	-0.084	0.406	-0.056	-0.357	-0.322	-0.324	0.273
**4**	-0.538	0.203	0.517	0.343	0.035	-0.035	0.280	0.098	0.246	-0.524	0.476	0.846**	0.441	-0.028	0.441	-0.825**	0.350	0.336	0.105	-0.455	0.093	-0.224
**5**	-0.280	-0.629*	0.517	-0.503	-0.049	-0.168	-0.399	0.294	-0.345	-0.042	-0.510	0.147	-0.070	0.476	-0.007	-0.329	0.490	-0.252	-0.371	-0.252	-0.381	-0.028
**6**	-0.028	0.112	0.140	-0.077	-0.671*	-0.441	0.098	0.322	-0.275	-0.182	0.266	0.531	-0.105	-0.545	-0.154	-0.483	-0.077	0.049	-0.357	0.308	-0.367	0.077
**7**	0.727**	-0.350	-0.594*	-0.294	0.021	-0.056	-0.434	-0.084	-0.134	0.713**	-0.294	-0.706*	-0.252	0.133	-0.559	0.825**	-0.322	-0.399	-0.035	0.413	-0.057	0.469
**8**	0.028	-0.112	0.021	0.427	0.322	-0.140	-0.189	-0.168	0.563	0.133	0.434	-0.098	0.685*	0.231	0.294	0.028	0.028	-0.441	0.469	-0.350	0.359	0.154
**9**	0.566	0.259	-0.455	0.503	0.231	0.350	0.028	-0.608*	0.535	0.483	0.147	-0.671*	0.329	0.063	0.147	0.650*	-0.238	-0.070	0.629*	0.147	0.623*	-0.035
**10+11**	-0.056	.734**	0.210	0.811**	-0.077	0.203	0.490	-0.573	0.570	-0.259	0.483	0.070	0.545	-0.322	0.643*	-0.273	-0.203	0.140	0.566	0.049	0.523	-0.503
**12**	-0.392	-0.476	0.671*	-0.441	0.007	-0.182	-0.245	0.357	-0.394	-0.231	-0.469	0.133	-0.126	0.483	0.007	-0.322	0.538	-0.231	-0.455	-0.203	-0.441	-0.175
**13**	-0.315	-0.531	0.098	-0.559	-0.014	0.098	-0.490	0.042	-0.408	-0.189	-0.839**	0.007	-0.413	0.280	-0.182	-0.210	-0.273	-0.014	-0.259	-0.077	-0.352	-0.161
**14**	-0.413	-0.462	0.517	-0.448	-0.378	-0.392	-0.350	0.483	-0.394	-0.322	-0.378	0.266	-0.133	0.245	-0.140	-0.503	0.490	-0.231	-0.503	-0.203	-0.513	-0.042
**15**	-0.741**	0.329	0.552	0.357	-0.056	-0.063	0.322	0.070	0.254	-0.734**	0.378	0.671*	0.392	-0.112	0.531	-0.853**	0.273	0.238	0.112	-0.490	0.082	-0.343
**16**	0.734**	-0.364	-0.636*	-0.385	-0.063	-0.133	-0.420	0.042	-0.254	0.706*	-0.294	-0.685*	-0.378	0.035	-0.657*	0.846**	-0.336	-0.392	-0.161	0.490	-0.164	0.510
**17**	0.699*	-0.371	-0.622*	-0.364	-0.035	-0.105	-0.448	-0.014	-0.204	0.692*	-0.322	-0.720**	-0.329	0.077	-0.608*	0.832**	-0.350	-0.413	-0.105	0.441	-0.125	0.497
**18**	-0.119	0.545	0.056	.832**	0.259	0.538	0.252	-0.797**	0.775**	-0.238	0.203	-0.056	0.622*	0.077	0.671*	-0.189	-0.189	0.280	.839**	-0.287	0.769**	-0.566
**19**	0.014	-0.608*	0.014	-0.259	0.399	0.196	-0.448	0.007	0.141	0.378	-0.203	-0.021	0.273	0.720**	-0.014	0.070	0.545	0.070	0.133	-0.531	0.128	0.399
**20**	0.783**	-0.196	-0.455	0.105	0.140	0.035	-0.343	-0.315	0.261	0.755**	0.021	-0.629*	0.217	0.273	-0.280	0.727**	-0.112	-0.364	0.301	0.238	0.267	0.406
**21**	0.322	0.434	-0.154	0.713**	0.266	0.371	0.182	-0.776**	0.796**	0.280	0.343	-0.329	0.692*	0.119	0.497	0.196	-0.049	-0.070	.818**	-0.168	0.772**	-0.070
**22**	-0.462	-0.448	0.469	-0.413	-0.133	-0.266	-0.287	0.566	-0.401	-0.413	-0.441	0.168	-0.259	0.399	-0.196	-0.322	0.622*	-0.147	-0.517	-0.301	-0.459	-0.203
**23**	-0.273	-0.203	0.217	0.000	0.133	0.014	-0.056	0.273	0.162	-0.126	0.154	0.441	0.273	0.413	0.070	-0.357	0.846**	0.238	0.000	-0.643*	0.085	0.133
**25**	0.014	-0.734**	-0.063	-0.790**	0.014	-0.224	-0.594*	0.238	-0.507	0.259	-0.587*	-0.070	-0.378	0.238	-0.434	0.063	-0.280	-0.371	-0.420	0.077	-0.534	0.483
**26**	0.021	-0.867**	-0.049	-0.636*	-0.105	-0.105	-0.804**	0.266	-0.387	0.154	-0.776**	-0.098	-0.315	0.524	-0.497	0.014	0.126	-0.196	-0.329	-0.126	-0.377	0.147

**27**	0.329	-0.259	0.014	-0.315	-0.189	-0.490	-0.238	0.154	-0.155	0.497	0.217	-0.210	0.140	-0.049	-0.245	0.224	-0.091	-0.531	-0.224	0.315	-0.324	0.776**
**28**	0.189	0.469	-0.217	0.510	-0.126	-0.273	0.329	0.133	0.296	0.042	0.853**	-0.182	0.294	-0.462	0.021	0.315	-0.077	0.000	0.161	0.252	0.189	0.175
**29**	-0.189	-0.545	0.238	-0.392	0.028	0.000	-0.462	0.238	-0.162	0.007	-0.483	-0.133	-0.035	.650*	-0.182	-0.028	0.629*	-0.021	-0.210	-0.357	-0.185	0.098
**30**	.608*	-0.434	-0.336	-0.378	0.098	-0.238	-0.378	0.140	-0.120	0.748**	-0.021	-0.469	-0.035	0.287	-0.503	0.657*	0.126	-0.455	-0.154	0.189	-0.157	0.797**
**32**	-0.329	-0.357	0.497	-0.371	-0.168	-0.210	-0.161	0.462	-0.352	-0.189	-0.252	0.140	-0.119	0.301	-0.063	-0.259	.643*	-0.014	-0.448	-0.168	-0.384	-0.056
**33_34**	-0.462	-0.399	0.538	-0.112	-0.280	-0.490	-0.413	0.399	-0.099	-0.510	-0.161	0.441	0.154	0.301	-0.077	-0.685*	0.392	-0.420	-0.280	-0.385	-0.356	-0.154
**35**	0.510	-0.587*	-0.441	-0.566	0.147	-0.189	-0.441	0.154	-0.232	0.755**	-0.189	-0.350	-0.189	0.189	-0.469	0.559	-0.084	-0.448	-0.189	0.126	-0.214	0.804**
**36**	-0.042	0.126	0.161	0.091	-0.084	-0.371	0.084	0.070	0.183	0.035	0.601*	0.336	0.434	-0.133	0.056	-0.287	0.035	-0.196	0.021	-0.077	-0.078	0.587*
**37**	0.378	-0.538	-0.280	-0.636*	0.154	-0.175	-0.294	0.231	-0.394	0.685*	-0.203	-0.392	-0.301	0.070	-0.343	0.573	-0.133	-0.364	-0.315	0.266	-0.320	0.664*
**38_39**	0.287	-0.217	0.329	-0.545	-0.273	-0.524	0.021	0.343	-0.570	0.385	0.084	0.154	-0.203	-0.182	-0.273	0.000	0.000	-0.434	-0.622*	0.545	-0.644*	0.559
**41**	0.140	0.301	0.336	0.441	-0.231	-0.427	0.210	0.105	0.225	-0.007	.678*	-0.007	0.476	-0.154	0.133	-0.028	0.252	-0.308	0.028	0.203	0.021	0.056
**42**	0.329	-0.469	-0.406	-0.147	0.084	-0.252	-0.510	0.196	0.190	0.483	0.210	-0.133	0.266	0.329	-0.420	0.301	0.238	-0.252	0.077	-0.252	0.032	0.811**
**43**	0.252	0.524	-0.063	0.357	0.091	0.364	0.573	-0.420	0.169	0.196	0.448	0.336	0.140	-0.462	0.364	-0.070	-0.364	0.538	0.287	0.329	0.310	-0.112
**44**	-0.350	0.063	0.343	0.503	0.133	-0.063	0.084	0.189	0.423	-0.350	0.566	0.448	0.580*	0.203	0.315	-0.413	0.587*	0.245	0.231	-0.510	0.260	-0.203
**45**	-0.210	-0.217	-0.021	0.378	0.189	0.063	-0.252	0.105	0.570	-0.063	0.378	0.189	0.636*	0.441	0.182	-0.189	0.692*	0.245	0.413	-0.755**	0.434	0.091
**46**	0.155	0.408	-0.246	0.845**	0.380	0.641*	0.155	-0.803**	.929**	0.120	0.296	-0.176	0.718**	0.218	0.577*	0.085	0.049	0.352	0.986**	-0.423	0.975**	-0.310
**47**	0.343	-0.070	-0.441	-0.559	-0.517	-0.112	-0.049	0.203	-0.620*	0.259	-0.357	-0.119	-0.748**	-0.510	-0.545	0.259	-0.448	0.119	-0.483	0.636*	-0.438	0.238
**51**	0.049	0.385	0.168	0.217	-0.035	0.399	0.399	-0.308	0.014	-0.028	0.091	0.231	0.014	-0.182	0.266	-0.147	-0.084	0.643*	0.119	0.259	0.164	-0.336
**52**	0.070	0.476	-0.084	0.937**	0.224	0.483	0.196	-0.657*	0.944**	-0.063	0.420	0.000	.790**	0.217	0.552	-0.112	0.287	0.322	0.902**	-0.490	0.915**	-0.378
**53**		-0.126	-0.587*	0.007	-0.273	-0.098	-0.238	-0.147	0.056	0.874**	0.077	-0.503	0.000	-0.070	-0.448	.706*	-0.042	-0.266	0.091	0.497	0.139	0.448
**54**			0.084	0.573	-0.007	0.301	0.867**	-0.434	0.303	-0.294	0.510	0.063	0.154	-0.566	0.538	-0.105	-0.245	0.413	0.322	0.196	0.367	-0.329
**55_56**				-0.042	0.161	-0.042	0.273	-0.028	-0.148	-0.503	-0.084	0.531	0.175	0.217	0.503	-0.734**	0.231	-0.105	-0.203	-0.168	-0.260	-0.385
**57**					0.112	0.322	0.273	-0.524	.880**	-0.189	0.573	-0.007	0.755**	0.014	0.538	-0.112	0.161	0.280	0.811**	-0.336	0.815**	-0.441
**58**						0.671*	0.224	-0.427	0.310	0.028	-0.035	0.203	0.259	0.490	0.531	-0.049	0.014	0.371	0.427	-0.517	0.409	-0.112
**59**							0.343	-0.776**	0.437	0.042	-0.210	0.056	0.196	0.315	0.608*	-0.028	-0.021	0.748**	0.650*	-0.378	0.691*	-0.434
**60_61**							1.000	-0.301	0.035	-0.238	0.441	0.308	-0.021	-0.538	0.601*	-0.196	-0.210	0.476	0.091	0.196	0.160	-0.238
**62**									-0.620*	-0.189	0.035	0.084	-0.490	-0.210	-0.671*	0.042	0.161	-0.343	-0.790**	0.182	-0.747**	0.322
**63**										0.035	0.444	-0.070	0.887**	0.317	0.535	-0.049	0.289	0.239	0.951**	-0.599*	0.928**	-0.197
**64_65**											0.014	-0.420	0.049	0.098	-0.287	.664*	0.014	-0.210	0.112	0.301	0.142	0.636*
**66**												0.203	0.510	-0.434	0.245	-0.028	0.063	0.077	0.280	-0.007	0.295	0.252
**67**													0.119	-0.049	0.357	-0.839**	0.077	0.336	-0.105	-0.301	-0.132	-0.161
**68**														0.385	0.566	-0.259	0.406	0.007	0.776**	-0.580*	0.712**	-0.063
**69**															0.091	-0.070	0.573	-0.063	0.308	-0.664*	0.278	-0.112
**70**																-0.503	0.056	0.413	0.601*	-0.434	0.573	-0.476
**71**																	-0.140	-0.175	0.007	0.420	0.082	0.406
**72**																		0.000	0.112	-0.559	0.210	-0.021
**73**																			0.371	-0.273	0.459	-0.371
**74**																				-0.531	0.975**	-0.266
**75**																					-0.513	0.210
**76**																						-0.288
**V**	**78**	**79**	**80**	**81**	**82**	**83**	**84**	**85**	**86**	**87**	**89**	**90**	**91**	**92**	**94**	**95**	**96**	**98**	**99**	**100**	**102**	**104**
**1**	-0.007	-0.127	0.169	0.148	-0.035	0.169	-0.120	-0.310	-0.282	0.127	0.063	-0.331	-0.028	0.092	-0.324	0.204	-0.183	0.720**	0.711**	0.602*	0.155	0.310
**2**	0.049	0.448	-0.385	-0.406	0.490	-0.385	0.056	0.217	0.042	-0.084	-0.035	0.357	-0.119	-0.042	0.210	0.035	0.434	-0.771**	-0.874**	-0.434	-0.007	-0.483
**3**	0.112	0.455	-0.371	-0.350	-0.182	-0.371	0.245	-0.126	-0.070	0.476	0.476	0.105	-0.035	0.329	-0.021	0.091	0.049	0.245	0.448	0.274	-0.154	-0.497
**4**	-0.077	0.196	0.084	0.007	0.189	0.084	-0.294	0.406	0.224	-0.112	0.063	0.455	0.622*	-0.455	0.371	0.084	0.245	-0.245	-0.126	-0.121	-0.175	0.112
**5**	0.483	0.671*	-0.420	-0.434	0.175	-0.420	-0.098	-0.182	-0.224	0.280	0.559	0.357	-0.119	0.049	-0.168	0.483	0.587*	-0.336	-0.098	0.160	-0.126	-0.434

**6**	-0.448	0.224	-0.441	-0.497	0.776**	-0.441	0.182	0.497	0.329	0.049	-0.399	0.490	0.196	-0.112	0.483	0.007	0.287	-0.137	-0.315	-0.502	-0.189	-0.259
**7**	-0.049	0.028	0.035	0.084	-0.315	0.035	0.517	-0.280	-0.119	0.329	0.000	-0.587*	-0.329	0.615*	-0.196	-0.427	-0.483	0.165	0.042	0.050	0.308	-0.161
**8**	0.238	-0.133	0.490	0.538	-0.587*	0.490	0.035	0.056	0.091	0.091	0.259	0.042	0.175	0.112	0.000	-0.196	-0.147	-0.445	-0.182	-0.089	-0.063	0.196
**9**	0.217	-0.469	0.643*	0.699*	-0.476	.643*	-0.063	-0.413	-0.308	-0.238	-0.056	-0.552	-0.427	0.259	-0.399	-0.105	-0.413	-0.049	-0.140	0.295	0.308	0.420
**10+11**	0.077	-0.497	0.427	0.441	0.105	0.427	-0.497	-0.056	-0.196	-0.699*	-0.168	0.063	-0.189	-0.378	-0.182	0.252	0.168	-0.385	-0.497	-0.018	-0.182	0.385
**12**	0.552	0.629*	-0.462	-0.490	0.126	-0.462	-0.294	-0.140	-0.175	0.063	0.517	0.336	-0.049	-0.231	-0.140	0.448	0.650*	-0.420	-0.189	0.093	-0.007	-0.392
**13**	0.140	0.308	-0.392	-0.371	0.364	-0.392	0.000	-0.476	-0.545	0.238	0.420	-0.203	-0.133	0.042	-0.580*	0.336	0.245	0.294	0.273	0.274	-0.490	-0.350
**14**	0.322	.629*	-0.594*	-0.615*	0.441	-0.594*	-0.070	0.021	-0.042	0.245	0.252	0.462	0.063	-0.182	0.070	0.364	0.538	-0.277	-0.161	-0.085	-0.189	-0.510
**15**	0.014	-0.049	0.000	-0.021	0.217	0.000	-0.385	0.322	0.161	-0.224	0.007	0.545	0.441	-0.573	0.245	0.238	0.322	-0.200	-0.070	-0.146	-0.420	0.070
**16**	-0.133	0.028	-0.070	-0.028	-0.224	-0.070	0.573	-0.189	-0.014	0.364	-0.105	-0.510	-0.322	0.615*	-0.098	-0.434	-0.455	0.224	0.084	-0.036	0.329	-0.182
**17**	-0.084	0.007	-0.042	0.014	-0.259	-0.042	0.559	-0.245	-0.077	0.371	-0.049	-0.531	-0.343	0.622*	-0.168	-0.413	-0.462	0.214	0.091	0.004	0.266	-0.196
**18**	0.266	-0.483	0.699*	0.727**	-0.175	0.699*	-0.545	-0.448	-0.538	-0.573	0.112	-0.378	-0.126	-0.294	-0.538	0.224	-0.126	-0.102	-0.189	0.406	-0.196	0.503
**19**	0.364	0.566	0.147	0.154	-0.441	0.147	0.238	-0.182	-0.175	0.699*	0.587*	-0.140	0.273	0.420	-0.077	-0.133	-0.245	0.004	0.280	0.448	0.196	-0.231
**20**	0.126	0.063	0.364	0.399	-0.510	0.364	0.357	-0.280	-0.168	0.210	0.105	-0.615*	-0.210	0.545	-0.175	-0.476	-0.538	-0.151	-0.224	0.132	0.455	-0.014
**21**	0.175	-0.427	0.734**	0.797**	-0.559	.734**	-0.112	-0.308	-0.287	-0.308	0.105	-0.364	-0.273	0.203	-0.273	-0.105	-0.399	-0.207	-0.231	0.295	0.021	0.315
**22**	0.455	0.531	-0.462	-0.510	0.203	-0.462	-0.280	-0.070	-0.007	0.077	0.231	0.294	0.098	-0.364	0.035	0.371	0.469	-0.137	0.007	0.046	0.091	-0.238
**23**	0.168	0.448	0.084	0.021	-0.196	0.084	-0.021	0.196	0.231	0.322	0.168	0.224	0.503	-0.056	0.413	-0.056	-0.112	0.014	0.203	0.192	0.259	-0.014
**25**	-0.140	0.455	-0.490	-0.441	0.042	-0.490	0.580*	-0.091	-0.119	0.678*	0.420	-0.084	0.021	0.503	-0.154	-0.189	-0.021	0.175	0.266	-0.057	-0.385	-0.657*
**26**	0.287	.678*	-0.350	-0.364	0.224	-0.350	0.217	-0.427	-0.399	0.559	0.420	-0.266	-0.021	0.273	-0.350	0.126	0.063	0.154	0.161	0.278	-0.028	-0.406
**27**	-0.161	0.322	-0.329	-0.252	-0.147	-0.329	0.685*	0.385	0.287	0.601*	0.119	0.210	0.112	0.497	0.329	-0.559	-0.133	-0.431	-0.308	-0.466	-0.070	-0.706*
**28**	-0.126	-0.434	0.238	0.252	-0.133	0.238	0.028	0.545	0.552	-0.098	-0.580*	0.210	0.280	-0.231	0.483	-0.406	-0.203	-0.277	-0.266	-0.495	0.378	0.322
**29**	0.566	0.629*	-0.245	-0.245	-0.049	-0.245	-0.035	-0.287	-0.273	0.441	0.434	-0.056	0.098	0.014	-0.168	0.126	0.077	-0.077	0.077	0.342	0.196	-0.385
**30**	-0.049	0.336	-0.063	-0.021	-0.538	-0.063	0.692*	0.091	0.217	0.622*	0.140	-0.266	0.028	0.671*	0.238	-0.671*	-0.510	-0.070	0.007	-0.100	0.406	-0.427
**32**	0.455	0.573	-0.455	-0.490	0.259	-0.455	-0.203	0.049	0.007	0.196	0.231	0.441	0.070	-0.231	0.091	0.364	0.524	-0.312	-0.126	0.028	0.154	-0.329
**33_34**	0.259	0.545	-0.322	-0.364	0.259	-0.322	-0.147	0.042	0.007	0.056	0.266	0.294	0.308	-0.343	0.112	0.161	0.371	-0.378	-0.294	-0.192	-0.259	-0.308
**35**	-0.217	0.231	-0.112	-0.056	-0.448	-0.112	0.804**	0.063	0.203	0.727**	0.196	-0.140	-0.091	0.860**	0.154	-0.476	-0.413	0.175	0.301	-0.057	0.084	-0.392
**36**	-0.406	0.175	-0.084	-0.049	-0.189	-0.084	0.510	0.622*	0.469	0.392	0.000	0.266	0.594*	0.154	0.608*	-0.706*	-0.322	-0.287	-0.196	-0.491	-0.224	-0.476
**37**	-0.091	0.161	-0.252	-0.189	-0.294	-0.252	.615*	0.112	0.196	0.629*	0.196	0.112	-0.217	0.706*	0.070	-0.203	-0.056	0.039	0.252	-0.107	0.063	-0.364
**38_39**	-0.308	0.538	-0.636*	-0.643*	0.168	-0.636*	0.503	0.510	0.385	0.308	0.105	0.448	0.000	0.343	0.469	-0.294	0.308	-0.501	-0.434	-0.552	0.007	-0.748**
**41**	0.175	0.070	0.042	0.028	-0.049	0.042	-0.140	0.420	0.343	-0.224	-0.182	0.336	0.175	-0.301	0.399	-0.210	0.182	-0.820**	-0.762**	-0.477	0.336	-0.028
**42**	-0.119	0.350	0.133	0.161	-0.490	0.133	0.720**	0.217	0.301	.839**	0.084	-0.231	0.510	0.545	0.392	-0.790**	-0.734**	0.091	0.196	-0.082	0.252	-0.287
**43**	-0.441	-0.252	0.280	0.231	0.203	0.280	-0.070	0.210	0.028	-0.301	-0.203	0.028	-0.007	0.056	0.077	0.007	0.028	0.004	-0.133	-0.004	0.028	0.308
**44**	0.329	0.189	0.322	0.245	-0.077	0.322	-0.413	0.287	0.224	-0.049	0.021	0.294	0.629*	-0.510	0.308	0.035	0.126	-0.399	-0.203	-0.025	0.315	0.364
**45**	0.406	0.224	0.462	0.434	-0.287	0.462	-0.105	0.070	0.091	0.399	0.119	0.077	0.580*	-0.098	0.196	-0.070	-0.259	-0.091	0.140	0.256	0.308	0.294
**46**	0.282	-0.444	0.937**	0.958**	-0.444	0.937**	-0.345	-0.451	-0.437	-0.310	0.085	-0.479	-0.106	0.028	-0.408	0.085	-0.430	0.046	0.007	0.581*	0.113	0.641*
**47**	-0.531	-0.007	-0.524	-0.524	0.629*	-0.524	0.455	0.063	0.063	0.217	-0.434	-0.021	-0.329	0.350	0.077	-0.007	-0.021	0.511	0.210	-0.167	-0.084	-0.287
**51**	-0.035	0.049	0.056	-0.007	0.427	0.056	-0.343	-0.056	-0.273	-0.308	-0.070	-0.070	-0.014	-0.238	-0.161	0.224	0.217	-0.063	-0.231	0.192	0.154	0.140
**52**	0.308	-0.315	0.874**	0.860**	-0.350	0.874**	-0.441	-0.322	-0.308	-0.406	-0.007	-0.364	0.042	-0.175	-0.203	0.063	-0.371	-0.091	-0.161	0.466	0.210	0.622*
**53**	-0.105	0.105	0.175	0.168	-0.091	0.175	0.469	-0.105	0.021	0.196	-0.252	-0.371	-0.392	.657*	0.091	-0.322	-0.406	-0.067	-0.287	0.004	0.573	-0.007
**54**	-0.287	-0.713**	0.224	0.252	0.028	0.224	-0.322	0.210	0.112	-0.664*	-0.420	0.133	-0.133	-0.357	0.105	0.035	-0.021	0.028	-0.112	-0.110	-0.147	0.294
**55_56**	0.238	0.364	-0.301	-0.329	0.091	-0.301	-0.469	0.063	-0.147	-0.385	0.552	0.420	0.091	-0.462	-0.084	0.350	.713**	-0.613*	-0.420	-0.075	-0.378	-0.343
**57**	0.287	-0.427	0.790**	0.776**	-0.210	0.790**	-0.517	-0.161	-0.175	-0.503	-0.175	-0.210	0.091	-0.371	-0.126	0.077	-0.224	-0.217	-0.287	0.238	0.210	.678*
**58**	0.091	-0.098	0.510	0.510	-0.734**	0.510	-0.210	-0.203	-0.224	-0.035	0.629*	-0.273	0.231	0.070	-0.308	-0.028	-0.168	0.182	0.469	0.516	-0.091	0.287
**59**	0.147	-0.273	0.615*	0.608*	-0.315	0.615*	-0.420	-0.566	-0.615*	-0.301	0.371	-0.483	-0.182	0.049	-0.601*	0.329	-0.182	0.413	0.413	0.847**	-0.049	0.462
**60_61**	-0.420	-0.566	0.056	0.063	-0.035	0.056	-0.259	0.378	0.252	-0.573	-0.217	0.364	-0.084	-0.252	0.217	0.098	0.189	0.046	0.056	-0.132	-0.217	0.182
**62**	-0.133	0.287	-0.636*	-0.671*	0.322	-0.636*	0.287	0.559	0.664*	0.399	-0.371	0.483	0.364	-0.182	0.601*	-0.189	0.203	-0.014	0.063	-0.634*	0.189	-0.245

**63**	0.359	-0.275	0.908**	0.923**	-0.486	0.908**	-0.296	-0.296	-0.282	-0.155	0.099	-0.345	0.141	-0.042	-0.204	-0.035	-0.458	-0.106	-0.070	0.462	0.162	0.556
**64_65**	-0.112	0.189	0.175	0.196	-0.322	0.175	0.608*	-0.070	0.021	0.462	0.056	-0.252	-0.329	0.888**	0.070	-0.322	-0.399	-0.014	-0.035	0.128	0.406	-0.147
**66**	-0.308	-0.350	0.329	0.329	-0.210	0.329	0.119	0.685*	0.643*	-0.035	-0.448	0.371	0.434	-0.105	0.664*	-0.434	-0.245	-0.263	-0.189	-0.459	0.175	0.273
**67**	-0.364	0.280	-0.091	-0.182	0.203	-0.091	-0.133	0.385	0.189	-0.084	0.238	0.385	0.538	-0.245	0.301	0.070	0.308	-0.042	0.063	-0.103	-0.364	-0.035
**68**	0.385	0.035	0.706*	0.713**	-0.455	0.706*	-0.217	-0.112	-0.182	-0.049	0.301	-0.091	0.252	-0.021	-0.056	-0.077	-0.231	-0.473	-0.329	0.263	0.077	0.259
**69**	0.692*	0.573	0.350	0.322	-0.490	0.350	-0.231	-0.615*	-0.559	0.203	0.769**	-0.490	0.133	0.077	-0.469	0.091	-0.133	-0.147	0.028	0.669*	0.231	0.000
**70**	0.161	-0.329	0.469	0.490	-0.273	0.469	-0.545	-0.133	-0.308	-0.497	0.406	0.175	-0.063	-0.231	-0.301	0.413	0.252	-0.203	-0.014	0.374	-0.406	0.287
**71**	0.028	-0.252	0.133	0.182	-0.301	0.133	0.371	-0.119	0.105	0.245	-0.350	-0.385	-0.350	0.448	-0.014	-0.322	-0.476	0.228	0.133	0.018	0.573	0.133
**72**	0.580*	0.483	0.196	0.140	-0.231	0.196	-0.224	-0.098	0.000	0.140	0.203	0.098	0.168	-0.084	0.175	0.140	0.000	-0.294	-0.140	0.310	0.510	0.070
**73**	-0.133	-0.287	0.378	0.329	0.077	0.378	-0.357	-0.105	-0.196	-0.189	-0.077	-0.168	0.196	-0.217	-0.154	0.217	-0.140	0.553	0.497	0.502	0.035	0.503
**74**	0.308	-0.350	0.937**	0.958**	-0.469	0.937**	-0.315	-0.448	-0.455	-0.203	0.182	-0.462	-0.014	0.056	-0.406	0.070	-0.441	0.042	0.049	0.619*	0.070	0.587*
**75**	-0.441	-0.126	-0.538	-0.524	0.455	-0.538	0.315	0.273	0.245	-0.147	-0.476	0.126	-0.434	0.154	0.182	-0.140	0.203	-0.105	-0.357	-0.555	0.070	-0.315
**76**	0.317	-0.381	0.954**	0.961**	-0.459	0.954**	-0.352	-0.434	-0.402	-0.238	0.085	-0.441	-0.061	0.046	-0.349	0.117	-0.445	0.105	0.096	0.659*	0.210	0.676*
**77**	-0.455	0.217	-0.238	-0.182	-0.294	-0.238	0.951**	0.517	0.566	0.818**	-0.105	0.140	0.231	0.727**	0.601*	-0.755**	-0.490	0.056	0.154	-0.416	0.091	-0.531
**78**		0.252	0.301	0.294	-0.154	0.301	-0.601*	-0.650*	-0.573	-0.161	0.385	-0.238	-0.203	-0.301	-0.559	0.490	0.217	-0.403	-0.294	0.516	0.336	0.231
**79**			-0.315	-0.392	0.119	-0.315	0.161	-0.091	-0.147	0.413	0.510	-0.042	0.231	0.175	0.049	-0.063	0.182	-0.375	-0.315	0.068	0.203	-0.510
**80**				0.986**	-0.559	1.000**	-0.315	-0.385	-0.315	-0.182	0.119	-0.497	0.077	0.049	-0.294	0.007	-0.490	0.091	0.119	0.605*	0.308	0.748**
**81**					-0.608*	0.986**	-0.259	-0.385	-0.315	-0.147	0.119	-0.483	0.028	0.098	-0.315	-0.021	-0.524	0.102	0.147	0.584*	0.231	0.692*
**82**						-0.559	-0.126	0.077	-0.035	-0.175	-0.406	0.287	-0.168	-0.315	0.014	0.399	0.517	0.004	-0.266	-0.295	-0.168	-0.140
**83**							-0.315	-0.385	-0.315	-0.182	0.119	-0.497	0.077	0.049	-0.294	0.007	-0.490	0.091	0.119	0.605*	0.308	0.748**
**84**								0.455	0.497	0.776**	-0.168	0.077	0.133	0.769**	0.524	-0.706*	-0.483	0.210	0.210	-0.424	-0.056	-0.552
**85**									0.937**	0.245	-0.441	0.706*	0.490	-0.021	0.916**	-0.497	0.028	-0.112	0.007	-0.826**	-0.084	-0.245
**86**										0.294	-0.552	0.615*	0.420	0.042	0.944**	-0.524	-0.105	-0.004	0.105	-0.787**	0.112	-0.119
**87**											0.091	0.028	0.378	0.608*	0.336	-0.510	-0.434	0.217	0.371	-0.110	0.063	-0.420
**89**												-0.189	0.021	0.168	-0.503	0.196	0.203	-0.263	0.000	0.505	-0.287	-0.273
**90**													0.070	-0.147	0.573	0.189	0.587*	-0.329	-0.105	-0.605*	-0.322	-0.259
**91**														-0.273	0.476	-0.545	-0.301	0.060	0.210	-0.238	0.035	0.021
**92**															0.091	-0.329	-0.420	0.182	0.210	0.117	0.028	-0.329
**94**																-0.566	-0.161	-0.074	-0.014	-0.719**	0.175	-0.210
**95**																	0.713**	-0.088	-0.070	0.431	-0.175	0.301
**96**																		-0.480	-0.378	-0.182	-0.301	-0.154
**98**																			0.876**	0.298	-0.095	0.259
**99**																				0.303	-0.189	0.217
**100**																					0.146	0.399
**102**																						0.378

P: Peak (numbered according to Table 2); *p*-Value corresponds to the significance level of Spearman correlation coefficient indicated as *: significant at $p < 0.05$; **: significant at $p < 0.01$.

Table S7: Spearman correlation coefficients among the volatile compounds from traditional maize flours and *broas*.

B \ F	5	6	7	12	14	17	19	20	22	23	24	26	30	33+34	35	42	45	49	50	64+65	67	71	92	94	95	96	98	99	102
**1**	0.04	0.10	0.15	0.00	0.06	0.20	0.01	-0.40	0.07	0.24	0.48	-0.04	0.10	0.11	0.15	0.30	0.08	0.26	-0.60*	0.35	0.23	0.25	0.23	-0.21	-0.11	-0.15	.674*	0.75**	0.44
**2**	-0.27	0.41	-0.39	-0.06	-0.11	-0.48	-0.36	0.13	-0.29	-0.34	-0.64*	-0.03	-0.34	-0.24	-0.31	-0.36	-0.13	-0.50	0.29	-0.34	-0.14	-0.18	0.04	0.01	0.26	0.40	-0.62*	-0.64*	-0.41
**3**	0.31	-0.22	0.42	0.29	0.29	0.50	0.37	0.39	0.45	0.57	0.56	0.19	0.53	0.65*	0.48	0.42	0.42	0.62*	-0.06	0.47	0.27	0.64*	0.58*	0.31	-0.54	-0.24	0.36	0.52	0.45
**4**	0.10	0.33	-0.25	0.45	-0.19	-0.41	-0.30	0.31	0.12	-0.26	0.20	-0.20	-0.25	0.36	-0.27	-0.21	0.37	-0.24	0.05	-0.15	0.71**	0.29	0.09	0.20	-0.12	-0.21	-0.21	0.03	-0.08
**5**	0.11	0.12	0.13	0.31	0.29	0.17	0.29	0.48	0.34	0.27	0.11	0.18	0.22	0.46	0.17	0.28	0.29	0.34	0.02	0.36	0.32	0.48	0.52	0.11	-0.68*	-0.28	-0.10	0.19	0.29
**6**	-0.19	0.22	-0.41	0.09	-0.33	-0.49	-0.60*	-0.04	-0.22	-0.45	-0.18	-0.40	-0.35	0.13	-0.39	-0.41	0.06	-0.40	-0.22	-0.38	0.27	0.11	0.22	0.22	0.31	0.23	0.00	-0.12	-0.23
**7**	-0.06	-0.36	0.34	-0.45	0.08	0.42	0.27	-0.13	-0.12	0.25	-0.21	0.26	0.26	-0.39	0.33	0.27	-0.29	0.16	0.04	0.22	-0.72**	-0.14	0.03	-0.08	0.13	0.33	0.13	-0.06	0.19
**8**	0.42	-0.10	0.43	0.37	0.38	0.34	0.55	0.48	0.44	0.25	-0.01	0.37	0.36	-0.12	0.36	0.32	0.00	0.24	0.33	0.29	0.01	-0.15	-0.21	0.27	-0.22	-0.10	-0.43	-0.25	0.19
**9**	-0.01	-0.01	0.20	-0.24	0.12	0.15	0.30	-0.39	-0.03	0.12	-0.34	0.28	0.08	-0.62*	0.16	0.13	-0.34	-0.06	0.09	0.08	-0.63*	-0.55	-0.48	-0.27	0.27	0.19	-0.15	-0.35	0.01
**10+11**	-0.22	0.27	-0.42	0.06	-0.15	-0.57	-0.26	-0.39	-0.20	-0.45	-0.49	-0.26	-0.43	-0.52	-0.44	-0.50	-0.36	-0.57	0.07	-0.51	-0.14	-0.66*	-0.74**	-0.14	0.38	-0.03	-0.46	-0.59*	-0.52
**12**	-0.03	0.22	-0.07	0.10	0.21	0.02	0.17	0.48	0.10	0.14	0.03	0.20	0.02	0.38	0.00	0.14	0.25	0.18	0.29	0.20	0.28	0.35	0.38	-0.13	-0.58*	-0.23	-0.29	0.06	0.03
**13**	-0.24	-0.48	-0.15	-0.07	-0.11	-0.03	-0.15	-0.13	-0.09	-0.17	-0.03	-0.50	-0.06	-0.01	-0.13	-0.14	-0.40	0.05	-0.53	-0.08	-0.05	0.13	0.06	0.00	-0.49	-0.52	0.40	0.37	0.10
**14**	0.08	0.10	-0.15	0.18	0.01	-0.09	-0.10	0.39	0.13	-0.03	0.03	-0.01	0.02	0.53	-0.06	-0.01	0.24	0.12	0.03	0.04	0.36	0.40	0.50	0.06	-0.46	-0.20	-0.03	0.06	0.04
**15**	0.18	0.20	-0.29	0.48	-0.10	-0.38	-0.28	0.22	0.15	-0.27	0.18	-0.27	-0.18	0.36	-0.27	-0.29	0.19	-0.16	0.01	-0.27	0.66*	0.06	-0.09	0.19	-0.11	-0.34	-0.15	-0.03	-0.22
**16**	-0.07	-0.35	0.33	-0.47	0.08	0.43	0.24	-0.13	-0.14	0.25	-0.17	0.25	0.26	-0.32	0.32	0.27	-0.24	0.19	0.01	0.22	-0.68*	-0.09	0.11	-0.07	0.17	0.38	0.20	-0.01	0.20
**17**	-0.03	-0.38	0.35	-0.43	0.11	0.45	0.27	-0.13	-0.10	0.27	-0.17	0.24	0.29	-0.35	0.35	0.28	-0.29	0.21	-0.01	0.22	-0.69*	-0.13	0.07	-0.06	0.14	0.34	0.20	-0.01	0.20
**18**	-0.15	-0.03	-0.29	0.01	-0.22	-0.42	-0.14	-0.48	-0.10	-0.37	-0.35	-0.30	-0.32	-0.53	-0.32	-0.38	-0.44	-0.44	-0.09	-0.37	-0.24	-0.57	-0.79**	-0.23	0.09	-0.34	-0.21	-0.38	-0.30
**19**	0.57	0.14	.587*	0.39	0.40	0.55	0.43	0.48	0.55	0.63*	0.47	0.52	0.58*	0.38	0.65*	0.72**	0.38	0.55	-0.07	0.78**	0.33	0.57	0.66*	0.16	-0.51	-0.03	0.18	0.47	0.74**
**20**	0.02	-0.11	0.33	-0.34	0.09	0.30	0.31	-0.04	-0.06	0.24	-0.32	0.43	0.20	-0.50	0.32	0.28	-0.21	0.01	0.26	0.24	-0.69*	-0.22	-0.08	-0.13	0.17	0.39	-0.19	-0.31	0.16
**21**	0.14	-0.06	0.19	0.10	0.07	0.03	0.29	-0.22	0.19	0.10	-0.24	0.17	0.13	-0.42	0.16	-0.01	-0.18	-0.10	0.19	-0.03	-0.42	-0.45	-0.57	0.03	0.18	0.00	-0.28	-0.48	-0.06
**22**	0.01	0.06	-0.14	-0.04	-0.05	-0.01	0.04	0.39	0.06	0.03	0.15	0.13	-0.03	.629*	-0.08	0.07	0.40	0.20	0.26	0.10	0.31	0.46	0.45	-0.21	-0.57	-0.30	-0.02	0.13	0.03
**23**	0.48	0.24	0.24	0.37	-0.01	0.15	0.13	0.48	0.45	0.32	0.56	0.35	0.27	0.82**	0.27	0.36	0.80**	0.32	0.18	0.41	0.61*	0.72**	0.64*	0.13	-0.38	-0.10	0.12	0.29	0.42
**25**	0.08	-0.50	0.31	0.09	0.22	0.42	0.15	0.31	0.14	0.24	0.15	-0.12	0.37	0.06	0.34	0.23	-0.21	0.36	-0.30	0.26	-0.05	0.30	0.36	0.39	-0.36	-0.12	0.30	0.41	0.34
**26**	-0.05	-0.34	0.08	-0.13	-0.10	0.17	0.03	0.13	0.05	0.06	0.01	-0.08	0.13	0.22	0.10	0.19	-0.04	0.21	-0.31	0.25	-0.08	0.45	0.50	-0.05	-0.59*	-0.28	0.35	0.33	0.39
**27**	0.36	0.19	0.36	0.20	0.50	0.36	0.10	0.48	0.10	0.34	-0.09	0.40	0.41	-0.22	0.48	0.31	-0.18	0.17	0.18	0.28	-0.06	-0.10	0.31	0.38	0.28	0.63*	-0.28	-0.13	0.10
**28**	0.25	0.57	-0.01	-0.06	0.21	-0.02	-0.12	0.04	-0.17	-0.05	-0.13	0.41	-0.06	-0.34	0.03	0.10	-0.09	-0.16	0.29	-0.01	0.06	-0.50	-0.13	-0.19	0.66*	0.66*	-0.34	-0.30	-0.20
**29**	0.27	0.20	0.12	0.02	0.16	0.20	0.08	0.31	0.16	0.29	0.17	0.36	0.23	0.37	0.27	0.36	0.20	0.29	0.05	0.41	0.18	0.38	0.59*	-0.22	-0.46	-0.06	0.11	0.24	0.32
**30**	0.33	-0.07	0.58*	-0.12	0.34	0.62*	0.41	0.39	0.16	0.58*	0.10	0.64*	0.54	-0.02	0.64*	0.56	0.13	0.39	0.34	0.52	-0.32	0.18	0.43	0.15	0.08	0.54	-0.04	0.02	0.36
**32**	0.15	0.50	-0.06	0.15	0.22	0.01	0.01	0.39	0.12	0.17	0.13	0.31	0.06	0.47	0.06	0.24	0.36	0.20	0.14	0.28	0.45	0.37	0.59*	-0.17	-0.35	0.03	-0.11	0.16	0.13
**33+34**	-0.04	-0.26	-0.27	0.08	-0.31	-0.27	-0.12	0.48	0.05	-0.31	-0.11	-0.21	-0.17	0.45	-0.27	-0.27	0.20	-0.11	0.29	-0.24	0.21	0.33	0.15	0.10	-0.54	-0.45	-0.25	-0.23	-0.15
**35**	0.38	-0.37	0.78**	0.15	0.45	0.82**	0.61*	0.39	0.44	0.70*	0.33	0.44	0.74**	0.16	0.76**	0.65*	0.18	.657*	-0.01	.063*	-0.18	0.36	0.48	0.48	-0.15	0.25	0.25	0.31	.601*
**36**	0.40	0.10	0.14	0.35	0.12	0.03	-0.19	0.48	0.10	0.07	0.12	0.09	0.20	0.02	0.23	-0.01	0.03	-0.07	0.24	-0.03	0.26	0.01	0.13	0.51	0.34	0.38	-0.24	-0.15	-0.07
**37**	0.36	-0.03	0.73**	0.24	0.73**	0.82**	0.63*	0.39	0.41	0.74**	0.31	0.52	0.706*	0.06	0.74**	0.73**	0.09	0.70*	-0.09	0.71**	-0.01	0.22	0.49	0.37	-0.11	0.34	0.15	0.41	0.57
**38+39**	-0.08	0.31	0.08	0.10	0.30	0.08	-0.01	0.48	-0.08	0.20	-0.12	0.22	0.10	-0.03	0.15	0.08	0.11	-0.01	0.31	0.10	0.06	0.17	0.40	0.36	0.23	.587*	-0.42	-0.12	-0.08
**41**	0.04	.657*	-0.20	-0.04	0.13	-0.27	-0.12	0.31	-0.21	-0.15	-0.43	0.41	-0.22	-0.28	-0.13	-0.08	0.01	-0.36	0.67*	-0.13	0.00	-0.37	-0.10	-0.19	0.41	0.55	-0.82**	-0.66*	-0.40
**42**	.615*	-0.17	.587*	0.13	0.16	0.55	0.23	0.48	0.34	0.45	0.33	0.48	0.57	0.23	.636*	0.55	0.24	0.39	0.10	0.52	0.03	0.36	0.55	0.31	-0.04	0.32	0.20	0.18	0.54
**43**	-0.29	0.43	-0.18	0.13	-0.08	-0.36	-0.29	-0.39	-0.19	-0.20	-0.11	-0.25	-0.33	-0.42	-0.25	-0.21	-0.08	-0.41	-0.30	-0.16	0.11	-0.17	-0.24	0.08	0.50	0.27	-0.10	0.01	-0.10
**44**	0.29	.587*	-0.10	0.27	-0.01	-0.20	-0.07	0.39	0.14	-0.09	0.13	0.29	-0.15	0.27	-0.10	0.13	0.41	-0.10	0.28	0.15	.601*	0.18	0.20	-0.18	-0.15	-0.01	-0.36	-0.06	0.08
**45**	.685*	0.36	0.32	0.43	0.15	0.21	0.18	0.39	0.51	0.27	0.40	0.48	0.31	0.47	0.35	0.51	0.48	0.30	0.00	0.53	0.57	0.37	0.48	-0.02	-0.34	-0.07	0.08	0.22	0.55
**46**	0.15	0.03	0.11	0.13	-0.08	-0.08	0.16	-0.40	0.21	0.00	-0.08	0.06	0.01	-0.30	0.05	0.03	-0.10	-0.12	-0.11	0.04	-0.19	-0.30	-0.48	-0.15	0.03	-0.21	-0.02	-0.20	0.12
**47**	-0.29	-0.15	-0.13	-0.27	-0.20	-0.05	-0.37	-0.48	-0.29	-0.13	-0.06	-0.36	-0.08	-0.05	-0.10	-0.16	-0.19	-0.06	-0.58*	-0.16	-0.21	0.06	0.24	0.08	0.35	0.28	0.58	0.27	0.01
**51**	-0.41	0.64*	-0.50	-0.13	-0.21	-0.58*	-0.57	-0.48	-0.45	-0.37	-0.27	-0.25	-0.55	-0.41	-0.45	-0.31	-0.20	-0.57	-0.29	-0.24	0.10	-0.19	-0.10	-0.36	0.37	0.23	-0.09	0.00	-0.23
**52**	0.11	0.11	-0.08	0.06	-0.29	-0.28	0.01	-0.31	0.13	-0.15	-0.14	0.05	-0.15	-0.13	-0.12	-0.13	0.08	-0.27	0.11	-0.11	-0.12	-0.22	-0.43	-0.22	0.04	-0.20	-0.15	-0.38	-0.03
**53**	-0.08	0.05	0.25	-0.33	-0.06	0.17	0.17	-0.22	-0.06	0.18	-0.34	0.36	0.13	-0.27	0.22	0.22	0.05	-0.03	0.08	0.20	-0.62*	-0.03	0.17	-0.06	0.34	0.58*	-0.02	-0.30	0.20
**54**	-0.14	0.31	-0.35	0.00	-0.08	-0.41	-0.35	-0.48	-0.27	-0.26	-0.12	-0.22	-0.31	-0.35	-0.31	-0.45	-0.22	-0.41	0.05	-0.49	-0.01	-0.58*	-0.62*	-0.09	0.69*	0.20	-0.15	-0.29	-0.56

**55+56**	-0.27	0.22	-0.37	0.23	0.05	-0.39	-0.10	0.39	-0.09	-0.19	-0.13	-0.20	-0.31	0.05	-0.34	-0.35	0.03	-0.27	0.42	-0.28	0.31	0.04	-0.20	0.07	-0.29	-0.31	-0.65*	-0.22	-0.43
**57**	0.06	0.27	-0.22	0.01	-0.24	-0.38	-0.11	-0.31	-0.03	-0.30	-0.27	0.03	-0.29	-0.28	-0.25	-0.21	-0.06	-0.39	0.15	-0.22	-0.08	-0.42	-0.52	-0.31	0.20	-0.08	-0.29	-0.47	-0.20
**58**	0.16	-0.11	0.39	0.35	0.31	0.34	0.48	0.22	0.36	0.41	0.51	0.10	0.26	0.01	0.31	0.34	0.16	0.31	0.02	0.40	0.28	0.22	-0.17	0.16	-0.39	-0.41	-0.01	0.45	0.34
**59**	-0.08	0.01	0.06	0.15	-0.03	-0.04	0.11	-0.48	0.15	0.13	0.24	-0.18	-0.02	-0.14	0.01	0.04	-0.01	0.01	-0.40	0.13	0.04	0.03	-0.31	-0.12	-0.17	-0.42	0.28	0.34	0.22
**60+61**	-0.12	0.38	-0.16	0.24	0.16	-0.22	-0.14	-0.22	-0.08	0.00	0.18	-0.16	-0.16	-0.13	-0.15	-0.25	0.04	-0.17	0.05	-0.26	0.28	-0.27	-0.41	0.15	0.52	0.15	-0.16	0.01	-0.38
**62**	0.15	0.06	0.01	-0.13	0.06	0.19	-0.07	0.48	-0.07	0.01	0.18	0.21	0.07	0.47	0.04	0.18	0.28	0.24	0.18	0.12	0.31	0.31	0.59*	-0.01	-0.05	0.25	0.08	0.18	0.06
**63**	0.35	0.10	0.15	0.23	-0.07	-0.05	0.16	-0.13	0.31	0.03	-0.02	0.20	0.08	-0.11	0.12	0.11	0.06	-0.07	0.04	0.11	-0.03	-0.18	-0.30	-0.10	-0.05	-0.18	-0.11	-0.25	0.17
**64+65**	0.21	0.06	0.62*	0.06	0.32	0.52	0.47	0.04	0.31	0.57	0.02	0.52	0.50	-0.10	0.59*	0.56	0.19	0.34	-0.04	0.57	-0.34	0.20	0.38	0.24	0.13	0.50	0.08	0.04	0.53
**66**	0.40	0.47	0.13	0.31	0.18	0.00	-0.03	0.22	0.13	0.06	0.11	0.31	0.08	-0.03	0.13	0.10	0.21	-0.07	0.26	0.04	0.32	-0.20	-0.05	0.20	0.53	0.49	-0.30	-0.22	-0.06
**67**	-0.14	0.02	-0.20	0.43	-0.22	-0.33	-0.23	0.31	0.09	-0.21	0.31	-0.45	-0.24	0.37	-0.29	-0.27	0.37	-0.18	-0.06	-0.17	0.66*	0.50	0.08	0.42	-0.23	-0.36	-0.09	0.25	-0.03
**68**	0.38	0.23	0.16	0.39	0.06	-0.06	0.20	0.22	0.36	0.07	-0.08	0.28	0.11	-0.06	0.15	0.13	0.13	-0.08	0.25	0.15	0.11	-0.08	-0.17	0.08	-0.13	-0.10	-0.41	-0.36	0.13
**69**	0.12	-0.17	0.20	0.01	-0.05	0.17	0.36	0.31	0.27	0.22	0.08	0.27	0.15	0.20	0.20	0.30	0.22	0.18	0.23	0.38	-0.06	0.41	0.19	-0.20	-0.76**	-0.46	-0.10	0.06	0.38
**70**	0.08	0.24	-0.01	0.57	0.24	-0.17	0.16	-0.04	0.31	0.07	0.15	-0.13	-0.01	-0.10	-0.03	-0.10	0.01	-0.06	-0.04	-0.03	0.36	-0.17	-0.48	0.19	-0.11	-0.39	-0.28	-0.01	-0.09
**71**	0.16	0.03	0.39	-0.40	0.26	0.50	0.31	-0.22	-0.04	0.38	-0.06	0.55	0.33	-0.27	0.42	0.46	-0.11	0.29	0.03	0.37	-0.52	-0.23	0.14	-0.27	0.34	0.55	0.21	0.01	0.25
**72**	0.38	0.36	0.17	0.16	0.01	0.10	0.27	0.39	0.38	0.29	0.20	0.57	0.20	.629*	0.22	0.37	0.71**	0.24	0.41	0.41	0.27	0.50	0.52	-0.17	-0.42	-0.04	-0.15	-0.08	0.33
**73**	-0.02	0.29	-0.14	0.15	-0.16	-0.22	-0.29	-0.48	-0.01	-0.08	0.38	-0.27	-0.20	0.05	-0.15	-0.03	0.10	-0.10	-0.55	0.03	0.42	0.11	-0.04	-0.17	0.10	-0.18	0.46	0.52	0.16
**74**	0.24	0.03	0.16	0.22	-0.05	-0.03	0.18	-0.31	0.29	0.05	-0.01	0.07	0.08	-0.23	0.11	0.09	-0.06	-0.06	-0.15	0.12	-0.09	-0.21	-0.39	-0.08	-0.06	-0.26	0.01	-0.13	0.21
**75**	-0.48	0.14	-0.27	-0.45	-0.01	-0.17	-0.31	-0.31	-0.57	-0.22	-0.48	-0.12	-0.27	-0.51	-0.24	-0.29	-0.40	-0.32	0.03	-0.35	-0.46	-0.42	-0.13	-0.08	.671*	.636*	-0.16	-0.29	-0.45
**76**	0.25	0.12	0.18	0.19	-0.05	-0.01	0.21	-0.36	0.31	0.10	0.05	0.15	0.09	-0.14	0.13	0.15	0.07	-0.02	-0.14	0.17	-0.06	-0.15	-0.31	-0.15	-0.03	-0.20	0.07	-0.09	0.25
**77**	0.55	-0.05	0.66*	0.24	0.43	.657*	0.27	0.48	0.34	.587*	0.34	0.48	.671*	0.18	0.72**	0.55	0.21	0.49	0.05	0.50	0.05	0.27	0.587*	0.56	0.24	.615*	0.17	0.22	0.44
**78**	0.06	0.21	-0.08	-0.14	0.02	-0.06	0.24	0.04	0.08	-0.01	-0.29	0.36	-0.06	0.00	-0.04	0.16	0.00	0.01	0.26	0.18	-0.15	-0.08	-0.01	-0.58*	-0.52	-0.31	-0.29	-0.28	0.08
**79**	-0.12	0.03	-0.03	-0.05	-0.17	-0.06	-0.02	0.48	0.00	0.01	-0.10	0.11	-0.05	0.29	-0.01	0.09	0.31	-0.05	0.24	0.18	0.06	.601*	0.57	0.03	-0.48	-0.03	-0.22	-0.02	0.20
**80**	0.23	0.03	0.24	0.14	-0.08	0.06	0.29	-0.22	0.31	0.11	0.08	0.20	0.08	-0.12	0.14	0.22	0.14	0.01	-0.04	0.24	-0.06	-0.06	-0.28	-0.15	-0.13	-0.23	0.01	-0.06	0.31
**81**	0.31	0.01	0.30	0.18	0.02	0.13	0.34	-0.22	0.35	0.17	0.10	0.23	0.17	-0.16	0.22	0.25	0.06	0.08	-0.06	0.26	-0.09	-0.14	-0.31	-0.10	-0.08	-0.20	0.04	-0.06	0.31
**82**	-0.50	0.13	-0.65*	-0.27	-0.42	-0.60*	-0.67*	-0.39	-0.49	-0.64*	-0.41	-0.55	-0.57	-0.07	-0.63*	-0.55	-0.26	-0.48	-0.38	-0.52	0.01	-0.11	0.04	-0.20	0.19	0.03	0.12	-0.10	-0.37
**83**	0.23	0.03	0.24	0.14	-0.08	0.06	0.29	-0.22	0.31	0.11	0.08	0.20	0.08	-0.12	0.14	0.22	0.14	0.01	-0.04	0.24	-0.06	-0.06	-0.28	-0.15	-0.13	-0.23	0.01	-0.06	0.31
**84**	0.39	-0.25	0.56	0.16	0.28	0.57	0.16	0.31	0.24	0.45	0.28	0.22	0.58*	0.13	0.59*	0.38	0.09	0.41	-0.12	0.34	-0.06	0.25	0.50	0.61*	0.27	0.52	0.32	0.24	0.38
**85**	0.31	0.29	0.15	0.31	0.29	0.15	-0.10	0.48	0.06	0.14	0.33	0.16	0.17	0.23	0.17	0.10	0.27	0.13	0.19	0.04	0.51	0.09	0.29	0.47	0.47	0.54	-0.12	0.11	-0.08
**86**	0.39	0.19	0.28	0.20	0.29	0.32	0.06	0.48	0.14	0.25	0.38	0.33	0.29	0.36	0.29	0.25	0.39	0.29	0.26	0.16	0.40	0.15	0.37	0.38	0.41	0.55	-0.01	0.12	0.04
**87**	0.64*	-0.11	0.64*	0.30	0.36	0.66*	0.25	0.48	0.45	0.54	0.47	0.39	0.67*	0.37	0.70*	0.66*	0.23	0.59*	-0.25	0.66*	0.25	0.49	0.78**	0.42	-0.17	0.27	0.43	0.50	0.71*
**89**	-0.03	-0.20	0.20	0.33	0.20	0.14	0.38	0.39	0.31	0.26	0.12	-0.01	0.17	0.04	0.17	0.15	0.06	0.16	0.11	0.27	0.10	0.34	-0.01	0.22	-0.70*	-0.53	-0.26	0.15	0.23
**90**	0.28	0.39	0.10	0.57	0.48	0.11	0.10	0.48	0.30	0.20	0.27	0.13	0.21	0.40	0.13	0.11	0.31	0.27	0.09	0.09	0.64*	0.11	0.27	0.46	0.09	0.19	-0.21	0.08	-0.06
**91**	0.31	0.05	0.01	0.17	-0.12	-0.01	-0.24	0.48	0.01	-0.09	0.38	0.01	-0.03	0.26	0.02	0.08	0.21	-0.03	0.10	0.08	0.56	0.31	0.27	0.12	-0.06	-0.01	0.02	0.28	0.13
**92**	0.33	-0.23	0.73**	0.29	0.36	0.64*	0.51	0.13	0.50	0.66*	0.26	0.31	0.69*	0.10	0.71*	0.55	0.21	0.52	-0.23	0.57	-0.19	0.35	0.41	0.58*	-0.01	0.28	0.30	0.25	.622*
**94**	0.38	0.25	0.22	0.21	0.14	0.20	-0.05	0.48	0.13	0.20	0.34	0.31	0.23	0.43	0.24	0.19	0.50	0.17	0.30	0.13	0.40	0.27	0.47	0.40	0.41	0.58*	-0.04	0.03	0.05
**95**	-0.30	0.14	-0.27	0.12	-0.02	-0.28	0.09	-0.31	0.09	-0.16	-0.15	-0.24	-0.24	0.10	-0.33	-0.16	0.02	-0.03	-0.27	-0.09	0.11	-0.01	-0.17	-0.24	-0.43	-0.55	-0.01	0.01	-0.05
**96**	-0.42	0.27	-0.39	0.12	0.12	-0.34	-0.05	0.13	-0.13	-0.24	-0.25	-0.23	-0.34	0.02	-0.41	-0.29	-0.03	-0.17	0.09	-0.24	0.24	-0.03	-0.10	-0.05	-0.24	-0.24	-0.44	-0.13	-0.35
**98**	0.11	-0.42	0.18	-0.06	-0.15	0.25	-0.07	-0.44	0.08	0.12	0.54	-0.29	0.18	0.22	0.14	0.12	0.02	0.29	-0.66*	0.11	0.08	0.21	0.11	0.06	0.02	-0.21	0.93**	0.69*	0.34
**99**	0.41	-0.34	0.48	0.30	0.22	0.55	0.26	-0.04	0.42	0.44	0.83**	-0.05	0.48	0.42	0.44	0.43	0.21	0.62*	-0.56	0.43	0.41	0.38	0.25	0.27	-0.22	-0.30	0.83**	0.89**	0.57
**100**	-0.01	-0.09	0.12	0.05	-0.13	0.03	0.24	-0.36	0.26	0.16	0.14	-0.01	0.06	0.06	0.08	0.18	0.12	0.11	-0.31	0.27	-0.09	0.23	-0.06	-0.26	-0.51	-0.54	0.30	0.25	0.40
**102**	0.03	0.41	0.06	-0.45	-0.16	0.06	0.09	-0.04	-0.13	0.10	-0.14	.615*	-0.07	0.06	0.06	0.33	0.38	-0.03	0.35	0.30	-0.24	0.14	0.36	-0.57	0.10	0.44	-0.09	-0.18	0.21
**104**	-0.01	0.12	-0.04	-0.07	-0.20	-0.11	0.11	-0.39	0.07	-0.14	0.07	0.03	-0.20	-0.02	-0.18	0.06	0.15	-0.06	-0.16	0.06	0.06	-0.10	-0.28	-0.41	-0.07	-0.27	0.14	0.06	0.15

M: Maize flour (peaks numbered according to Table 2); **B**: *Broas* (peaks numbered according to Table 2); *p*-Value corresponds to the significance level of Spearman correlation coefficient indicated as *: significant at $p < 0.05$; **: significant at $p < 0.01$.

Table S8: Spearman correlation coefficients between *broas'* volatile compounds and (1) the major phenolic compounds and (2) total carotenoids content of both *broas* and maize flours.

	Maize flours													
V \ Var	pCA	FA	DCS*ct*	DCS*tt*	DCS$_T$	DFP*cc*	DFP*ct*	DFP*tt*	DFP$_T$	CFP*ct*	CFP*tt*	CFP$_T$	bisDFP	Carot
**1**	-0.459	-0.383	0.099	-0.106	-0.106	0.384	-0.077	-0.373	-0.352	0.134	-0.183	-0.141	-0.097	0.739**
**2**	0.158	0.208	0.098	-0.028	-0.028	-0.106	0.140	0.049	0.147	0.067	0.028	0.084	0.004	-0.783**
**3**	-0.140	-0.352	0.039	-0.049	-0.049	-0.116	-0.364	-0.510	-0.441	-0.326	-0.455	-0.434	-0.099	0.154
**4**	-0.523	-0.648*	-0.417	-0.497	-0.497	-0.028	-0.357	-0.350	-0.420	-0.568	-0.399	-0.483	-0.334	-0.147
**5**	0.039	-0.254	-0.067	-0.035	-0.035	-0.279	-0.357	-0.371	-0.322	-0.379	-0.266	-0.294	-0.199	-0.238
**6**	-0.365	-0.085	-0.242	-0.399	-0.399	0.268	0.182	-0.098	0.049	-0.011	-0.140	-0.112	-0.096	-0.224
**7**	0.460	0.662*	0.144	0.252	0.252	0.085	0.315	0.245	0.357	0.596*	0.371	0.497	-0.018	-0.077
**8**	0.414	0.120	-0.340	0.070	0.070	-0.328	0.014	0.364	0.210	-0.018	0.385	0.322	-0.345	-0.308
**9**	0.551	0.486	0.340	0.552	0.552	-0.102	0.350	0.608*	0.476	0.533	0.650*	0.678*	0.099	0.007
**10+11**	0.102	0.004	0.238	0.252	0.252	-0.011	0.371	0.601*	0.455	0.179	0.490	0.420	0.259	-0.112
**12**	0.046	-0.243	0.077	0.000	0.000	-0.384	-0.538	-0.490	-0.476	-0.540	-0.469	-0.510	0.018	-0.308
**13**	-0.295	-0.151	-0.224	-0.308	-0.308	0.504	0.252	-0.014	0.196	0.196	0.203	0.196	-0.068	0.350
**14**	-0.098	-0.229	-0.179	-0.196	-0.196	-0.183	-0.392	-0.406	-0.343	-0.516	-0.406	-0.455	-0.092	-0.245
**15**	-0.418	-0.602*	-0.301	-0.336	-0.336	-0.046	-0.252	-0.140	-0.252	-0.572	-0.273	-0.406	-0.057	0.014
**16**	0.432	0.687*	0.140	0.231	0.231	0.095	0.301	0.189	0.322	0.579*	0.294	0.427	0.018	-0.049
**17**	0.446	0.673*	0.130	0.238	0.238	0.113	0.336	0.238	0.364	0.604*	0.357	0.483	0.011	-0.028
**18**	0.042	-0.095	0.095	0.196	0.196	0.120	0.322	0.615*	0.448	0.207	0.622*	0.531	0.075	0.161
**19**	-0.119	-0.363	-0.305	-0.259	-0.259	-0.067	-0.441	-0.573	-0.580*	-0.186	-0.301	-0.245	-0.700*	-0.056
**20**	0.537	0.585*	0.126	0.287	0.287	-0.092	0.196	0.273	0.280	0.502	0.406	0.517	-0.195	-0.336
**21**	0.425	0.176	0.259	0.476	0.476	-0.190	0.294	0.643*	0.455	0.291	0.608*	0.587*	0.021	-0.105
**22**	0.018	-0.176	-0.060	-0.021	-0.021	-0.430	-0.713**	-0.517	-0.545	-0.765**	-0.601*	-0.692*	0.068	-0.133
**23**	-0.232	-0.504	-0.284	-0.217	-0.217	-0.384	-0.811**	-0.671*	-0.797**	-0.793**	-0.706*	-0.755**	-0.441	-0.098
**25**	-0.154	0.007	-0.333	-0.399	-0.399	0.413	0.259	-0.161	0.112	0.316	0.077	0.168	-0.291	0.049
**26**	-0.056	0.042	-0.347	-0.266	-0.266	0.194	-0.098	-0.252	-0.070	0.014	0.000	0.028	-0.384	0.021
**27**	0.098	0.176	-0.123	-0.266	-0.266	0.134	0.217	-0.175	-0.021	0.368	-0.035	0.126	-0.288	-0.531
**28**	0.158	0.261	-0.039	-0.014	-0.014	-0.113	-0.035	0.021	-0.112	0.077	-0.077	-0.049	0.025	-0.231
**29**	-0.049	-0.218	-0.077	-0.133	-0.133	-0.152	-0.573	-0.615*	-0.608*	-0.389	-0.469	-0.455	-0.266	-0.105
**30**	0.344	0.373	0.028	0.049	0.049	-0.152	-0.112	-0.252	-0.175	0.214	-0.147	0.007	-0.266	-0.371
**32**	-0.084	-0.285	0.021	-0.098	-0.098	-0.307	-0.608*	-0.657*	-0.664*	-0.551	-0.629*	-0.643*	-0.082	-0.238
**33+34**	0.011	-0.120	-0.448	-0.252	-0.252	-0.286	-0.385	-0.105	-0.098	-0.646*	-0.196	-0.322	-0.160	-0.392
**35**	0.267	0.282	-0.109	0.028	0.028	-0.018	0.126	-0.070	0.056	0.330	0.070	0.196	-0.320	-0.077
**36**	-0.284	-0.222	-0.371	-0.538	-0.538	0.173	0.021	-0.245	-0.182	-0.028	-0.245	-0.168	-0.359	-0.413
**37**	0.228	0.201	0.035	0.056	0.056	-0.004	0.147	-0.168	-0.042	0.382	0.007	0.140	-0.195	-0.035
**38+39**	-0.021	0.063	0.147	-0.189	-0.189	-0.025	0.063	-0.392	-0.175	0.140	-0.357	-0.196	-0.043	-0.650*
**41**	0.302	0.204	0.095	0.070	0.070	-0.413	-0.217	-0.056	-0.168	-0.175	-0.175	-0.161	0.028	-0.755**
**42**	0.028	0.092	-0.536	-0.371	-0.371	0.028	-0.224	-0.322	-0.287	-0.004	-0.175	-0.084	-0.679*	-0.217
**43**	-0.375	-0.215	0.158	-0.126	-0.126	0.384	0.434	0.091	0.140	0.456	0.175	0.259	-0.078	0.063
**44**	-0.165	-0.380	-0.413	-0.301	-0.301	-0.335	-0.657*	-0.420	-0.615*	-0.653*	-0.434	-0.524	-0.433	-0.280
**45**	-0.105	-0.363	-0.574	-0.287	-0.287	-0.250	-0.629*	-0.399	-0.601*	-0.533	-0.294	-0.364	-0.718**	-0.049
**46**	0.166	-0.057	0.056	0.310	0.310	-0.050	0.148	0.507	0.275	0.173	0.556	0.493	-0.182	0.197
**47**	-0.214	0.243	0.151	-0.070	-0.070	0.526	0.490	0.021	0.273	0.519	0.077	0.196	0.195	0.308
**51**	-0.470	-0.299	0.238	-0.196	-0.196	0.402	0.140	-0.231	-0.168	0.249	-0.091	-0.014	-0.011	0.035
**52**	0.165	-0.081	0.025	0.287	0.287	-0.236	-0.098	0.392	0.119	-0.137	0.336	0.238	-0.153	0.007
**53**	0.530	0.676*	0.245	0.406	0.406	-0.138	0.252	0.273	0.315	0.505	0.336	0.469	-0.131	-0.350
**54**	-0.154	-0.144	0.504	0.231	0.231	0.102	0.273	0.273	0.154	0.133	0.056	0.042	0.558	0.196
**55+56**	-0.214	-0.500	0.161	-0.133	-0.133	-0.198	-0.238	-0.259	-0.245	-0.439	-0.329	-0.385	0.185	-0.392
**57**	0.172	0.000	-0.007	0.231	0.231	-0.208	-0.049	0.420	0.147	-0.105	0.336	0.231	-0.060	-0.049
**58**	-0.274	-0.563	-0.025	-0.112	-0.112	0.032	-0.168	-0.210	-0.287	-0.074	-0.077	-0.091	-0.234	0.315
**59**	-0.312	-0.496	0.284	0.147	0.147	0.233	0.098	0.063	-0.028	0.168	0.203	0.189	-0.043	0.573
**60+61**	-0.333	-0.408	0.546	0.133	0.133	0.071	0.168	-0.021	-0.084	0.039	-0.210	-0.196	0.515	0.224
**62**	0.074	0.246	-0.385	-0.280	-0.280	-0.240	-0.483	-0.448	-0.413	-0.477	-0.573	-0.594*	-0.018	-0.252
**63**	0.170	-0.110	-0.159	0.169	0.169	-0.188	-0.092	0.345	0.077	-0.081	0.373	0.289	-0.358	0.014
**64+65**	0.375	0.373	0.179	0.280	0.280	-0.081	0.224	0.070	0.133	0.516	0.238	0.399	-0.334	-0.238
**66**	-0.021	-0.046	-0.172	-0.126	-0.126	-0.148	-0.070	-0.014	-0.161	-0.091	-0.140	-0.133	-0.167	-0.245
**67**	-0.684*	-0.722**	-0.357	-0.573	-0.573	0.162	-0.161	-0.371	-0.301	-0.396	-0.378	-0.420	-0.277	-0.021
**68**	0.154	-0.201	-0.238	0.021	0.021	-0.282	-0.168	0.182	-0.035	-0.186	0.231	0.168	-0.483	-0.343
**69**	0.140	-0.190	-0.193	0.007	0.007	-0.303	-0.524	-0.238	-0.322	-0.372	-0.063	-0.112	-0.465	-0.168
**70**	-0.235	-0.644*	0.235	0.112	0.112	-0.035	0.091	0.168	0.007	-0.084	0.154	0.077	0.064	0.168
**71**	0.526	0.673*	0.280	0.427	0.427	-0.099	0.112	0.140	0.119	0.453	0.182	0.287	0.103	0.063
**72**	0.235	-0.162	-0.046	0.189	0.189	-0.705*	-0.853**	-0.434	-0.643*	-0.768**	-0.490	-0.559	-0.316	-0.350
**73**	-0.670*	-0.620*	0.028	-0.231	-0.231	0.413	-0.056	-0.280	-0.336	-0.007	-0.182	-0.189	-0.107	0.657*

**74**	0.088	-0.162	-0.049	0.203	0.203	-0.018	0.098	0.420	0.196	0.126	0.510	0.448	-0.309	0.196
**75**	0.168	0.556	0.462	0.175	0.175	0.215	0.531	0.182	0.385	0.593*	0.119	0.259	0.508	-0.217
**76**	0.118	-0.149	0.046	0.303	0.303	-0.111	-0.004	0.356	0.100	0.055	0.413	0.349	-0.257	0.238
**77**	0.042	0.176	-0.231	-0.252	-0.252	0.088	0.070	-0.287	-0.154	0.249	-0.203	-0.042	-0.380	-0.238
**78**	0.474	0.113	0.056	0.371	0.371	-0.511	-0.469	0.056	-0.147	-0.368	0.112	0.000	-0.089	-0.210
**79**	-0.049	-0.088	-0.280	-0.315	-0.315	-0.152	-0.413	-0.510	-0.371	-0.309	-0.350	-0.301	-0.497	-0.580*
**80**	0.158	-0.088	-0.102	0.231	0.231	-0.169	-0.091	0.315	0.063	0.000	0.385	0.315	-0.366	0.175
**81**	0.189	-0.067	-0.070	0.259	0.259	-0.138	-0.014	0.371	0.119	0.074	0.441	0.378	-0.323	0.210
**82**	-0.225	0.120	-0.025	-0.168	-0.168	0.325	0.266	0.063	0.224	0.105	0.042	0.035	0.227	0.021
**83**	0.158	-0.088	-0.102	0.231	0.231	-0.169	-0.091	0.315	0.063	0.000	0.385	0.315	-0.366	0.175
**84**	-0.018	0.229	-0.235	-0.266	-0.266	0.258	0.280	-0.133	0.063	0.393	-0.049	0.112	-0.302	-0.112
**85**	-0.274	-0.123	-0.210	-0.406	-0.406	0.032	-0.063	-0.378	-0.315	-0.133	-0.517	-0.455	-0.018	-0.203
**86**	-0.046	0.081	-0.196	-0.217	-0.217	-0.162	-0.189	-0.322	-0.315	-0.214	-0.510	-0.469	0.014	-0.161
**87**	-0.130	-0.021	-0.567	-0.469	-0.469	0.201	-0.112	-0.434	-0.308	0.084	-0.210	-0.105	-0.675*	-0.014
**89**	-0.123	-0.475	-0.049	-0.126	-0.126	-0.042	-0.126	-0.175	-0.133	-0.112	0.028	0.021	-0.323	-0.147
**90**	-0.109	-0.254	0.007	-0.091	-0.091	-0.212	-0.077	-0.217	-0.217	-0.291	-0.378	-0.399	0.160	-0.168
**91**	-0.547	-0.426	-0.760**	-0.818**	-0.818**	0.194	-0.448	-0.587*	-0.580*	-0.425	-0.524	-0.538	-0.561	-0.049
**92**	0.179	0.165	0.046	0.126	0.126	0.085	0.343	0.084	0.203	0.505	0.252	0.399	-0.362	-0.042
**94**	-0.102	0.014	-0.214	-0.266	-0.266	-0.180	-0.266	-0.406	-0.392	-0.288	-0.580*	-0.524	-0.110	-0.301
**95**	0.095	-0.176	0.315	0.413	0.413	-0.201	0.007	0.252	0.161	-0.161	0.231	0.105	0.288	0.238
**96**	-0.011	-0.165	0.256	0.105	0.105	-0.183	0.000	-0.021	0.028	-0.204	-0.091	-0.154	0.366	-0.203
**98**	-0.411	-0.160	-0.118	-0.154	-0.154	0.489	0.147	-0.060	-0.007	0.185	-0.007	0.004	0.007	0.876**
**99**	-0.442	-0.405	-0.231	-0.238	-0.238	0.342	-0.042	-0.252	-0.245	0.000	-0.168	-0.175	-0.149	0.853**
**100**	-0.032	-0.303	0.135	0.253	0.253	-0.022	-0.146	0.057	-0.064	-0.030	0.235	0.185	-0.235	0.388
**102**	0.432	0.440	0.084	0.301	0.301	-0.487	-0.545	-0.238	-0.378	-0.214	-0.245	-0.217	-0.188	-0.308
**104**	0.116	0.018	-0.067	0.273	0.273	-0.190	-0.161	0.287	0.035	-0.140	0.245	0.119	-0.053	0.399

	Broas												
**V \ Var**	**pCA**	**FA**	**DCS*ct***	**DCS*tt***	**DCST**	**DFP*cc***	**DFP*ct***	**DFP*tt***	**DFPT**	**CFP*ct***	**CFP*tt***	**CFPT**	**Carot**
**1**	-0.042	0.077	-0.060	0.099	0.014	-0.068	-0.081	-0.254	-0.232	0.174	0.149	0.166	0.754**
**2**	-0.077	0.035	-0.204	-0.322	-0.294	-0.029	0.004	0.224	0.147	-0.461	-0.046	-0.140	-0.776**
**3**	-0.147	-0.119	-0.277	-0.098	-0.168	0.064	-0.519	-0.524	-0.517	-0.320	-0.313	-0.340	0.161
**4**	-0.573	-0.559	-0.172	-0.524	-0.497	0.136	-0.053	-0.168	-0.154	-0.345	-0.148	-0.095	-0.119
**5**	-0.182	0.007	-0.540	-0.168	-0.294	-0.476	-0.681*	-0.133	-0.175	-0.563	-0.004	-0.063	-0.217
**6**	-0.497	-0.287	-0.193	-0.503	-0.420	0.236	0.386	0.231	0.196	-0.106	0.148	0.098	-0.217
**7**	0.371	0.322	0.042	0.224	0.238	0.061	0.067	0.091	0.084	0.405	0.084	0.046	-0.133
**8**	0.014	-0.175	-0.084	-0.035	-0.049	-0.233	-0.302	0.056	0.098	-0.070	-0.067	-0.018	-0.343
**9**	0.671*	0.497	0.411	0.524	0.510	-0.204	0.021	0.175	0.189	0.299	0.056	0.081	-0.014
**10+11**	0.301	0.140	0.344	0.224	0.238	-0.140	0.274	0.413	0.420	-0.021	0.123	0.161	-0.098
**12**	-0.091	0.063	-0.393	-0.077	-0.189	-0.408	-0.621*	-0.210	-0.266	-0.609*	-0.134	-0.186	-0.266
**13**	-0.210	-0.154	-0.642*	-0.126	-0.238	-0.265	-0.077	0.427	0.441	0.123	0.650*	0.599*	0.294
**14**	-0.329	-0.126	-0.481	-0.308	-0.371	-0.240	-0.460	-0.077	-0.119	-0.567	0.018	-0.067	-0.224
**15**	-0.434	-0.476	-0.102	-0.329	-0.322	0.050	-0.060	-0.049	-0.021	-0.398	-0.077	-0.046	0.042
**16**	0.322	0.329	0.056	0.210	0.238	0.097	0.130	0.077	0.063	0.430	0.081	0.035	-0.098
**17**	0.336	0.315	0.018	0.217	0.231	0.061	0.084	0.105	0.098	0.405	0.112	0.063	-0.084
**18**	0.364	0.084	0.239	0.287	0.252	-0.200	0.098	0.392	0.441	0.155	0.278	0.343	0.140
**19**	-0.301	-0.252	-0.540	-0.413	-0.517	-0.111	-0.828**	-0.594*	-0.580*	-0.475	-0.243	-0.280	-0.077
**20**	0.420	0.294	0.105	0.154	0.168	0.021	-0.102	-0.014	-0.021	0.211	-0.084	-0.102	-0.385
**21**	0.552	0.224	0.435	0.364	0.371	-0.025	-0.060	0.056	0.098	0.085	-0.162	-0.105	-0.126
**22**	-0.112	0.063	-0.193	-0.007	-0.063	-0.276	-0.477	-0.245	-0.287	-0.394	-0.116	-0.147	-0.084
**23**	-0.343	-0.280	-0.049	-0.336	-0.329	0.147	-0.547	-0.734**	-0.727**	-0.451	-0.524	-0.497	-0.056
**25**	-0.385	-0.350	-0.723**	-0.392	-0.448	0.054	-0.137	0.112	0.126	0.032	0.278	0.207	-0.028
**26**	-0.217	-0.056	-0.660*	-0.238	-0.329	-0.283	-0.379	0.133	0.119	0.004	0.422	0.350	-0.028
**27**	-0.329	-0.315	-0.495	-0.573	-0.566	0.265	-0.214	-0.196	-0.217	-0.493	-0.264	-0.389	-0.580*
**28**	-0.056	-0.007	0.249	-0.105	-0.028	0.132	0.179	-0.112	-0.126	-0.141	-0.225	-0.249	-0.210
**29**	-0.140	-0.007	-0.467	-0.231	-0.343	-0.218	-0.768**	-0.434	-0.462	-0.595*	-0.137	-0.231	-0.098
**30**	0.056	0.021	-0.095	-0.161	-0.133	0.297	-0.291	-0.476	-0.490	-0.109	-0.446	-0.504	-0.406
**32**	-0.238	0.035	-0.375	-0.231	-0.322	-0.311	-0.621*	-0.371	-0.434	-0.732**	-0.200	-0.287	-0.182
**33+34**	-0.301	-0.301	-0.319	-0.280	-0.273	-0.122	-0.232	0.147	0.140	-0.229	0.088	0.088	-0.399
**35**	-0.049	-0.056	-0.204	-0.112	-0.105	0.175	-0.211	-0.287	-0.273	0.116	-0.211	-0.238	-0.126
**36**	-0.566	-0.720**	-0.270	-0.755**	-0.664*	0.666*	0.021	-0.329	-0.301	-0.401	-0.429	-0.466	-0.455
**37**	-0.084	0.049	-0.337	-0.084	-0.140	-0.097	-0.302	-0.245	-0.259	-0.113	-0.137	-0.200	-0.056
**38+39**	-0.301	-0.168	-0.316	-0.469	-0.434	0.297	-0.011	-0.231	-0.301	-0.447	-0.397	-0.483	-0.643*
**41**	0.021	0.042	0.116	-0.175	-0.126	0.004	-0.098	-0.119	-0.175	-0.507	-0.383	-0.424	-0.720**
**42**	-0.371	-0.427	-0.309	-0.538	-0.490	0.401	-0.330	-0.476	-0.441	-0.102	-0.295	-0.333	-0.280
**43**	-0.098	-0.063	0.133	-0.140	-0.105	0.168	0.463	0.126	0.119	0.158	0.053	0.098	0.077
**44**	-0.371	-0.280	-0.116	-0.364	-0.371	-0.168	-0.379	-0.308	-0.315	-0.447	-0.204	-0.172	-0.238
**45**	-0.378	-0.301	-0.260	-0.399	-0.441	-0.190	-0.674*	-0.462	-0.434	-0.437	-0.165	-0.161	-0.042
**46**	0.416	0.176	0.350	0.317	0.289	-0.187	-0.120	0.070	0.127	0.174	0.067	0.148	0.183
**47**	-0.077	0.147	-0.049	-0.028	0.014	0.222	0.502	0.252	0.224	0.349	0.320	0.245	0.294
**51**	-0.091	0.049	-0.088	-0.189	-0.224	-0.014	0.175	0.070	0.028	-0.165	0.144	0.116	0.063
**52**	0.378	0.147	0.463	0.259	0.273	-0.089	-0.116	-0.021	0.021	0.070	-0.084	0.004	0.014
**53**	0.420	0.490	0.288	0.189	0.252	0.050	0.084	0.007	-0.035	0.268	-0.098	-0.130	-0.364
**54**	0.266	0.098	0.607*	0.266	0.329	0.322	0.481	0.000	0.014	-0.011	-0.239	-0.203	0.238
**55+56**	-0.147	-0.238	-0.228	-0.175	-0.231	-0.075	-0.204	-0.049	-0.077	-0.553	-0.225	-0.210	-0.357

**57**	0.308	0.140	0.432	0.224	0.245	-0.157	0.004	0.119	0.147	0.039	0.011	0.081	-0.035
**58**	-0.035	-0.273	-0.056	0.035	-0.042	0.014	-0.256	-0.364	-0.301	0.056	-0.214	-0.095	0.308
**59**	0.280	0.112	0.179	0.301	0.210	-0.107	-0.098	-0.119	-0.070	0.173	0.056	0.147	0.580*
**60+61**	0.084	-0.028	0.491	0.168	0.210	0.358	0.411	-0.224	-0.217	-0.106	-0.422	-0.361	0.287
**62**	-0.420	-0.147	-0.189	-0.301	-0.238	0.054	-0.039	-0.140	-0.182	-0.187	-0.077	-0.151	-0.231
**63**	0.232	0.007	0.240	0.106	0.092	-0.144	-0.297	-0.077	-0.021	-0.032	-0.060	0.007	0.000
**64+65**	0.224	0.280	0.035	0.021	0.028	0.043	-0.189	-0.245	-0.266	0.060	-0.236	-0.266	-0.259
**66**	-0.238	-0.273	0.260	-0.280	-0.175	0.326	0.151	-0.273	-0.259	-0.194	-0.415	-0.389	-0.224
**67**	-0.622*	-0.643*	-0.228	-0.510	-0.476	0.283	0.175	-0.098	-0.077	-0.063	-0.091	0.000	-0.007
**68**	0.028	-0.175	-0.007	-0.168	-0.189	-0.132	-0.449	-0.147	-0.112	-0.338	-0.200	-0.158	-0.357
**69**	0.119	0.000	-0.277	-0.007	-0.112	-0.319	-0.740**	-0.259	-0.245	-0.180	-0.056	-0.025	-0.189
**70**	0.091	-0.133	0.112	0.112	0.035	-0.143	-0.165	-0.077	-0.035	-0.296	-0.165	-0.081	0.196
**71**	0.448	0.531	0.288	0.378	0.392	-0.050	-0.011	-0.119	-0.140	0.254	-0.067	-0.119	0.056
**72**	0.077	0.161	0.095	-0.014	-0.042	-0.215	-0.754**	-0.601*	-0.629*	-0.532	-0.485	-0.483	-0.294
**73**	-0.161	-0.126	0.095	-0.042	-0.077	0.089	0.119	-0.196	-0.161	0.102	0.084	0.144	0.685*
**74**	0.301	0.063	0.221	0.196	0.154	-0.186	-0.221	0.014	0.077	0.095	0.063	0.140	0.175
**75**	0.182	0.329	0.158	0.119	0.189	0.190	0.600*	0.322	0.252	0.180	0.074	-0.007	-0.210
**76**	0.374	0.185	0.355	0.292	0.256	-0.199	-0.234	-0.089	-0.036	0.099	-0.020	0.062	0.238
**77**	-0.371	-0.336	-0.242	-0.497	-0.427	0.512	-0.098	-0.462	-0.455	-0.165	-0.418	-0.490	-0.280
**78**	0.413	0.448	-0.081	0.329	0.196	-0.762**	-0.737**	0.042	0.014	-0.345	0.141	0.116	-0.189
**79**	-0.301	-0.182	-0.530	-0.476	-0.503	-0.047	-0.442	-0.182	-0.231	-0.320	-0.084	-0.126	-0.594*
**80**	0.315	0.126	0.326	0.259	0.238	-0.204	-0.214	-0.084	-0.028	0.229	-0.004	0.105	0.168
**81**	0.336	0.119	0.312	0.273	0.245	-0.190	-0.232	-0.084	-0.021	0.190	-0.014	0.081	0.196
**82**	-0.189	0.105	-0.182	-0.112	-0.098	-0.157	0.411	0.566	0.510	0.070	0.545	0.469	0.035
**83**	0.315	0.126	0.326	0.259	0.238	-0.204	-0.214	-0.084	-0.028	0.229	-0.004	0.105	0.168
**84**	-0.357	-0.329	-0.232	-0.448	-0.364	0.551	0.116	-0.252	-0.238	0.053	-0.228	-0.294	-0.168
**85**	-0.594*	-0.497	-0.032	-0.517	-0.399	0.551	0.302	-0.336	-0.343	-0.236	-0.443	-0.462	-0.182
**86**	-0.427	-0.308	0.144	-0.315	-0.182	0.483	0.249	-0.392	-0.399	-0.099	-0.478	-0.487	-0.133
**87**	-0.566	-0.434	-0.593*	-0.615*	-0.622*	0.197	-0.379	-0.378	-0.357	-0.187	-0.077	-0.158	-0.070
**89**	-0.056	-0.245	-0.463	-0.147	-0.273	-0.204	-0.540	-0.133	-0.112	-0.268	-0.063	-0.021	-0.175
**90**	-0.399	-0.217	-0.109	-0.245	-0.231	0.000	-0.032	-0.168	-0.196	-0.518	-0.299	-0.333	-0.112
**91**	-0.776**	-0.804**	-0.382	-0.741**	-0.685*	0.415	-0.032	-0.329	-0.287	-0.113	-0.127	-0.098	-0.077
**92**	0.035	0.021	-0.098	-0.098	-0.091	0.193	-0.151	-0.245	-0.231	0.092	-0.207	-0.228	-0.084
**94**	-0.462	-0.350	0.130	-0.441	-0.294	0.580*	0.182	-0.483	-0.497	-0.201	-0.569	-0.585*	-0.273
**95**	0.336	0.476	0.056	0.497	0.371	-0.759**	-0.200	0.364	0.336	-0.070	0.376	0.399	0.294
**96**	-0.014	0.161	-0.182	0.112	0.035	-0.483	-0.032	0.322	0.259	-0.310	0.169	0.154	-0.147
**98**	-0.098	-0.091	0.121	0.116	0.133	0.269	0.315	-0.067	-0.004	0.564	0.231	0.270	0.855**
**99**	-0.273	-0.280	-0.063	-0.007	-0.035	0.175	0.004	-0.308	-0.231	0.303	0.046	0.098	0.839**
**100**	0.374	0.278	0.055	0.342	0.221	-0.384	-0.445	-0.096	-0.064	0.106	0.152	0.216	0.388
**102**	0.322	0.503	0.347	0.203	0.238	-0.157	-0.246	-0.329	-0.385	0.035	-0.236	-0.242	-0.266
**104**	0.301	0.322	0.467	0.469	0.462	-0.397	0.074	0.140	0.168	0.440	0.243	0.357	0.434

Var: Variables; **V**: *Broas'* volatiles (peaks numbered according to Table 2); **TPC**: Total phenolic content; AA: Antioxidant activity: **pCA**: *p*-Coumaric acid; **FA**: Ferulic acid; **DCS**: Dicoumaroyl spermidine; **DFP**: Diferuloyl putrescine; **CFP**: Coumaroyl feruloyl putrescine; ***cc***: *cis,cis* isomer; ***ct***: *cis.trans* isomer; ***tt***: *trans,trans* isomer; **т:** total; **bisDFP**: Bis-diferuloyl putrescine. *p*-Value corresponds to the significance level of Spearman correlation coefficient indicated as *: significant at $p < 0.05$; **: significant at $p < 0.01$.

3.2. Broas' sensory analysis and volatile composition

Table S9: Average *broas'* sensorial analysis scores (SA).

Broas	Appearance	Color	Smell and odor	Taste and aroma	Texture	Global appreciation
**1**	5.90 ± 0.90	6.00 ± 0.83	5.89 ± 1.01	5.73 ± 1.20	5.90 ± 1.10	5.67 ± 1.14
**2**	6.48 ± 0.95	6.71 ± 0.87	5.87 ± 0.82	6.04 ± 0.87	6.17 ± 0.91	6.06 ± 0.73
**3**	6.13 ± 0.70	5.88 ± 0.87	5.91 ± 0.93	6.25 ± 0.93	6.08 ± 0.82	6.15 ± 0.85
**4**	6.42 ± 0.79	6.29 ± 0.90	5.98 ± 0.77	6.17 ± 1.00	6.31 ± 0.80	6.13 ± 0.87
**5**	6.40 ± 0.99	6.36 ± 0.87	5.85 ± 0.87	5.98 ± 1.09	6.15 ± 1.00	6.02 ± 0.94
**6**	6.15 ± 0.93	5.89 ± 0.84	5.96 ± 0.76	6.13 ± 0.95	6.13 ± 0.88	6.00 ± 0.93
**7**	6.19 ± 0.68	5.98 ± 0.77	5.93 ± 0.83	5.85 ± 1.04	6.04 ± 0.93	5.87 ± 0.97
**8**	6.72 ± 1.08	6.89 ± 1.11	6.20 ± 0.88	6.17 ± 1.09	6.36 ± 0.87	6.28 ± 0.99
**9**	5.98 ± 0.69	5.76 ± 0.71	5.93 ± 0.70	5.84 ± 0.90	5.90 ± 1.10	5.89 ± 0.96
**10**	6.76 ± 1.05	6.84 ± 1.09	6.16 ± 0.91	6.20 ± 0.97	6.17 ± 0.91	6.27 ± 1.12
**11**	6.00 ± 0.85	5.73 ± 0.78	5.86 ± 0.93	6.36 ± 0.88	6.08 ± 0.82	6.20 ± 0.89
**12**	5.36 ± 1.05	5.00 ± 0.93	5.39 ± 0.95	4.67 ± 0.88	6.31 ± 0.80	4.58 ± 0.97

Table S10: Spearman correlation coefficients between *broas'* volatile compounds (V) and sensorial analysis scores (SA).

V \ SA	Appearance	Color	Smell and odor	Taste and aroma	Texture	Global appreciation
**1**	0.648*	0.746**	0.370	0.141	0.585*	0.254
**2**	-0.531	-0.832**	-0.196	0.158	-0.406	-0.154
**3**	-0.126	0.084	-0.137	-0.133	-0.042	-0.056
**4**	0.049	-0.210	0.305	0.270	0.301	0.231
**5**	-0.322	-0.350	-0.410	-0.056	-0.224	-0.217
**6**	-0.007	-0.294	0.277	0.287	0.140	0.245
**7**	-0.266	0.014	-0.298	-0.599*	-0.545	-0.545
**8**	-0.315	-0.266	-0.291	-0.539	-0.580*	-0.552
**9**	-0.035	0.105	-0.077	-0.231	-0.301	-0.308
**10+11**	0.140	-0.112	0.242	0.455	0.140	0.301
**12**	-0.322	-0.392	-0.487	0.119	-0.091	-0.077
**13**	0.196	0.252	0.081	-0.028	0.070	0.084
**14**	-0.287	-0.378	-0.154	0.172	-0.056	0.042
**15**	0.168	-0.077	0.368	0.473	0.385	0.462
**16**	-0.231	0.042	-0.305	-0.571	-0.490	-0.497
**17**	-0.224	0.056	-0.284	-0.578*	-0.517	-0.503
**18**	0.224	0.161	0.382	0.249	0.140	0.189
**19**	-0.231	-0.084	-0.014	-0.480	-0.336	-0.545
**20**	-0.441	-0.231	-0.280	-0.627*	-0.685*	-0.706*
**21**	-0.154	-0.056	0.098	-0.126	-0.315	-0.203
**22**	-0.252	-0.217	-0.336	0.161	0.105	0.077
**23**	-0.224	-0.147	0.116	-0.049	0.091	-0.084
**25**	-0.119	0.007	-0.147	-0.497	-0.392	-0.343
**26**	-0.224	-0.056	-0.147	-0.389	-0.280	-0.371
**27**	-0.406	-0.510	-0.144	-0.389	-0.685*	-0.497
**28**	0.077	-0.126	0.077	0.067	-0.021	-0.035
**29**	-0.252	-0.175	-0.070	-0.046	-0.126	-0.203
**30**	-0.517	-0.287	-0.343	-0.676*	-0.685*	-0.692*
**32**	-0.189	-0.322	-0.224	0.242	0.056	0.014
**33+34**	-0.476	-0.497	-0.207	-0.049	-0.203	-0.077
**35**	-0.322	-0.028	-0.343	-0.788**	-0.629*	-0.650*
**36**	-0.266	-0.406	0.294	-0.172	-0.322	-0.154
**37**	-0.119	0.007	-0.448	-0.564	-0.483	-0.524
**38+39**	-0.448	-0.643*	-0.413	-0.133	-0.434	-0.280
**41**	-0.427	-0.706*	-0.256	0.109	-0.315	-0.196
**42**	-0.399	-0.175	0.095	-0.746**	-0.573	-0.657*
**43**	0.371	0.105	0.319	0.231	0.294	0.182
**44**	-0.091	-0.287	0.067	0.074	0.112	-0.091

**45**	-0.098	-0.077	0.252	-0.228	-0.070	-0.315
**46**	0.134	0.225	0.363	-0.035	0.007	-0.085
**47**	0.280	0.287	0.224	0.137	0.210	0.252
**51**	0.350	0.028	0.378	0.515	0.441	0.315
**52**	-0.035	0.021	0.357	0.084	0.049	-0.014
**53**	-0.434	-0.273	-0.235	-0.459	-0.552	-0.573
**54**	0.441	0.231	0.445	0.711**	0.531	0.685*
**55+56**	-0.196	-0.462	-0.270	0.389	0.070	0.224
**57**	0.049	-0.021	0.319	0.193	0.105	0.063
**58**	0.252	0.364	-0.028	-0.235	0.133	-0.105
**59**	0.490	0.573	0.392	0.200	0.448	0.238
**60+61**	0.497	0.259	0.245	0.662*	0.601*	0.678*
**62**	-0.210	-0.252	-0.266	-0.084	-0.063	-0.063
**63**	-0.049	0.028	0.356	-0.116	-0.113	-0.197
**64+65**	-0.357	-0.175	-0.249	-0.606*	-0.608*	-0.678*
**66**	0.014	-0.175	0.207	0.018	-0.021	-0.021
**67**	0.119	-0.077	0.172	0.144	0.336	0.245
**68**	-0.315	-0.350	0.154	-0.193	-0.350	-0.364
**69**	-0.434	-0.196	-0.231	-0.431	-0.350	-0.524
**70**	0.308	0.133	0.228	0.399	0.301	0.329
**71**	-0.049	0.175	-0.210	-0.354	-0.287	-0.364
**72**	-0.497	-0.392	-0.172	-0.067	-0.161	-0.287
**73**	0.734**	0.650*	0.676*	0.438	0.797**	0.510
**74**	0.119	0.210	0.403	-0.088	-0.021	-0.133
**75**	0.000	-0.161	-0.231	0.179	-0.070	0.119
**76**	0.149	0.260	0.390	-0.025	0.071	-0.085
**77**	-0.301	-0.189	-0.049	-0.588*	-0.545	-0.497
**78**	-0.322	-0.252	-0.294	-0.007	-0.217	-0.287
**79**	-0.657*	-0.636*	-0.350	-0.378	-0.462	-0.531
**80**	0.070	0.224	0.238	-0.235	-0.021	-0.252
**81**	0.098	0.259	0.266	-0.231	-0.056	-0.238
**82**	0.189	-0.063	0.161	0.487	0.329	0.420
**83**	0.070	0.224	0.238	-0.235	-0.021	-0.252
**84**	-0.210	-0.077	0.028	-0.557	-0.476	-0.392
**85**	0.056	-0.161	0.025	0.035	0.056	0.126
**86**	-0.021	-0.098	-0.098	-0.102	-0.007	0.021
**87**	-0.175	-0.021	0.081	-0.648*	-0.434	-0.538
**89**	-0.259	-0.196	-0.270	-0.287	-0.294	-0.336
**90**	0.063	-0.217	-0.175	0.308	0.112	0.266
**91**	0.042	-0.028	0.305	-0.228	0.098	-0.091
**92**	-0.252	-0.021	-0.102	-0.623*	-0.552	-0.552
**94**	-0.175	-0.259	-0.004	-0.091	-0.063	-0.028
**95**	0.231	0.140	-0.151	0.476	0.357	0.336
**96**	0.014	-0.280	-0.448	0.455	0.175	0.287
**98**	0.651*	0.886**	0.533	0.014	0.546	0.357
**99**	0.636*	0.860**	0.378	-0.119	0.462	0.231
**100**	0.135	0.352	0.194	-0.036	0.157	-0.078
**102**	-0.357	-0.224	-0.259	-0.249	-0.182	-0.441
**104**	0.378	0.448	0.147	0.067	0.399	0.098

p-Value corresponds to the significance level of Spearman correlation coefficient indicated as *: significant at $p < 0.05$; **: significant at $p < 0.01$.

Table S11: Spearman correlation coefficients among *broas'* sensorial analysis scores.

	Color	Smell and odor	Taste and aroma	Texture	Global appreciation
**Appearance**	0.874**	0.595*	0.490	0.825**	0.699*
**Color**		0.522	0.203	0.685*	0.503
**Smell and odor**			0.368	0.525	0.494
**Taste and aroma**				0.739**	0.897**
**Texture**					0.874**

p-Value corresponds to the significance level of Spearman correlation coefficient indicated as *: significant at $p < 0.05$; **: significant at $p < 0.01$.

References:

1. Birch, A.N.; Petersen, M.A.; Hansen, Å.S. The Aroma Profile of Wheat Bread Crumb Influenced by Yeast Concentration and Fermentation Temperature. *LWT-Food Sci. Technol.* **2013**, *50*, 480–488.
2. Birch, A.N.; Petersen, M.A.; Hansen, Å.S. REVIEW: Aroma of Wheat Bread Crumb. *Cereal Chem.* **2014**, *91*, 105–114.
3. Bredie, W.L.P.; Mottram, D.S.; Guy, R.C.E. Aroma Volatiles Generated during Extrusion Cooking of Maize Flour. *J. Agric. Food Chem.* **1998**, *46*, 1479–1487.
4. Pico, J.; Bernal, J.; Gómez, M. Wheat Bread Aroma Compounds in Crumb and Crust: A Review. *Food Res. Int.* **2015**, *75*, 200–215.
5. Moskowitz, M.R.; Bin, Q.; Elias, R.J.; Peterson, D.G. Influence of Endogenous Ferulic Acid in Whole Wheat Flour on Bread Crust Aroma. *J. Agric. Food Chem.* **2012**, *60*, 11245–11252, doi:10.1021/jf303750y.
6. Vogel, J.T.; Tan, B.-C.; McCarty, D.R.; Klee, H.J. The Carotenoid Cleavage Dioxygenase 1 Enzyme Has Broad Substrate Specificity, Cleaving Multiple Carotenoids at Two Different Bond Positions. *J. Biol. Chem.* **2008**, *283*, 11364–11373.
7. Simkin, A.J.; Schwartz, S.H.; Auldridge, M.; Taylor, M.G.; Klee, H.J. The Tomato Carotenoid Cleavage Dioxygenase 1 Genes Contribute to the Formation of the Flavor Volatiles Β-ionone, Pseudoionone, and Geranylacetone. *plant J.* **2004**, *40*, 882–892.
8. Hansen, A.; Schieberle, P. Generation of Aroma Compounds during Sourdough Fermentation: Applied and Fundamental Aspects. *Trends Food Sci. Technol.* **2005**, *16*, 85–94.
9. Berger, R.G. *Flavours and Fragrances: Chemistry, Bioprocessing and Sustainability*; Springer Science & Business Media, 2007; ISBN 3540493395.
10. Bastos, D.M.; Monaro, É.; Siguemoto, É.; Séfora, M.; Markowicz, D.; Monaro, E.; Siguemoto, E.; Sefor, M. Maillard Reaction Products in Processed Food: Pros and Cons. *Food Ind. Process. - Methods Equip.* **2012**, doi:10.5772/31925.
11. Pétel, C.; Onno, B.; Prost, C. Sourdough Volatile Compounds and Their Contribution to Bread: A Review. *Trends Food Sci. Technol.* **2017**, *59*, 105–123.
12. Purlis, E. Browning Development in Bakery Products–a Review. *J. Food Eng.* **2010**, *99*, 239–249.
13. Martins, S.I.F.S.; Jongen, W.M.F.; Van Boekel, M.A.J.S. A Review of Maillard Reaction in Food and Implications to Kinetic Modelling. *Trends Food Sci. Technol.* **2000**, *11*, 364–373.
14. Bailey, M.E.; Gutheil, R.A.; Hsieh, F.-H.; Cheng, C.-W.; Gerhardt, K.O. Maillard reaction volatile compounds and color quality of a whey protein concentrate—corn meal extruded product. In; ACS Publications, 1994 ISBN 1947-5918.
15. Rizzi, G.P. The Strecker Degradation of Amino Acids: Newer Avenues for Flavor Formation. *Food Rev. Int.* **2008**, *24*, 416–435.
16. Jensen, S.; Oestdal, H.; Skibsted, L.H.; Larsen, E.; Thybo, A.K. Chemical Changes in Wheat Pan Bread during Storage and How It Affects the Sensory Perception of Aroma, Flavour, and Taste. *J. Cereal Sci.* **2011**, *53*, 259–268.
17. Pozo-Bayón, M.A.; Guichard, E.; Cayot, N. *Flavor Control in Baked Cereal Products*; 2006; Vol. 22; ISBN 8755912060.
18. Cho, I.H.; Peterson, D.G. Chemistry of Bread Aroma: A Review. *Food Sci. Biotechnol.* **2010**, *19*, 575–582.
19. Belitz, H.-D.; Grosch, W.; Schieberle, P. Cereals and Cereal Products. *Food Chem.* **2009**, 670–745.
